# Non-classical neutrophil extracellular traps induced by PAR2-signaling proteases

**DOI:** 10.1038/s41419-025-07428-z

**Published:** 2025-02-19

**Authors:** Danuta Bryzek, Anna Gasiorek, Dominik Kowalczyk, Michal Santocki, Izabela Ciaston, Ewelina Dobosz, Elzbieta Kolaczkowska, Katarzyna Kjøge, Tomasz Kantyka, Maciej Lech, Barbara Potempa, Jan J. Enghild, Jan Potempa, Joanna Koziel

**Affiliations:** 1 Microbiology Department, Faculty of Biochemistry Biophysics and Biotechnology, Jagiellonian University, Kraków, Poland; 2 Department of Experimental Hematology, Institute of Zoology and Biomedical Research, Jagiellonian University, Krakow, Poland; 3 Department of Molecular Biology and Genetics, Aarhus University, Aarhus, Denmark; 4 MCB, Jagiellonian University, Krakow, Poland; 5 LMU Hospital, Medizinische Klinik und Poliklinik IV, Ludwig-Maximilians University, Munich, Germany; 6 Department of Oral Immunology and Infectious Diseases, School of Dentistry, University of Louisville, Louisville, Kentucky USA

**Keywords:** Coagulation system, Immune cell death

## Abstract

Neutrophil extracellular traps (NETs) are associated with diseases linked to aberrant coagulation. The blood clotting cascade involves a series of proteases, some of which induce NET formation via a yet unknown mechanism. We hypothesized that this formation involves signaling via a factor Xa (FXa) activation of the protease-activated receptor 2 (PAR2). Our findings revealed that NETs can be triggered in vitro by enzymatically active proteases and PAR2 agonists. Intravital microscopy of the liver vasculature revealed that both FXa infusion and activation of endogenous FX promoted NET formation, effects that were prevented by the FXa inhibitor, apixaban. Unlike classical NETs, these protease-induced NETs lacked bactericidal activity and their proteomic signature indicates their role in inflammatory disorders, including autoimmune diseases and carcinogenesis. Our findings suggest a novel mechanism of NET formation under aseptic conditions, potentially contributing to a self-amplifying clotting and NET formation cycle. This mechanism may underlie the pathogenesis of disseminated intravascular coagulation and other aseptic conditions.

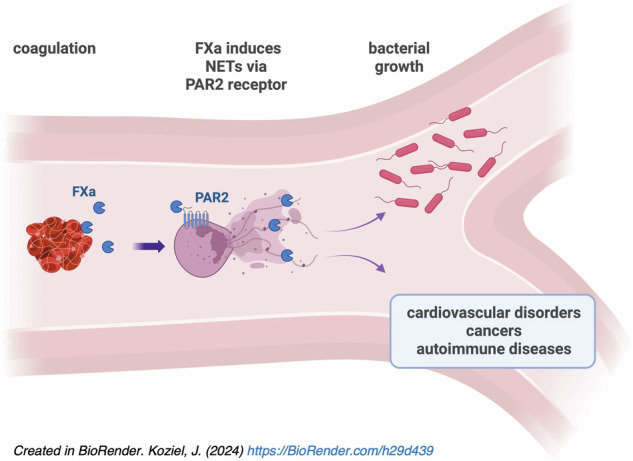

## Introduction

Neutrophil extracellular traps (NETs) are chromatin structures decorated with histones and other proteins that are released by neutrophils as an antibacterial mechanism that plays an important role in innate immunity [1]. Many pathogens and virulence factors are NET inducers, such as bacterial lipopolysaccharides (LPS) and exotoxins [1–3]. However, NETs are also produced during non-infectious diseases, including autoimmune disorders such as rheumatoid arthritis, psoriasis, systemic lupus erythematosus and arteriosclerosis [4]. Many endogenous NET inducers have been identified, including chemokines (IL-8, CXCL1, CXCL2, CXCL3), interferons (IFN-α, IFN-γ), anaphylatoxins (C5a) and growth factors (GM-CSF) [5–8]. Furthermore, alarmins such as β-defensin 1 (hBD-1) [9] and the high mobility group box 1 protein (HMGB1) are known to trigger NETs [10, 11]. Finally, components of the coagulation system, such as von Willebrand factor (VWF) [12], platelet factor 4 (PF4) [12, 13] and coagulation factor XII (FXII) are also recognized as inducers of NETs [14].

Microbial proteases are implicated in NET formation [2, 15]. Arginine-specific gingipains secreted by the periodontal pathogen *Porphyromonas gingivalis* trigger NETs via the activation of protease-activated receptor 2 (PAR2) [2]. This implies that endogenous host proteases may also induce NET, especially in the inflammatory environment, where their activity is enhanced by neutrophil degranulation, proteolytic cascades (coagulation, fibrinolysis, and kinin generation), and the secretion of proteases from immune and structural cells [16]. No human proteases are yet known to directly induce NETosis, but many activate PAR-dependent signaling pathways in different cell types and may provide insight into the endogenous triggers of NETs.

The human protease-activated receptors (PAR1–4) belong to the G protein-coupled receptor (GPCR) family [17] and are activated by proteolytic cleavage at a specific peptide bond in their N-terminal domain. This uncovers a new N-terminal motif that acts as a tethered ligand and interacts with an extracellular domain of the receptor. The canonical pathway induced by PAR activation engages PLC/Ca^2+^/PKC endocytosis and endosomal ERK signaling [18, 19]. Alternatively, proteolysis at the N-terminus but away from the canonical site generates a tethered ligand that does not result in endocytosis, but instead activates adenylyl cyclase and ultimately leads to the activation of PKA [18].

Neutrophils predominantly express PAR2, whose role in inflammatory diseases has been extensively studied but to the best of our knowledge not in the context of NET formation in response to receptor activation by endogenous proteases. We therefore investigated human proteases known to signal via PARs, focusing on PAR2 to induce NET formation in the sterile environment of chronic inflammation. Here we show for the first time the formation of a new category of NETs induced by endogenous proteases via a non-classical mechanism. Our results might explain the accumulation of these structures under aseptic conditions due to excessive blood clotting associated with inflammation.

## Results

### Host proteases promote the formation of NETs via PAR2 activation

**Fig. 1:**
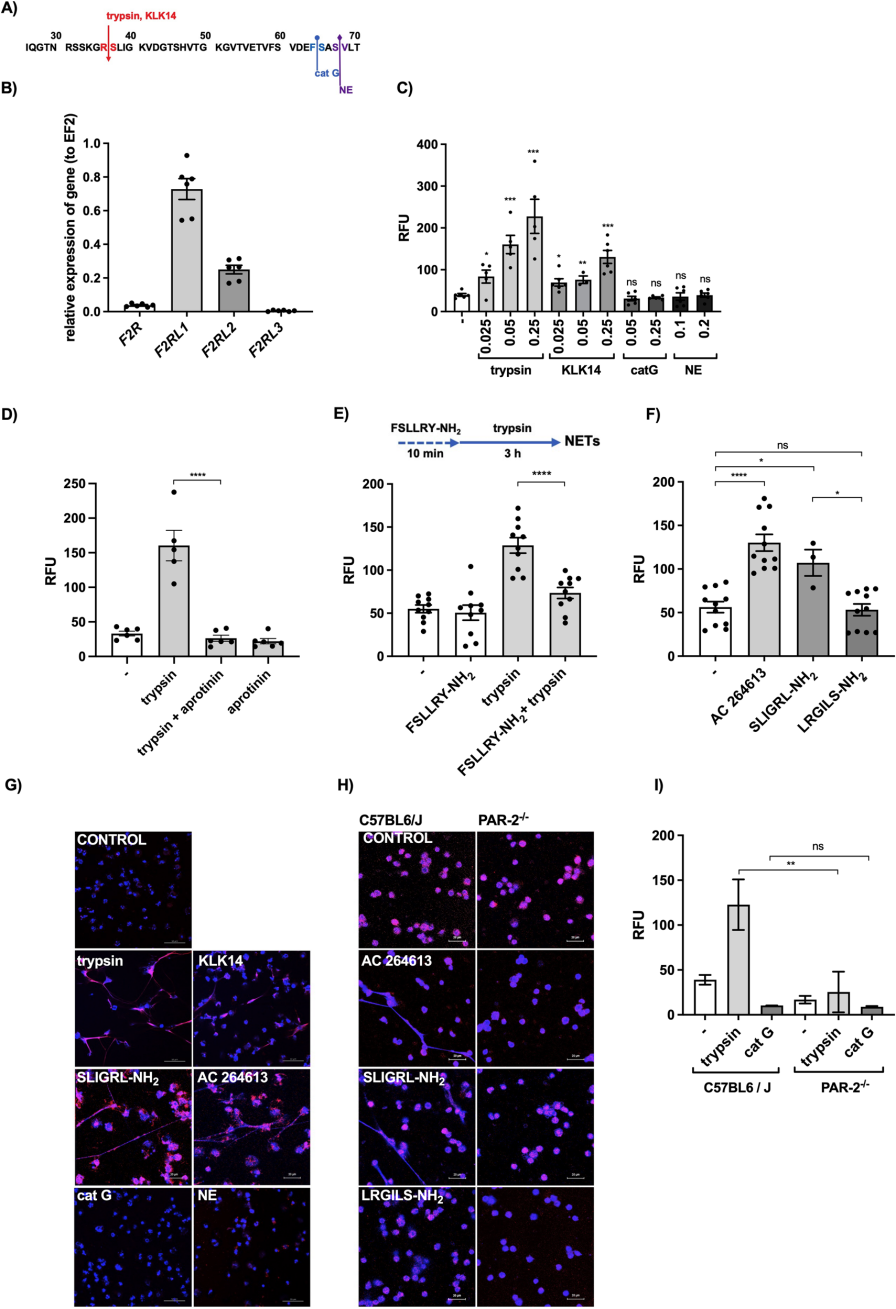
NET formation is triggered by PAR2-activating proteases and small-molecule PAR2 agonists but not by proteases that cleave PAR2 at non-canonical sites. **A** PAR2 sequence showing cleavage sites for human proteases. **B** Relative expression levels of *PAR1* (*F2R*), *PAR2* (*F2RL1*), *PAR3* (*F2RL2*) and *PAR4* (*F2RL3*) mRNA in human neutrophils. **C** The amount of extracellular DNA released by neutrophils 3 h post-incubation with trypsin, kallikrein 14 (KLK14), cathepsin G (catG) and neutrophil elastase (NE) at the indicated concentrations (μM) based on SytoxGreen staining (RFU = relative fluorescence units). **D** The amount of extracellular DNA released by neutrophils 3 h post-incubation with 0.05 μM trypsin, trypsin pre-incubated in a 1:1 ratio with aprotinin based on SytoxGreen staining. **E** The amount of extracellular DNA released by neutrophils 3 h post-stimulation with trypsin (0.05 μM) after pre-incubation with 100 μM FSLLRY-NH_2_ for 10 min. **F** The amount of extracellular DNA released by neutrophils 3 h post-stimulation with 100 μM AC 264613 or SLIGRL-NH_2_ (PAR2 agonists) or LRGILS-NH_2_ (reversed amino acid sequence control peptide for SLIGRL-NH_2_) based on SytoxGreen staining. **G** NET structures visualized by a confocal laser scanning microscopy. DNA is stained with Hoechst 33342 (blue), and NE is shown in red (scale bars = 50 μm). **H** Visualization of NETs formed by peritoneal neutrophils from wild-type and *PAR2*^–/–^C57BL6/J mice after treatment with PAR2 agonists, visualized by a confocal laser scanning microscopy. **I** Peritoneal neutrophils from wild-type and *PAR2*^–/–^ C57BL6/J mice were incubated with trypsin and catG (0.25 μM). Statistical significance was determined by applying an unpaired *t*-test (**C**) or by one-way ANOVA (**D–F, I**) followed by Tukey’s multiple comparisons test. Data are means (± SEM) with a minimum of *n* = 3 (**C**–**F**) or *n* = 2 (**I**) independent experiments using neutrophils from different healthy donors (*P < 0.05, **P < 0.01, ***P < 0.001, ****P < 0.0001; ns non-significant).

We previously showed that the *P. gingivalis* protease gingipain R induces NETs in a PAR2-dependent manner [2]. To determine whether the formation of NETs in response to proteases is a general phenomenon, we tested the host enzymes trypsin and KLK14. These enzymes signal via PAR2 by removing the extracellular N-terminus at a canonical site (Arg36/Ser37), exposing a tethered ligand in the new N-terminal sequence (Fig. 1A) [20]. We also applied cathepsin G and neutrophil elastase, which cleave PAR2 at non-canonical sites and thus inactivate the receptor (Fig. 1A) [20]. Using neutrophils that highly express PAR2 (Fig. 1B, Fig. S1) [21], we found that only the first group of enzymes generated NETs in a concentration-dependent manner (Fig. 1C). The process was prevented by pre-incubation with protease inhibitors (Fig. 1D) and/or a PAR2 antagonist (Fig. 1E), confirming that proteolytic activity and PAR2 activation are necessary to trigger NETs.

PAR2 can also be activated by small-molecule agonists mimicking the insertion of the tether ligand [22]. We, therefore, confirmed the induction of NETs via PAR2 using the synthetic PAR2 agonists AC 264613 and SLIGRL-NH_2_, whereas the control peptide with a reversed amino acid sequence (LRGILS-NH_2_) did not stimulate the release of NETs (Fig. 1F). The formation of NETs in response to proteases and PAR2 agonists was visualized by confocal microscopy, revealing characteristic extracellular DNA fibers colocalized with elastase when neutrophils were treated with trypsin, KLK14 or either of the PAR2 agonists, but not with cathepsin G or neutrophil elastase (Fig. 1G). Finally, the role of the PAR2 activation was confirmed using neutrophils from the *PAR2*^–/–^ mouse strain, which did not respond to trypsin or the PAR2 agonists (Fig. 1H, I). These results show decisively that host proteases promote the formation of NETs in both human and murine neutrophils via the proteolytic activation of PAR2.

### Coagulation factor Xa activates NETs

**Fig. 2:**
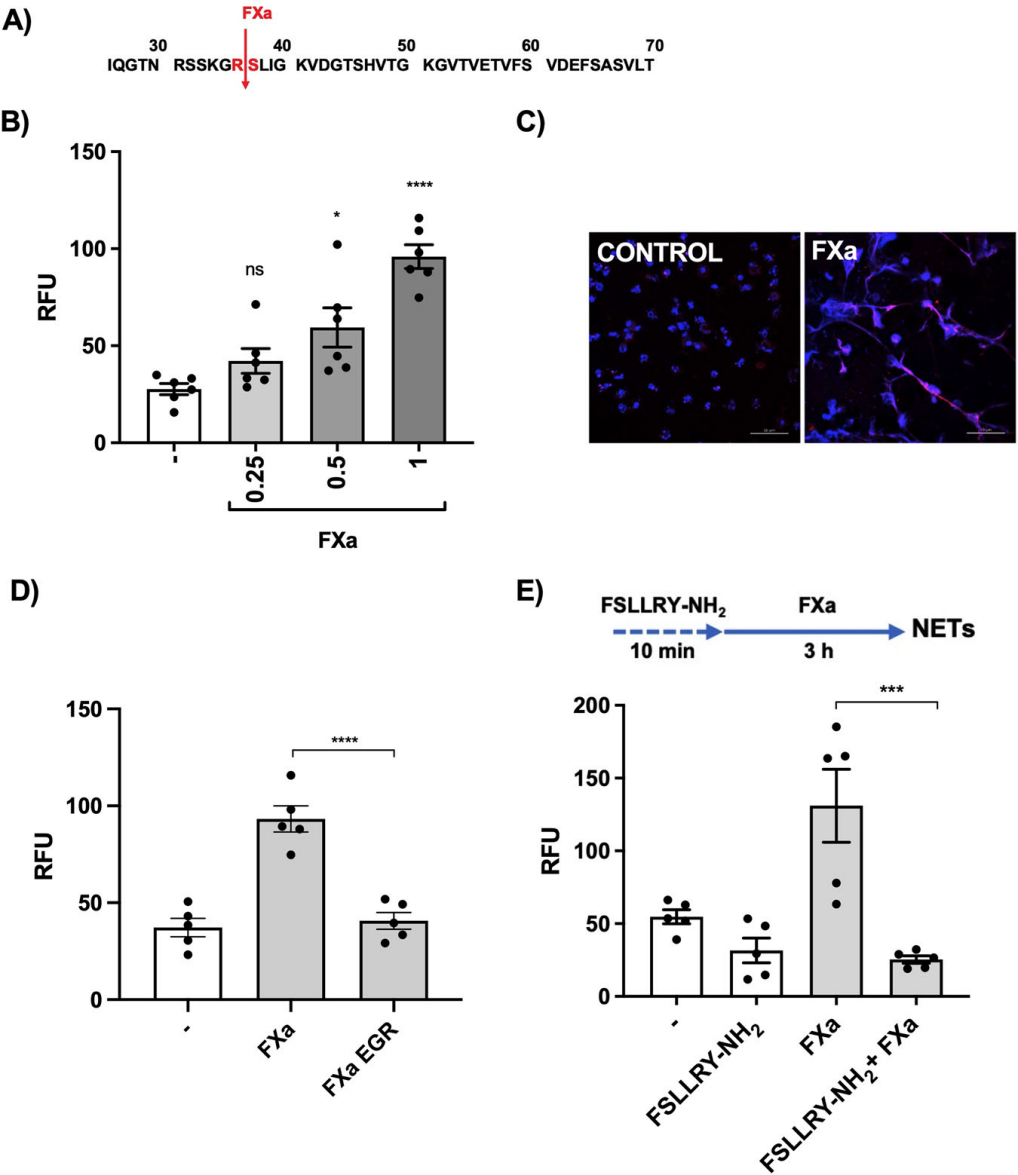
NET formation is triggered by PAR2-activating FXa. **A** PAR2 sequence showing cleavage sites for FXa. **B** The amount of extracellular DNA released by neutrophils 3 h post-incubation with FXa at the indicated concentrations (μM) based on SytoxGreen staining. **C** NET structures visualized by a confocal laser scanning microscopy. DNA is stained with Hoechst 33342 (blue), and NE is shown in red (scale bars = 50 μm). **D** The amount of extracellular DNA released by neutrophils 3 h post-incubation with FXa and FXa EGR native protein (active site irreversibly blocked by 1 μM of the chloromethylketone tripeptide inhibitor EGRck) based on SytoxGreen staining. **E** The amount of extracellular DNA released by neutrophils 3 h post-stimulation with FXa (1 µM) after pre-incubation with 100 μM FSLLRY-NH_2_ for 10 min. Statistical significance was determined by applying an unpaired *t*-test (**B**) or by one-way ANOVA (**D, E**) followed by Tukey’s multiple comparisons test. Data are means (± SEM) with a minimum of *n* = 3 (**B, D**, **E**) independent experiments using neutrophils from different healthy donors (*P < 0.05, ***P < 0.001, ****P < 0.0001; ns non-significant).

Coagulation factor Xa is one of the physiological activators of PAR2 (Fig. 2A) [23], which, together with FVIIa, is considered crucial in extensive crosstalk between coagulation and inflammatory responses [24]. We found, that FXa promotes dose-dependent NET formation (Fig. 2B, C). The observed effect depends on the proteolytic activity of FXa (Fig. 2D) and is limited in the presence of PAR2 antagonist (Fig. 2E). To determine whether the formation of NETs is induced during coagulation, we applied two potent activators of FX that induce clotting [25, 26]: Russell viper (*Daboia russelli*) venom (RVV-X), and RgpA, a *P. gingivalis* cysteine protease (Fig. S2A, B). Clotting made it impossible to image NET formation in the ex vivo setting, so we can only show that FXa generated by the pre-incubation of FX with RVV-X or RgpA stimulated NET formation (Fig. S2C, D). Notably, we used 10 nM RgpA, the concentration much lower than needed to induce NETs by direct action on PAR2 (50 nM) [2]. NET formation was prevented by the pre-treatment of RgpA with Kyt-1, a specific inhibitor of Rgp gingipains, but was unaffected if Kyt-1 was added after the treatment of FX with RgpA (Fig. S2D). These data showed for the first time that FXa is a potent inducer of NETs and that the exogenous activation of FX leads to the generation of NETs in vitro. Since NET formation stimulated by FXa is associated with enhanced clotting (Fig. S2E), it may trigger a feed-forward loop of pathological outcomes involved in inflammatory diseases.

### FXa induces NETs in vivo in the liver microcirculation

**Fig. 3:**
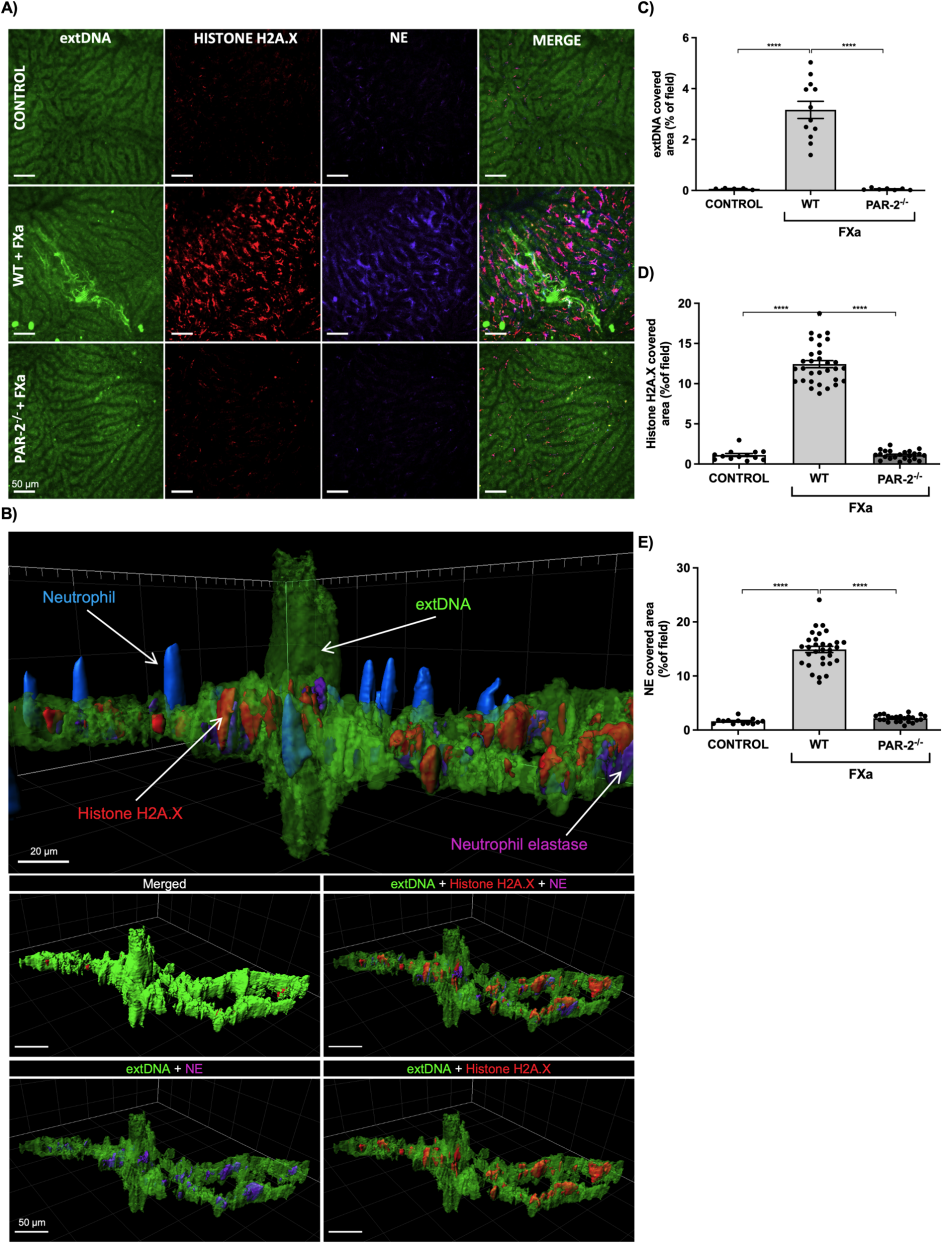
FXa induces NET formation in the liver vasculature and is dependent on PAR2 expression. **A** Representative images of NETs acquired by spinning disk intravital microscopy (SD-IVM) in untreated (control) wild-type (WT) mice as well as wild-type and *PAR2*^–/–^ mice injected with murine FXa 4 h before imaging. Extracellular DNA (extDNA) was stained with SytoxGreen (bright green), histone H2A.X was stained with an AlexaFluor 555-conjugated antibody (red) and neutrophil elastase was stained with an AlexaFluor 647-conjugated antibody (blue). The images from each channel were overlaid (scale bars = 50 μm). **B** Representative 3D conformation of the NET structure after FXa treatment in WT mice. **C–E** Quantitative analysis of NETs in liver sinusoids: area (%) covered by (**C**) extDNA, (**D**) histone H2A.X and (**E**) neutrophil elastase (NE). **C–E** Statistical significance was evaluated by one-way ANOVA followed by Tukey’s multiple comparisons test. Data are means (± SEM) from *n* ≥ 3 independent experiments (****P < 0.0001).

The activation of FX is a key event in the blood clotting pathway, but excessive FXa activity can trigger many signaling pathways via PARs and other receptors. FXa can thus cause several inflammatory diseases [27], each of which is associated with NETs generation [4, 28]. The formation of NETs via FXa-dependent PAR2 signaling has not been studied in detail, so we assessed the ability of FXa to induce NETs in vivo. We used multichannel SD-IVM to visualize extracellular DNA colocalized with elastase and histones in the liver microcirculation following the *i.p*. administration of exogenous FXa. We then compared the neutrophil response to FXa in the liver microcirculation of wild-type and *PAR2*^–/–^ C57BL6/J mice. FXa generated NETs that were localized in the liver vessels of wild-type but not PAR2-deficient mice (Fig. 3A). Visualization of the 3D structure of NETs in wild-type mice after FXa treatment revealed neutrophils surrounded by extracellular DNA, along with neutrophil elastase and histone H2A.X (Fig. 3B). We determined the quantity of NET components relative to the number of neutrophils, which confirmed that the activation of PAR2 following treatment with FXa caused NET formation in the liver microcirculation (Fig. 3C-E). These results show that activation of the coagulation cascade in vivo can lead to the formation of intravenous NETs and that both FXa and PAR2 are required.

### Induction of intravascular coagulation is associated with NET formation

Although NETs induced by FXa strongly suggest a causative link between coagulation and NET formation, we were determined to show this link more directly. We, therefore, designed experiments in which coagulation in the liver microcirculation was induced by the infusion of pure RgpA directly into the liver vessel. The application of RVV-X to trigger intravascular NETs was not possible due to the lethal effect of the venom [29].

**Fig. 4:**
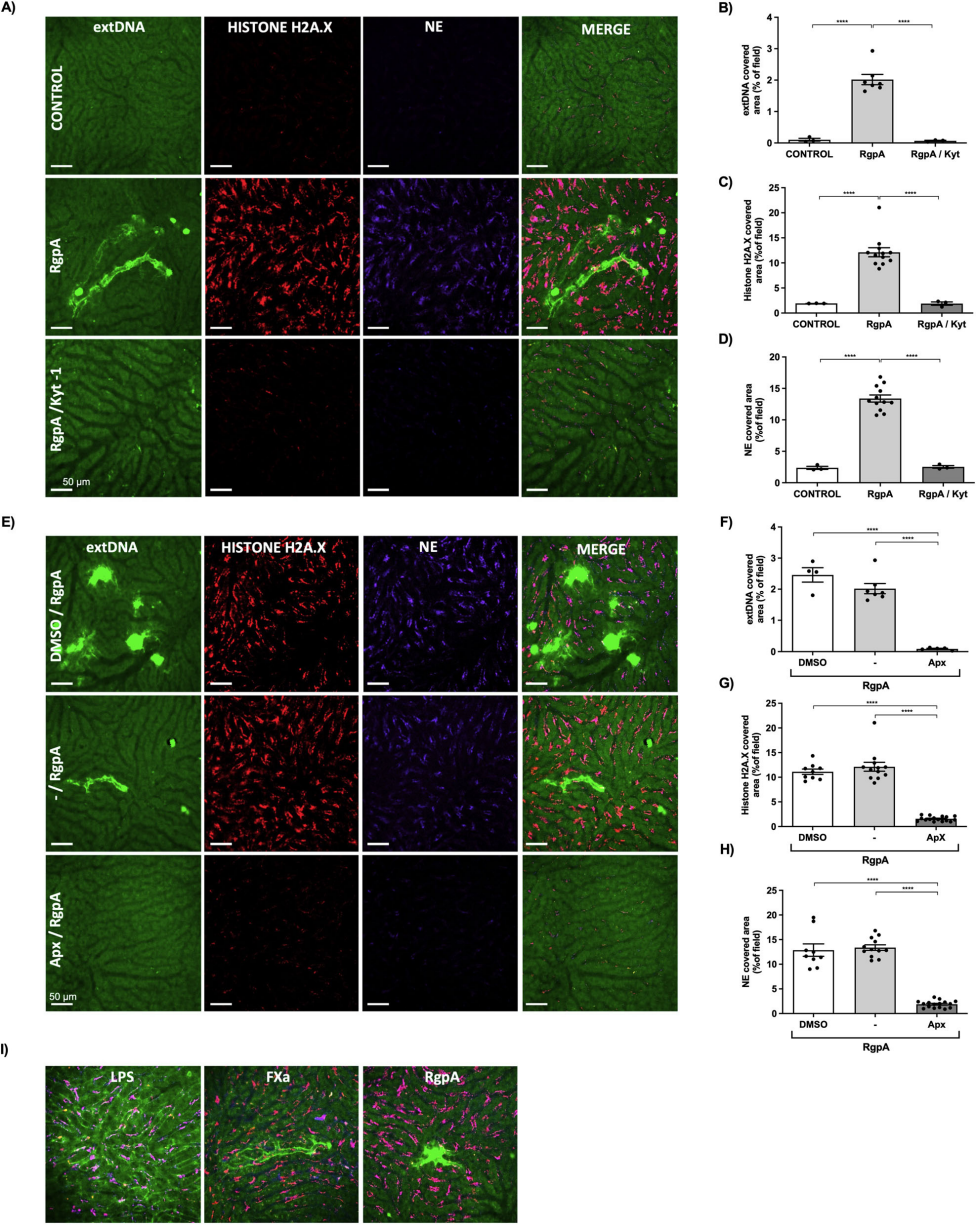
Induction of coagulation by RgpA in the liver vasculature is associated with NET formation. **A, E** NET formation in the liver vasculature was estimated by SD-IVM in wild-type (WT) mice injected with RgpA 4 h before imaging. **A** Kyt-1, a gingipain-specific inhibitor was added immediately after activation of RgpA, before its administration to the mice, and (**E**) apixaban, which inhibits FXa, was administered *i.p*. 30 min before RgpA. DMSO was used as a control for apixaban. Extracellular DNA (extDNA) was stained with SytoxGreen (green bright), histone H2A.X with an AlexaFluor 555-conjugated antibody (red) and neutrophil elastase was stained with an AlexaFluor 647-conjugated antibody (blue). The images from each channel were overlaid (scale bars = 50 μm). **B–D, F–H** Quantitative analysis of NETs in the liver sinusoids: area (%) covered by (**B, F**) extDNA, (**C, G**) histone H2A.X, and (**D, H**) neutrophil elastase (NE). **I** The appearance of NETs triggered by LPS, FXa and RgpA as inducers of coagulation. **B–D, F–H** Statistical significance was evaluated by one–way ANOVA followed by Tukey’s multiple comparisons test. Data are means (± SEM) of *n* ≥ 3 separate experiments (****P < 0.0001).

In agreement with our in vitro results (Fig. S2), RgpA triggered NET formation in the liver microcirculation (Fig. 4A-D), and the effect was dependent on gingipain activity because it could be prevented by pre-incubation of the protease with Kyt-1 (Fig. 4A-D). Given that RgpA directly signals through PAR2 [30] and can cause NETs at higher concentrations [2], we confirmed that formation of NETs observed in vivo was not caused directly by RgpA but by the FXa generated by RgpA. This was achieved by applying apixaban, a selective inhibitor of FXa that does not affect gingipain activity (Fig. S3). The formation of NETs was abolished in mice treated with apixaban (Fig. 4E-H), thus confirming that intravascular NETs result from the direct action of FXa. Furthermore, this result argues against the involvement of plasma kallikrein, activated protein C and thrombin in the coagulation pathway, all generated by RgpA in human plasma [31–33] and all known to signal via PAR2 [22, 34].

Finally, we compared the pattern of NETs formed in response to FXa and LPS because the latter promotes NET formation in liver sinusoids during endotoxemia [35]. We found that, in contrast to the diffuse pattern of NETs induced by LPS (Fig. 4I), NETs triggered by exogenous or endogenous FXa led to the formation of well-defined but limited clusters of dense NETs (Fig. 4I), apparently due to blood clotting in the liver vasculature. These results show unambiguously that intravascular coagulation is associated with NET formation promoted by FXa.

### Biochemical and functional characterization of protease-derived NETs

**Fig. 5:**
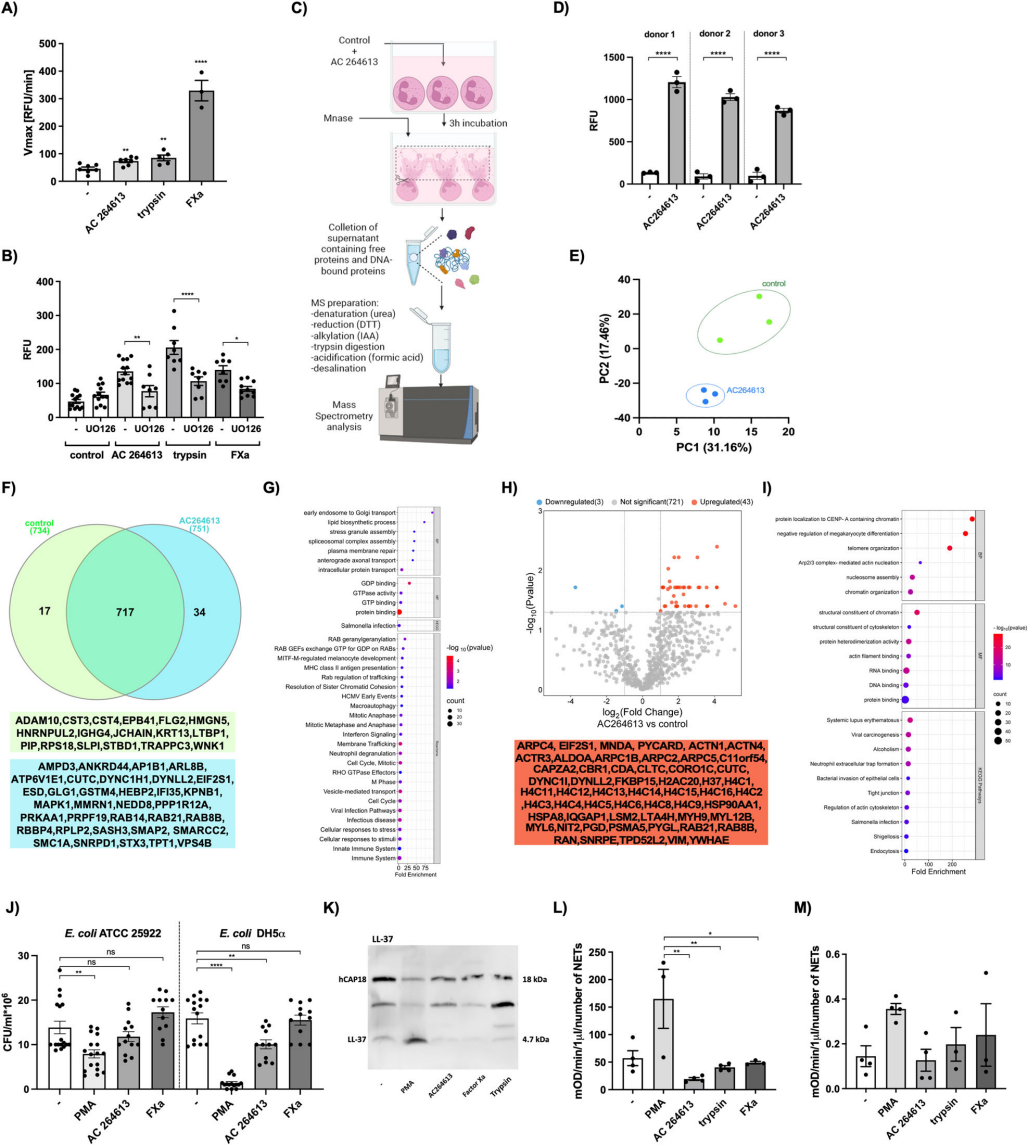
Biochemical and functional characterization of protease-derived NETs. **A** Calcium mobilization in neutrophils exposed to AC 264613, trypsin, or FXa. **B** The amount of extracellular DNA estimated by SytoxGreen staining in neutrophils pre-treated for 5 min with the ERK1/2 inhibitor UO126 before stimulation with AC 264613, trypsin or FXa for 3 h. **C** Scheme of NETs samples preparation for mass spectroscopy analysis. **D** Formation of NETs released by neutrophils isolated from 3 independent donors. The amount of extracellular DNA released by neutrophils 3 h post-incubation with AC 264613 (100 μM) based on SytoxGreen staining (RFU = relative fluorescence units). **E** Principal component analysis (PCA) showing differences between the two analyzed groups of proteomes (control and AC 264613). **F** Venn diagram presenting all identified proteins in control NETs and after AC 264613 stimulation (FDR = 1%, *n* ≥ 2 unique peptides). Gene names for unique peptides identified for control and AC 264613 are tabulated. **G** List of GO Biological Processes (BP) Molecular Function (MF) KEGG Pathways (KEGG) and Reactome Pathways (Reactome) for unique 34 proteins quantified in AC 264613 induced NETs of at least one donor obtained using the Database for Annotation, Visualization and Integrated Discovery - DAVID database (*p* value = 0.05). **H** Volcano plot of differently abundant proteins after AC 264613 stimulation. Gene names for proteins with increased abundance are tabulated. **I** List of GO Biological Processes (BP) Molecular Function (MF) KEGG Pathways for 42 proteins with increased abundance found in AC 264613 induced NETs obtained using DAVID database (*p* value with Bonferroni posttest = 0.05). **J** Bactericidal activity of NETs induced by PAR2 activators. *E. coli* cultures were mixed with NETs generated by exposure to PMA (25 nM), AC 264613 (100 μM) and FXa (1 μM) or serum-free DMEM collected from neutrophils. After incubation for 2 h, the bacteria were plated and the number of colonies (CFUs) was determined. **K** The presence of LL-37 within NETs generated by PMA, AC 264613, FXa or trypsin was visualized by immunoblot analysis 3 h poststimulation. A representative immunoblot is shown. **L,** **M** Enzymatic activity of human neutrophil elastase (**L**) and cathepsin G (**M**) in NETs generated by PMA, AC 264613, FXa or trypsin. **A, D, J, L** Statistical significance was evaluated by applying an unpaired *t*-test, or (**B**) by one- way ANOVA followed by Tukey’s multiple comparisons test. **A**–**M** Data are means ( ± SEM) of *n* ≥ 3 independent experiments (*P < 0.05, **P < 0.01, ***P < 0.001, ****P < 0.0001; ns non-significant).

Our studies conclusively demonstrated the formation of NETs triggered by proteases in vivo, but did not answer the question regarding the biochemical nature and biological functions of these structures. To address this question, we have first investigated the signaling pathways activated by proteases during the formation of NETs. Initially, we examined the activation of MEK/ERK and PI3K-AKT a well-known components of the PAR2 signaling pathways [36] by FXa and AC 264613 (Fig. S4). We confirmed intracellular calcium release following the treatment of neutrophils with FXa, trypsin, and AC264613 (Fig. 5A), a hallmark of PAR2 signaling [20]. Moreover, we found that NET formation was also dependent on the activation of ERK (Fig. 5B). We have, therefore, shown that the canonical signal transduction pathway mediated by the activation of PAR2 is mechanistically responsible for NETs formation induced by proteases.

As biological functions of NETs depend on the composition of proteins decorating the chromatin structure [37], we performed a detailed proteomic analysis of PAR2-dependent NETs (Fig. 5C). To this end, samples were prepared from neutrophils of three healthy blood donors, stimulated with AC 264613 to stimulate NET formation or left untreated (control) (Fig. 5D), and subjected to quantitative mass spectrometry analysis. In total 768 proteins were found within a 1% false discovery rate (FDR) and number of unique peptides ≥2 (Table S1). Principal component analysis (PCA) displays the clustering of replicates and separation between experimental groups (control vs AC 264613) indicating their different expression (Fig. 5E). Venn diagram shows 34 proteins exclusively quantified in at least one of the three donors after NET induction by AC 264613 compared to the untreated control (Fig. 5F). Among them are those crucial for endosome trafficking, PAR2 signaling (Rab proteins) and cellular stress response (Fig. 5G). Volcano plot analysis shows different abundance of 46 proteins; among them, 43 were significantly more abundant after NET induction by AC 264613 (Fig. 5G). Among the top Gene Ontology (GO) biological process terms enriched were the organization of chromatin and interactions with nucleic acids and proteins (Fig. 5I). Among KEGG pathways are the formation of NETs, autoimmune diseses and carcinogenesis (Fig. 5I) (DAVID database). Proteomic data confirm the role of PAR2 signaling in NET formation. Then, to further explore the biological functions of protease-derived NETs, we probed them for antimicrobial immune defense function. Initially, we examined their antibacterial potential by comparing them to PMA-induced NETs. Surprisingly, we found reduced bactericidal activity in NETs induced by proteases (Fig. 5J). We attributed this result to the absence of the antimicrobial peptide LL-37 from the protease-derived NETs (Fig. 5K) and the low activity of major antibacterial enzymes, including elastase and cathepsin G, in the protease-derived NETs when compared to PMA-induced NETs (Fig. 5L, M). Collectively, we showed the unique biological properties of PAR-dependent NETs, proposing that protease-induced NETs should be classified as a novel type of NETs with a potentially fundamental role in regulating aseptic inflammation.

## Discussion

NETs evolved as an innate immunity mechanism to immobilize and inactivate pathogens [1, 38]. However, the excessive induction of NETs and/or slow clearance by nucleases and phagocytosis is pathological [39]. This can lead to autoimmune diseases that reflect the breakdown of immunological tolerance to self-antigens massively released from netting granulocytes [40]. The deposition of NETs also promotes tumor growth and progression, metastasis, and cancer-associated thrombosis [41]. The accumulation of NETs is also associated with blood vessel occlusion and has been identified in atherosclerotic lesions and arterial thrombi [42]. It is, therefore, important to identify factors that trigger NET formation during aseptic inflammation.

NETs were only recently classified as an important component of the coagulation cascade and are proposed to trigger atherosclerotic plaque formation and arterial thrombosis. The extruded DNA network binds platelets, enabling their aggregation and activating them via histone H4 [43]. Furthermore, once activated by H4, platelets secrete polyP, which induces coagulation by FXII [35, 44]. NETs also initiate coagulation by binding to and facilitating the activation of FXII [45] and tissue factor, which initiate the extrinsic (FXa) and intrinsic (FVIIa) coagulation cascades [46, 47]. Interestingly, the role of NETs in coagulation is bidirectional. Platelets induce the formation of NETs on contact with neutrophils [48] but the molecular mechanism remained unknown until this study, which revealed for the first time that FXa is a potent inducer of NETs in vitro and in vivo. Moreover, other components of the coagulation cascade (FVIIa, activated protein C, and thrombin) and the fibrinolysis pathway (plasmin) also activate PAR2 signaling [22, 23] and may also promote NETs. This is supported by the clusters of NETs visible in the liver circulation, apparently limited to intravenous clots, distinguishing these structures from the diffused NETs induced by LPS (Fig. 4). Finally, we cannot exclude the possibility that other PARs may be involved. However, PAR1 and PAR4 are expressed at much lower levels than PAR2 on neutrophils (Fig. 1A) and the more abundant PAR3 forms heterodimers with PAR1 [49, 50]. Therefore, unless PAR3 has an unknown function involving crosstalk with PAR2, we assume that PAR2 signaling triggers protease-induced NETs associated with coagulation. This novel finding mechanistically explains the link between coagulation and NET formation and confirms the bidirectional mechanism in which coagulation and PAR2-induced NETs form a feedforward loop.

In rheumatoid arthritis, NETs are released into the synovial fluid, where they are found with anti-citrullinated protein antibodies (ACPA). Citrullinated and carbamylated antigens are implicated in the pathogenesis of rheumatoid arthritis, so in-depth studies of the NET proteome are needed, given that PAR2-activating enzymes are also found in the synovium [51]. These include coagulation factors and mast cell tryptase, which exacerbates synovitis in rheumatoid arthritis and osteoarthritis by signaling via PAR2 [52, 53]. Activation of the coagulation pathway by gingipains may also be relevant because *P. gingivalis*, the keystone pathogen in periodontitis, is considered the etiological factor that promotes rheumatoid arthritis [54].

Many of the proteases present in tumors activate PAR signaling, including elevated levels of KLK14 in tumors and inflammatory skin diseases such as the Netherton syndrome [55–57]. Moreover, the tumorigenic role of PAR2 in response to trypsin has been demonstrated in colon cancer, ovarian cancer, breast cancer, and colorectal cancer [58–61]. FXa promotes tumor migration and invasion in breast cancer [62]. Importantly, neutrophils are key innate immune effector cells in cancer, explaining the presence of NETs in Ewing’s sarcoma [63], breast cancer [64], pancreatic cancer [65], colorectal cancer [66] and ovarian cancer [67], but their role is not clearly defined. NETs inhibit the proliferation of colon cancer cells and have a cytotoxic effect against melanoma cells [68, 69]. However, they also capture and protect circulating cancer cells from NK cells and CD8^+^ T cells [70, 71]. NETs also promote the awakening of dormant cancer cells [72]. Furthermore, NET-associated proteases promote the remodeling of the extracellular matrix. Accordingly, our results may lead to new research focusing on proteases in the cancer microenvironment that act as NETs inducers, revealing their yet undiscovered role in cancer development and progression. Justification for this research is suggested by the proteome of PAR2-induced NETs.

In summary, we have discovered a new category of non-classical, endogenous protease-triggered NETs, providing insight into the etiology of non-infectious inflammatory diseases, including those related to coagulation, but also autoimmune diseases and carcinogenesis (graphical abstract). Our results provide a broader view of the undoubtedly bidirectional relationship between NETs and coagulation. Most importantly, the new category of NETs reported here for the first time may be considered therapeutic targets for protease inhibitors and/or PAR2 antagonists.

## Materials and Methods

### Isolation of human neutrophils

A fraction enriched in granulocytes was obtained by density gradient centrifugation in lymphocyte separation medium (Pan Biotech). Neutrophils were separated from erythrocytes using 1% polyvinyl alcohol (POCH). After 30 min of sedimentation, the upper layer was harvested and centrifuged (280 × g, 10 min, room temperature) and the residual erythrocytes were lysed in water. Neutrophils were resuspended in serum-free Dulbecco’s modified Eagle’s medium (DMEM) without phenol red (Gibco/Thermo Fisher Scientific). The purity of the human neutrophils was assessed by flow cytometry analysis. Cells were incubated in 0,5% BSA in PBS 1x with addition of PerCP-Cy™5.5 Mouse Anti-Human CD14 Clone MφP9 (BD Pharmingen, cat. no. 562692), FITC anti-human CD15 (SSEA-1) Antibody, Clone HI98 (BioLegend, cat. no. 301904), or CD3 Monoclonal Antibody (UCHT1), APC (eBioscience™, cat. no. 17-0038-42) in 1:100 dilution for 30 minutes in 4 °C. Data was acquired by the BD LSR Fortessa system (Becton Dickinson) and analyzed by BDFACS Diva sofware. The data are presented in the Fig. S5A.

### Sex as a biological variable

Our study examined male and female animals, and similar findings are reported for both sexes. The animals were allocated in experimental groups randomly, within a given phenotype.

### Isolation of murine peritoneal neutrophils

C57BL6/J wild-type and PAR2 knockout (*PAR2*^–/–^) mice, 6–8 weeks old, were injected intraperitoneally (*i.p*.) with 1 ml of 4% sterile thioglycolate (Fluka) to induce peritonitis. After 3 h, peritoneal lavage was performed with 10 ml ice-cold phosphate-buffered saline (PBS; Gibco/Thermo Fisher Scientific). The resulting cell suspension was centrifuged (280 × g, 5 min, room temperature) and the retained erythrocytes were lysed in water. The neutrophils were resuspended in serum-free DMEM without phenol red. The purity of the peritoneal neutrophils was assessed by flow cytometry analysis (Fig. S5B). Cells were incubated in 0,5% BSA in PBS 1x with addition of FITC-conjugated rat anti-mouse Ly-G6 antibody (ThermoFisher Scientific, Invitrogen, cat. no. 1-9668) in 1:200 dilution for 30 minutes in room temperature. Data was acquired by the BD LSR Fortessa system (Becton Dickinson) and analyzed by FlowJo v10 software.

### Isolation of murine bone marrow-derived neutrophils

Bone marrow neutrophils were isolated from 8-week-old wild-type C57BL6/J mice. The isolated femur and tibia were centrifuged (10 000 × g, 40 sec, RT), cells were resuspended in DMEM supplemented with 10% FBS and 1% penicillin/streptomycin and passed through a 40 μm nylon cell strainer. The single cell suspension was centrifuged (350 × g, 5 min, RT), lysed in 0.155 M NH_4_Cl to remove erythrocytes, and centrifuged (350 × g, 5 min, RT) after addition of ice-cold PBS. Collected cells were resuspended in DMEM and passed through a 70 μm nylon cell strainer and centrifuged (350 × g, 5 min, RT). Cells were resuspended in ice-cold PBS 1x supplemented with 2% FBS and 1 mM EDTA. Bone marrow cells were separated using the double gradient with Histopaque 1119 and 1077 (Sigma-Aldrich). After centrifugation (700 × g, 30 min, RT) bottom layer enriched in neutrophils was collected and centrifuged again (350 × g, 10 min, RT). The purity of the bone marrow-derived neutrophils was assessed by flow cytometry analysis (Fig. S5C). Cells were incubated in 0,5% BSA in PBS 1x with addition of FITC-conjugated rat anti-mouse Ly-G6 antibody (ThermoFisher Scientific, Invitrogen, cat. no. 1-9668) and anti-mouse CD11b-PE (ThermoFisher Scientific, Invitrogen, catalog numer 12-0112-82) in 1:200 dilution for 30 minutes in room temperature. Data was acquired by the BD LSR Fortessa system (Becton Dickinson) and analyzed by BDFACS Diva software.

### Quantitative reverse-transcription polymerase chain reaction (qRT-PCR)

Total cellular RNA was extracted from human peripheral neutrophils using TRIzol reagent (Invitrogen/Thermo Fisher Scientific). Briefly, 800 ng of RNA was reverse transcribed with MultiScribe Reverse Transcriptase (Applied Biosystems) in a total volume of 20 μl according to the manufacturer’s instructions. We then amplified 20 ng of the resulting complementary DNA (cDNA) in a 15-μl reaction containing 10 mM of each primer (Table S2) and 1× GoTaq PCR master mix (Promega). The templates were denatured at 95 °C for 5 min followed by 40 amplification cycles (Table S2) and a final elongation step at 72 °C for 10 min. Differences in gene expression were determined using the ΔΔC_T_ method [73] and normalized against the housekeeping gene *EF-2*.

### PAR2 protein expression on the surface of human and mouse neutrophils

The cells (0,2 mln) were left untreated (human and mouse bone marrow-derived PMN), fixed with 4% formaldehyde (5 min) and washed with PBS 1x. Cells were then permeabilized with 0,1% PBS-TritonX-100 for 3 minutes. After washing with PBS 1x, neutrophils were incubated in 0,5% BSA containing 5 μg/ml anti-PAR2 antibody (ab180953, abcam) for 30 minutes at 20 °C. Goat anti rabbit IgG conjugated with APC (cat. no. 111-136-144, 1:1000 dilution, 30 minutes, 20 °C) was used as secondary antibody. Additionally, surface of cells were quenched with trypan blue (0.83 mg/ml) immediatelly before measurment. Unstained cells were used as autofluorescence control (AF). Cells without incubaction with primary antibody were used as control of secondary antibody (2nd Ab). Data were acquired by the BD LSR Fortessa system (Becton Dickinson) and analyzed by BDFACS Diva software.

### Induction of NETs

Neutrophils (1 × 10^5^ per well) were seeded in 96-well plates coated with 0.01 mg/ml poly-l-lysine (Sigma-Aldrich) and incubated for 30 min at 37 °C in a humidified 5% CO_2_ atmosphere to promote cell adhesion. NET formation was stimulated by exposure to proteases, including bovine pancreatic trypsin (Sigma-Aldrich), kallikrein 14 (KLK14; kindly provided by Dr. T. Kantyka), FX, FXa, FXa EGR (Thermo Fisher Scientific), cathepsin G (BioCentrum), neutrophil elastase (Athens Research & Technology) and/or RgpA. We also included the protease inhibitors aprotinin (Sigma-Aldrich) and/or Kyt-1 (Peptide Institute). The production of NETs was also triggered using the PAR2 agonists AC 264613 and/or SLIGRL-NH_2_ (Tocris Bioscience). As a negative control, we used the reversed amino acid sequence peptide LRGILS-NH_2_ (Tocris Bioscience). To block PAR2-dependent signaling, neutrophils were pre-treated with the peptide PAR2 antagonist FSLLRY-NH_2_ (Tocris Bioscience) before NETs were induced with proteases. All treatments lasted 3 h. To investigate ERK signaling during PAR2-activated NETs, neutrophils were pre-treated for 30 min with the selective ERK inhibitor UO126 (Cell Signaling Technology).

### DNA quantification

The total DNA content of NETs was determined by incubating neutrophils with 1 U/ml micrococcal nuclease (Thermo Fisher Scientific) for 15 min to release NETs from the cells. The free NETs were then separated from cells and debris by centrifugation (1800 × g, 10 min, room temperature) and the DNA was quantified using 10 μM SytoxGreen nucleic acid stain (Invitrogen) at a 1:10 (v/v) ratio. The fluorescence signal was obtained by excitation at 485 nm (emission wavelength 535 nM) and is presented as relative fluorescence units (RFU).

### Immunofluorescence staining

Slides coated with poly-l-lysine were seeded with 5 × 10^5^ human or mouse neutrophils and stimulated with PAR2 agonists and proteases for 3 h, as described above. The cells were then fixed for 10 min with 3.7% formaldehyde, blocked with PBS containing 5% fetal bovine serum (FBS), 1% bovine serum albumin (BSA), 0.05% Tween-20 and 2 mM EDTA for 1 h, and incubated with 0.1% saponin (Sigma-Aldrich) in PBS for 30 min. The cells were stained with rabbit anti-human neutrophil elastase antibodies (Athens Research and Technology, cat. no. 16-14-051200) for 1 h and goat anti-rabbit IgG F(ab′)_2_ antibodies conjugated to APC (Jackson ImmunoResearch Laboratories, cat. no. 111-136-144) for 45 min. The antibodies were suspended in PBS containing 3% BSA and 0.1% saponin. Nuclei were counterstained with Hoechst 33342 (1 μg/ml) for 10 min. All steps were carried out at room temperature with intervening washes (0.1% saponin in PBS). Confocal images were captured using a Zeiss LSM 880 microscope and ZEN software.

### Proteolytic enzymes

Arginine-specific gingipain (RgpA) was purified, followed by active-site titration as previously described [74].

### Treatment of mice prior to intravital microscopy

Experimental groups, with 3 mice in each group (6–8 weeks old), consisted of C57BL6/J wild-type and *PAR2*^–/–^ mice that received native mouse FXa (0.06 mg/kg; abcam) or PBS (FXa control group) via cannulated jugular vein 4 h before NET imaging. Wild-type mice were injected (i) *i.v*. with RgpA (0.8 mg/kg) 4 h before NET imaging, (ii) *i.v*. with RgpA and Kyt-1 (2.75 μg/kg) 4 h before NET imaging, (iii) *i.v*. with RgpA and also *i.p*. with apixaban (Medchem Express; 25 mg/kg) or DMSO (apixaban control group) 30 min prior to RgpA injection, or (iv) *i.p*. with 1 mg/kg LPS (*Escherichia coli* serotype 0111:B4; Sigma-Aldrich) in saline to induce endotoxemia [75] followed by intravital imaging 4 h after LPS injection.

### Preparation of mouse liver for intravital microscopy

Mice were anesthetized by *i.p*. injection with a mixture of ketamine hydrochloride (200 mg/kg; Biowet Pulawy) and xylazine hydrochloride (10 mg/kg; aniMedica) and were cannulated in the right jugular vein to facilitate the supply of anesthetics, antibodies and fluorescent dyes. Livers were prepared as previously described [76]. Briefly, the liver was exposed by a midline incision of the abdomen, followed by a lateral incision along the costal margin to the midaxillary line. The mouse was then placed in the right lateral position, and the ligaments attaching the liver to the stomach and diaphragm were cut, allowing the liver to be moved onto an imaging board covered with saline-soaked Kimwipes tissue. A cover glass was placed on the left liver lobe, and the space underneath the cover glass was filled with saline to keep the tissue moist. The mouse was then placed under the upright microscope for intravital imaging.

### Spinning-disk intravital microscopy (SD-IVM)

Livers were imaged using a Zeiss Axio Examiner. Z1 upright microscope equipped with an AMH-200-F6S metal halide light source (Andor, Oxford Instruments) with a motorized six-position excitation filter wheel and a DSD2 laser-free confocal spinning disk (Andor, Oxford Instruments) with Zeiss EC Plan-NEOFLUAR 10×/0.3 and Zeiss EC Plan-NEOFLUAR 20×/0.5 air objectives. The following excitation filters were used: DAPI, 390/40 nm; GFP, 482/18 nm; RFP, 561/14 nm; Cy5, 640/14 nm. These were paired with the corresponding emission filters: DAPI, 452/45 nm; GFP, 525/45 nm; RFP, 609/54 nm; Cy5, 676/29 nm. Images were captured using a Zyla 5.5 sCMOS camera (5.5 megapixels; Andor, Oxford Instruments) and iQ v3.6.1 acquisition software (Andor, Oxford Instruments).

### Visualization of neutrophils and NETs

NETs were visualized by the co-localization of neutrophil elastase, histone H2A.X and extracellular DNA. These were detected using an AlexaFluor 647-conjugated anti-neutrophil elastase monoclonal antibody (clone G-2; Santa Cruz Biotechnology; cat. no. sc-55549 AF647; 1.2 µg per mouse), an AlexaFluor 568-conjugated anti-H2A.X monoclonal antibody (clone 938CT5.1.1; Santa Cruz Biotechnology; cat. no. sc-517336; 0.8 µg per mouse) and 0.1 mM SytoxGreen in saline, respectively. All antibodies were injected *i.v*. via the cannulated jugular vein ~20 min before IVM. SytoxGreen stains DNA instantly, and was administrated during imaging. NETs were quantified as previously described [77] using ImageJ v1.53e (National Institutes of Health) and expressed as the percentage of liver area covered in each field of view, with at least five fields analyzed per mouse.

### Bactericidal activity of NETs

Neutrophils seeded in 24-well plates (2 × 10^6^/well) coated with 0.01 mg/ml poly-l-lysine were centrifuged (200 × g, 5 min, room temperature) before we added 100 µM AC 264613 and 1 µM FXa. We used 0.025 µM phorbol 12-myristate 13-acetate (PMA) to induce classical NETs. After 3 h, NETs were collected and incubated with 4 × 10^6^ *E. coli* ATCC 29522 or *E. coli* DH5α cells. As a control, the same bacterial strains were incubated in supernatant from untreated neutrophils. After 2 h, bacterial survival was estimated by plating serial dilutions on solid agar plates and counting the colony forming units (CFUs).

### Enzymatic assays

NETs generated in response to PMA (0.025 µM), AC 264613 (100 µM), trypsin (0.05 µM) and/or FXa (1 µM) were collected, and the activities of neutrophil serine proteases were measured using specific substrates. Neutrophil elastase and cathepsin G activities were determined using *N*-methoxysuccinyl-Ala-Ala-Pro-Val-*p*-nitroanilide (Sigma-Aldrich) and *N*-succinyl-Ala-Ala-Pro-Phe-*p*-nitroanilide (Sigma-Aldrich), respectively. Each substrate (1 mM in 100 μl 50 mM Tris-HCl, pH 7.5) was mixed with 100 μl of supernatant from the netting and control neutrophils, and the rate of substrate hydrolysis was measured as the increase in the optical density at 450 nm (OD_450_) after incubation for 30 min at 37 °C.

### SDS-PAGE and immunoblotting

NETs and whole protein lysates were obtained from neutrophils by using Pierce RIPA Buffer (Thermo Scientific) with proteinase and/or phosphatase inhibitors and equal amounts of protein were separated by SDS-PAGE. The proteins from NETs and lysates for AKT were transferred to PVDF membranes (Merck Millipore) in 25 mM Tris-HCl, 0.2 M glycine (pH 8.3) supplemented with 20% methanol (60 V, 3 h, 4 °C) or to nitrocellulose membrane for ERK. Non-specific binding sites were blocked with 5% skimmed milk for PVDF or 5% BSA for nitrocellulose membrane in Tris-buffered saline (pH 7.5) containing Tween-20 (TTBS) for 1 h at room temperature, followed by overnight incubation at 4 °C with antibodies. Mouse LL-37/CAP-18 (1:500, Hycult Biotech, cat. no. mAb 3D11), PathScan® Multiplex Western Cocktail I (rabbit P-ERK1/2, Thr202/Tyr204 (1:1000, Cell Signaling, cat. no. 5301), rabbit ERK1/2 (1:500, Cell Signaling, cat. no. 9102), rabbit Rab 11 (1:1000, Cell Signaling, cat. no. 3539)), rabbit P-AKT (T308) (1:1000, Cell Signalling, cat. no. 13038), rabbit AKT (1:1000, Cell Signalling, cat. no. 9272), rabbit GAPDH (1:5000, Cell Signaling, cat. no. 2118) were used in 5% skimmed milk or 3% BSA in TTBS, respectively for PVDF and nitrocellulose membrane. Membranes were washed extensively in TTBS and incubated with a 1:20000 dilution of a horseradish peroxidase (HRP)-conjugated sheep anti-mouse IgG secondary antibody (Sigma-Aldrich, cat. no. AC111P) and with goat anti-rabbit IgG (1:5000; Cell Signaling, cat. no. 7074) for 1 h in TTBS containing 5% skimmed milk or 3% BSA in TTBS. Membranes were washed (5 × 5 min) in TTBS, and blots were developed using enhanced chemiluminescence (ECL) substrate (Thermo Fisher Scientific).

### Intracellular calcium measurement

Neutrophils were seeded at a density of 0.5 × 10^6^ cells/well in black 96-well plates with clear bottoms (coated with 0.01 mg/ml poly-l-lysine) and were incubated with 80 μl 1× calcium dye 5 in 1× Hanks’ balanced salt solution (HBSS) supplemented with 20 mM *N*-2-hydroxyethylpiperazine-*N*′-2-ethanesulfonic acid (HEPES) in the presence of 2 mM probenecid (Molecular Devices). PAR2 activators (40 μl/well) were added using the Flex Station 3 multimode microplate reader (Molecular Devices), and fluorescence readings were acquired for 120 s at an excitation wavelength of 485 nm (emission wavelength 525 nm). The final concentration of the compounds was 100 μM AC 264613, 0.25 μM trypsin, and 1 μM FXa. Background fluorescence was recorded in untreated cells in the same assay buffer.

### Mass spectrometry (LC-MS/MS) analysis of NETs proteome

Neutrophils were subjected to AC 264613 or untreated (control) for 3 hours, then treated with MNase (1 U/ml) for 15 minutes and centrifuged at 1800 × g for 10 minutes. Ice-cold acetone was added to the supernatants containing NETs, then samples were incubated at −80 °C for 60 minutes and centrifuged at 15,000 × g for 10 minutes. The supernatant obtained by centrifugation was withdrawn, the protein pellet was dried and dissolved in PBS. Then, samples were lyophilized in a speed vacuum concentrator before resuspension in 8 M urea, 100 mM ammonium bicarbonate. Proteins were reduced with 10 mM dithiothretiol for 30 min, followed by alkylation with 30 mM iodoacetamide for 30 min, in the dark. The urea concentration was lowered by the addition of 100 mM ammonium bicarbonate and then digested with trypsin at a 1:50 (w/w) ratio overnight at 37 °C. Digested peptides were acidified with formic acid and desalted on homemade reverse-phase C18 columns packed with Octadecyl C18 Solid Phase Extraction disks (Empore, 3 M). The samples were eluted from the columns with 70% acetonitrile in 0.1% formic acid, lyophilized to near-dryness and redissolved in 0.1% formic acid. The samples were analyzed on an Orbitrap Eclipse Tribrid mass spectrometer (Thermo Fisher Scientific) coupled to an EASY-nLC 1200 (Thermo Fisher Scientific). 750 ng of each sample was loaded onto a trap column (2 cm × 75 µm inner diameter) and separated on an analytical column (20 cm × 75 µm inner diameter) packed with 1.9 µm C18 beads (Dr. Maisch, GmbH). The samples were eluted at a flow rate of 250 nl/min and with a gradient from 5 to 35% acetonitrile for 80 min, followed by a steep increase to 80% acetonitrile for 10 min. The MS raw files were processed in Proteome Discoverer 2.5 using the Sequest HT search algorithm. The UniProt human reference proteome was used as a database with the following parameters: 10 ppm precursor mass accuracy, 0.02 Da fragment mass accuracy, trypsin as digestion enzyme, max. 2 missed cleavage sites, Oxidation (M) as variable modification and Acetyl (N-Term), Met-loss (M), and Met-loss+Acetyl (M) as variable protein N-terminus modifications. Carbamidomethyl (C) as fixed modification. Protein abundances were label-free quantified based on precursor area and using only unique peptides. The samples were normalized to the total peptide amount. Further data analysis was conducted in Perseus (www.perseus-framework.org) and MATLAB (R2022b) by MathWorks. Data in Venn diagram were filtered to proteins identified with a 1% FDR and minimum two unique peptides. Data in Volcano plot were filtered to include proteins identified with a 1% FDR with a minimum of two unique peptides and quantified in all three replicates in at least one sample group. Protein abundances were log2 transformed before further processing. Missing values were drawn at random from a normal distribution downshifted by 1.8 and with a width of 0.3 compared to the experimental data. *P*-values were calculated with an unpaired equal variance t-test and FDR adjusted using the Benjamini-Hochberg procedure. Proteins were considered differential abundant at a fold change ≥2 and a BH adj. *P*-value < 0.05.

### Bioinformatics Analysis

Principal component analysis (PCA) was performed using Python 3.12 (Python Software Foundation - PSF). The components were selected by parallel analysis. The graph was made using the Prism software 9 (GraphPad, La Jolla, CA, USA). Venn diagrams was created using the InteractiVenn platform [78] and modified in Inkscape (Inkscape Team). Bubble plots and Volcano plot were made using SRplot [79]. GO Enrichment, KEGG Pathways and Reactome analysis was performed using the DAVID Gene Functional Classification Tool database. Data were considered statistically significant if *p* value was *p* ≤ 0.05.

### Turbidity mesurements

Turbidity measurements were performed to analyze the kinetics of clot formation in FX-deficient plasma (HYPHEN BioMed) under the influence of FXa-derived NETs and/or purified FXa. After 3 hours, 25 µl of collected NETs and 2 µg of prothrombin were resuspended in 25 µl of 10 mM HEPES pH 7.4, 150 mM NaCl and 25 mM CaCl_2_. Then, 50 µl of FX -deficient plasma initiated clotting what was monitored by measuring absorbance at a wavelength of 400 nm for 2 h at 37 °C and presented as Vmax [RFU/min]. As a control of clot formation FXa was used.

### Scanning electron microscopy (SEM)

Clots formed under the influence of FXa-derived NETs and/or FXa were fixed in 2.5% glutaraldehyde in 0.1 M sodium cacodylate buffer (pH 7.4). After fixation, the sections were washed in sodium cacodylate buffer and post-fixed in 1% osmium tetroxide. Next, samples were dehydrated in an alcohol series, dried, and sputtered with gold. Images were captured with a JSM5410 scanning electron microscope (JEOL) at the Institute of Zoology, Jagiellonian University, in Krakow, Poland.

### Statistics

Statistical significance was determined using GraphPad Prism v7 (GraphPad Software) by applying a two-tailed unpaired t-test or by one-way analysis of variance (ANOVA) with Tukey’s multiple comparisons test. All values are expressed as means ± standard errors (SEM) and *P* values < 0.05 were considered statistically significant.

## Supplementary information

Supplementary Information

Original Data

original western blot file

original western blot file

original western blot file

original western blot file

original western blot file

## Data Availability

All data needed to evaluate the conclusions in the paper are present in the paper and/or the Supplementary Information. The raw data supporting the findings of this study are available from the corresponding author upon reasonable request. The mass spectrometry proteomics data have been deposited to the ProteomeXchange Consortium via the PRIDE [80] partner repository with the dataset identifier PXD055633.
