# Supplementary material for: Non-classical neutrophil extracellular traps induced by PAR2-signaling proteases
Source: Cell Death Dis. 2025 Feb 19;16(1):109. doi: 10.1038/s41419-025-07428-z (PMC11840154; doi:10.1038/s41419-025-07428-z)
Supplement: Supplementary file 1 — Supplementary Information [file 41419_2025_7428_MOESM1_ESM.pdf]

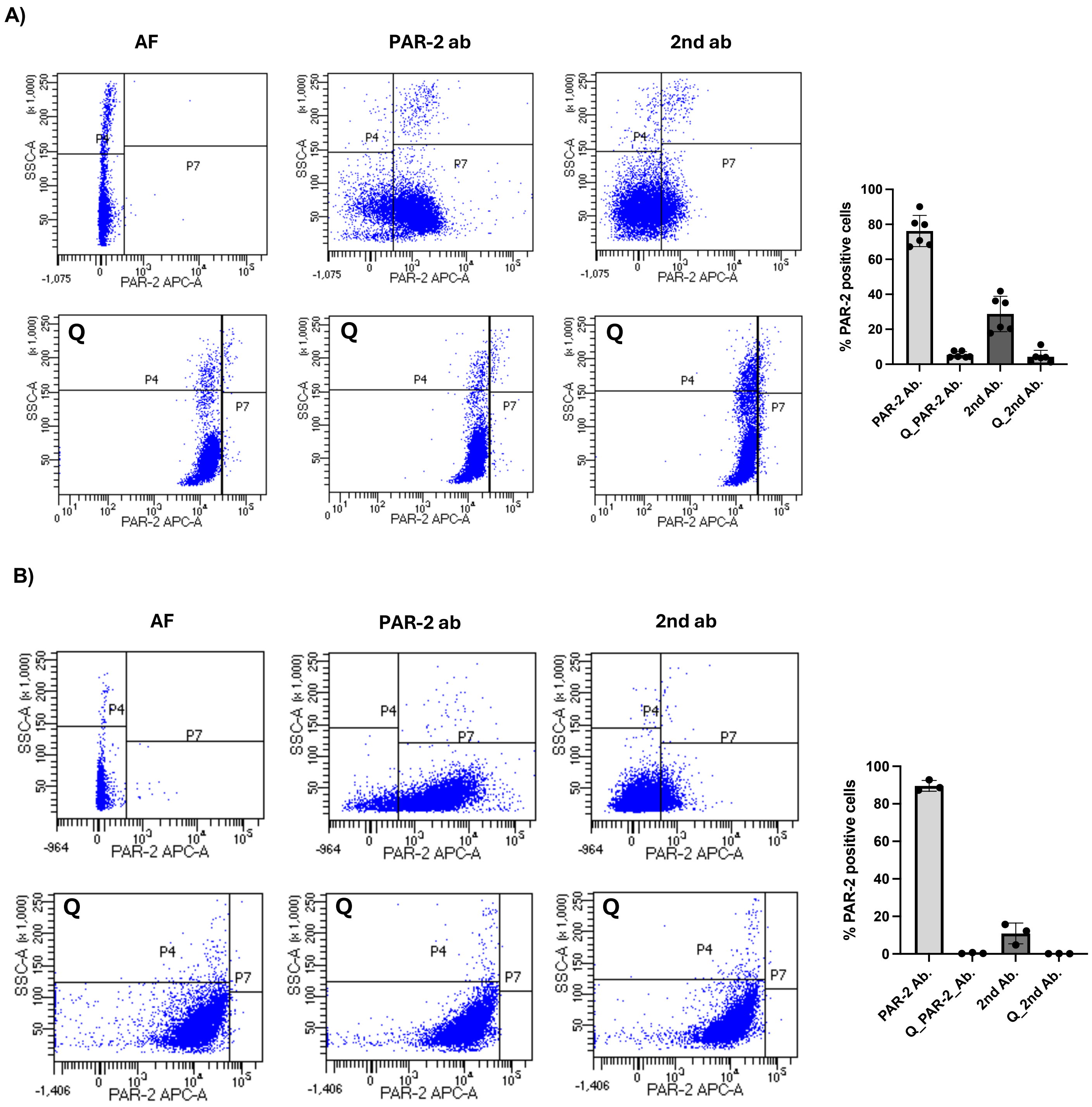

Fig. S1. Identification of PAR-2 protein on the surface of neutrophils. The presence of PAR-2 receptor on the surface of (A) human and (B) murine neutrophils was detected with anti-PAR2 antibody and goat anti rabbit IgG conjugated with APC. After fixation and permeabilization cells were stained with PAR-2 specific antibodies. Additionally, trypan blue quenching (Q) was performed to confirm the localization of the receptor. Results were obtained from PMN isolated from 3 donors in duplicates (A) and neutrophils isolated from bone marrow of 3 mice (B) shown as mean ± SEM.

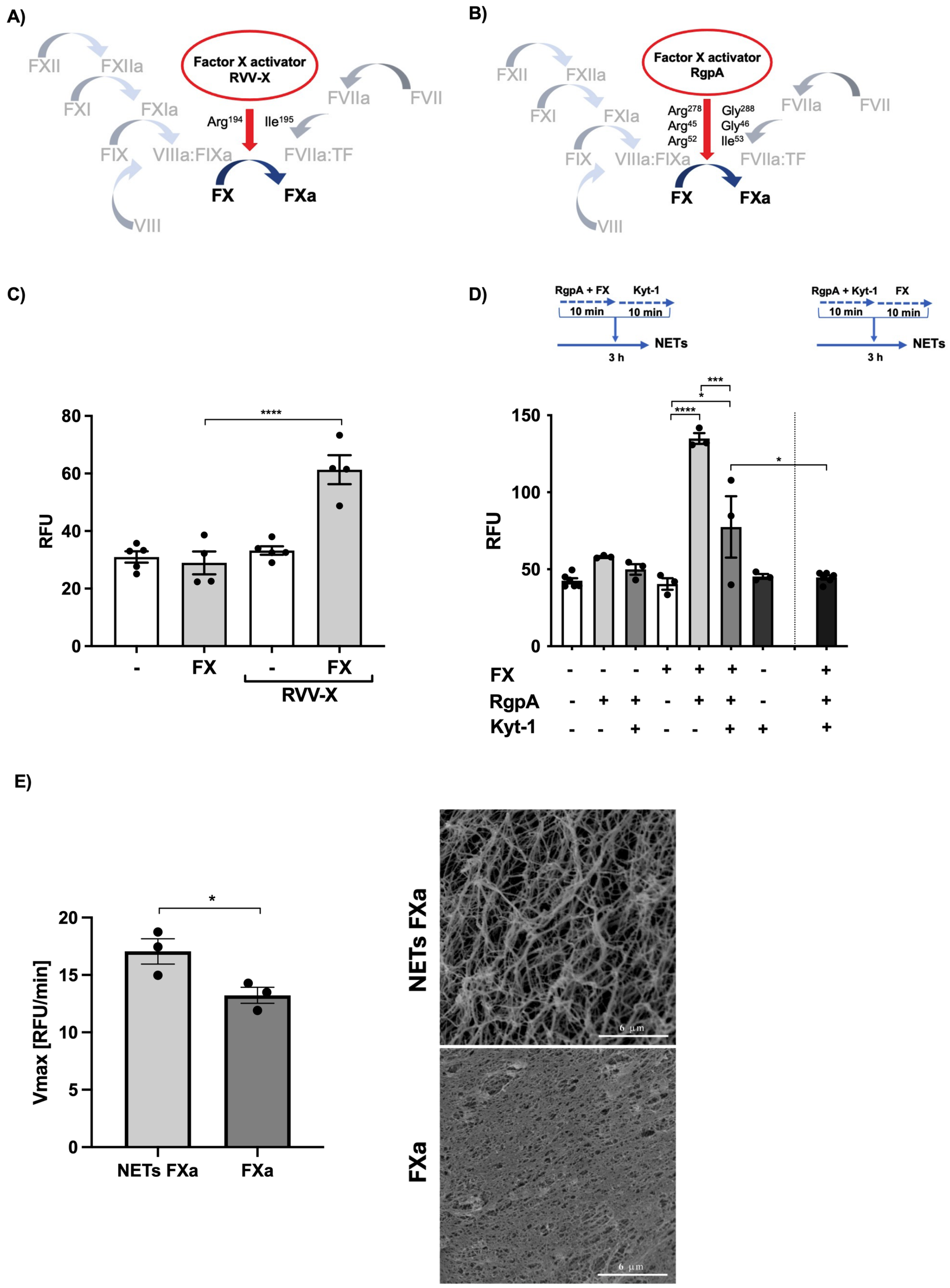

Fig. S2. Generation of FXa by Russell viper venom (RVV-X) and gingipain RgpA leads to the formation of NETs *in vitro*. (A, B) The cleavage sites for RVV-X (A) and RgpA (B) in FX, leading to the formation of FXa. (C) Induction of NETs by FX (1 µM) modified using RVV-X (1 µg/ml). After incubation for 3 h, NET formation was determined by SytoxGreen staining. (D) The amount of extracellular DNA, measured by SytoxGreen staining, released by neutrophils 3 h post-incubation with 10 nM RgpA and/or 1 µM FX in the presence or absence of Kyt-1 at a final concentration of 1 µM. (E) Kinetic measurement of clot formation induced by FXa- derived NETs and/or FXa, and their visualization by SEM. (C, D) Statistical significance was evaluated by one-way ANOVA followed by Tukey's multiple comparisons test, or (E) by applying an unpaired *t*-test. Data are means (± SEM) of at least n = 3 separate experiments (*P < 0.05, ***P < 0.001, ****P < 0.0001).

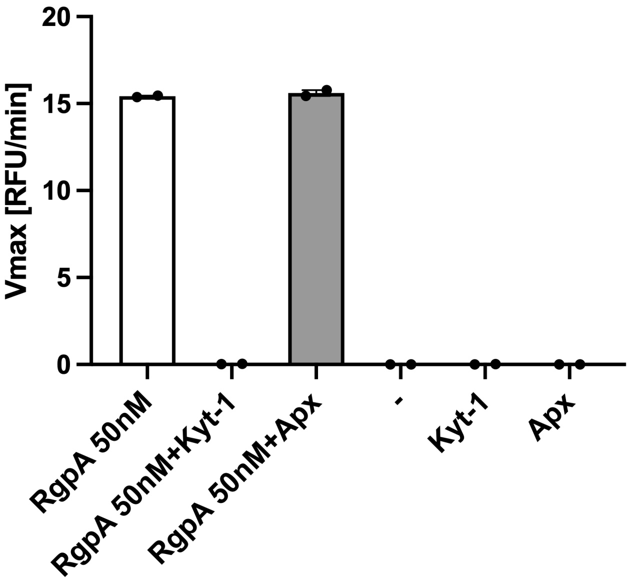

Fig. S3. Apixaban has no effect on the activity of gingipain RgpA. The efficiency of RgpA activity after apixaban (10 μM) treatment was verified using L - Ba*p*NA as a substrate for Arg-X gingipains in TNC buffer with 0.05 % Tween-20. Substrate hydrolysis was recorded at 405 nm for 40 min, and activity was expressed as V max [RFU/min] by a Flex Station 3 multimode microplate reader. For control, gingipain-specific inhibitor Kyt-1 (1 μM) was used.

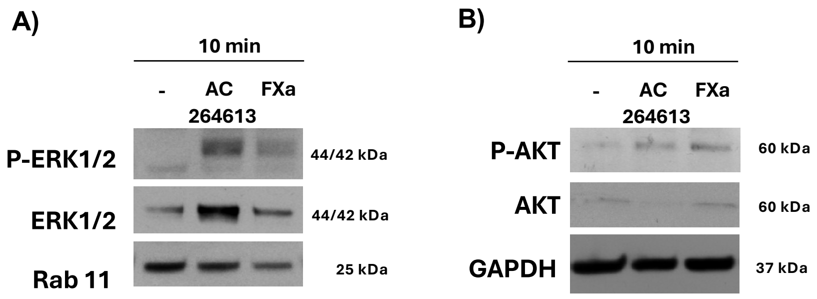

Fig. S4. Coagulation factor FXa activates MEK/ERK and PI3K-AKT pathways. Representative Western Blot analysis of phosphorylation of ERK1/2 Thr202/Tyr204 (A) and AKT T308 (B) after 10 min of PMN stimulation with AC264613 (100 μM) and/or FXa (500 nM). Rab 11 was used as a reference gene for ERK (A), and GAPDH for AKT (B).

Nr	Donor 1	Donor 2	Donor 3	Donor 1	Donor 2	Donor 3
	control	conrol	control	AC 264613	AC 264613	AC 264613
1	1-phosphatidylinositol 4,5-bisphosphate phosphodiesterase gamma-2		1-phosphatidylinositol 4,5-bisphosphate phosphodiesterase gamma-2	1-phosphatidylinositol 4,5-bisphosphate phosphodiesterase gamma-2	1-phosphatidylinositol 4,5-bisphosphate phosphodiesterase gamma-2	1-phosphatidylinositol 4,5-bisphosphate phosphodiesterase gamma-2
2	1,4-alpha-glucan-branching enzyme	1,4-alpha-glucan-branching enzyme	1,4-alpha-glucan-branching enzyme	1,4-alpha-glucan-branching enzyme	1,4-alpha-glucan-branching enzyme	1,4-alpha-glucan-branching enzyme
3	10 kDa heat shock protein, mitochondrial	10 kDa heat shock protein, mitochondrial	10 kDa heat shock protein, mitochondrial	10 kDa heat shock protein, mitochondrial	10 kDa heat shock protein, mitochondrial	10 kDa heat shock protein, mitochondrial
4	14-3-3 protein beta/alpha	14-3-3 protein beta/alpha	14-3-3 protein beta/alpha	14-3-3 protein beta/alpha	14-3-3 protein beta/alpha	14-3-3 protein beta/alpha
5	14-3-3 protein epsilon	14-3-3 protein epsilon	14-3-3 protein epsilon	14-3-3 protein epsilon	14-3-3 protein epsilon	14-3-3 protein epsilon
6	14-3-3 protein eta	14-3-3 protein eta	14-3-3 protein eta	14-3-3 protein eta	14-3-3 protein eta	14-3-3 protein eta
7	14-3-3 protein gamma	14-3-3 protein gamma	14-3-3 protein gamma	14-3-3 protein gamma	14-3-3 protein gamma	14-3-3 protein gamma
8		14-3-3 protein theta	14-3-3 protein theta	14-3-3 protein theta	14-3-3 protein theta	14-3-3 protein theta
9	14-3-3 protein zeta/delta	14-3-3 protein zeta/delta	14-3-3 protein zeta/delta	14-3-3 protein zeta/delta	14-3-3 protein zeta/delta	14-3-3 protein zeta/delta
10	26S proteasome non-ATPase regulatory subunit 10				26S proteasome non-ATPase regulatory subunit 10	26S proteasome non-ATPase regulatory subunit 10
11		26S proteasome non-ATPase regulatory subunit 3	26S proteasome non-ATPase regulatory subunit 3	26S proteasome non-ATPase regulatory subunit 3	26S proteasome non-ATPase regulatory subunit 3	26S proteasome non-ATPase regulatory subunit 3
12	26S proteasome non-ATPase regulatory subunit 9	26S proteasome non-ATPase regulatory subunit 9	26S proteasome non-ATPase regulatory subunit 9	26S proteasome non-ATPase regulatory subunit 9	26S proteasome non-ATPase regulatory subunit 9	26S proteasome non-ATPase regulatory subunit 9
13	26S proteasome regulatory subunit 7	26S proteasome regulatory subunit 7	26S proteasome regulatory subunit 7	26S proteasome regulatory subunit 7	26S proteasome regulatory subunit 7	26S proteasome regulatory subunit 7
14				5'-AMP-activated protein kinase catalytic subunit alpha-1		5'-AMP-activated protein kinase catalytic subunit alpha-1
15	6-phosphogluconate dehydrogenase, decarboxylating	6-phosphogluconate dehydrogenase, decarboxylating	6-phosphogluconate dehydrogenase, decarboxylating	6-phosphogluconate dehydrogenase, decarboxylating	6-phosphogluconate dehydrogenase, decarboxylating	6-phosphogluconate dehydrogenase, decarboxylating
16		6-phosphogluconolactonase	6-phosphogluconolactonase	6-phosphogluconolactonase	6-phosphogluconolactonase	6-phosphogluconolactonase
17	Acid ceramidase	Acid ceramidase	Acid ceramidase	Acid ceramidase	Acid ceramidase	Acid ceramidase
18	Acidic leucine-rich nuclear phosphoprotein 32 family member A	Acidic leucine-rich nuclear phosphoprotein 32 family member A	Acidic leucine-rich nuclear phosphoprotein 32 family member A	Acidic leucine-rich nuclear phosphoprotein 32 family member A	Acidic leucine-rich nuclear phosphoprotein 32 family member A	Acidic leucine-rich nuclear phosphoprotein 32 family member A
19	Actin nucleation-promoting factor WAS	Actin nucleation-promoting factor WAS	Actin nucleation-promoting factor WAS	Actin nucleation-promoting factor WAS	Actin nucleation-promoting factor WAS	Actin nucleation-promoting factor WAS
20	Actin-related protein 2	Actin-related protein 2	Actin-related protein 2	Actin-related protein 2	Actin-related protein 2	Actin-related protein 2
21	Actin-related protein 2/3 complex subunit 1B	Actin-related protein 2/3 complex subunit 1B	Actin-related protein 2/3 complex subunit 1B	Actin-related protein 2/3 complex subunit 1B	Actin-related protein 2/3 complex subunit 1B	Actin-related protein 2/3 complex subunit 1B
22	Actin-related protein 2/3 complex subunit 2	Actin-related protein 2/3 complex subunit 2	Actin-related protein 2/3 complex subunit 2	Actin-related protein 2/3 complex subunit 2	Actin-related protein 2/3 complex subunit 2	Actin-related protein 2/3 complex subunit 2
23	Actin-related protein 2/3 complex subunit 3	Actin-related protein 2/3 complex subunit 3	Actin-related protein 2/3 complex subunit 3	Actin-related protein 2/3 complex subunit 3	Actin-related protein 2/3 complex subunit 3	Actin-related protein 2/3 complex subunit 3
24	Actin-related protein 2/3 complex subunit 4	Actin-related protein 2/3 complex subunit 4	Actin-related protein 2/3 complex subunit 4	Actin-related protein 2/3 complex subunit 4	Actin-related protein 2/3 complex subunit 4	Actin-related protein 2/3 complex subunit 4
25	Actin-related protein 2/3 complex subunit 5	Actin-related protein 2/3 complex subunit 5	Actin-related protein 2/3 complex subunit 5	Actin-related protein 2/3 complex subunit 5	Actin-related protein 2/3 complex subunit 5	Actin-related protein 2/3 complex subunit 5
26	Actin-related protein 3	Actin-related protein 3	Actin-related protein 3	Actin-related protein 3	Actin-related protein 3	Actin-related protein 3
27	Actin, alpha skeletal muscle	Actin, alpha skeletal muscle	Actin, alpha skeletal muscle	Actin, alpha skeletal muscle	Actin, alpha skeletal muscle	Actin, alpha skeletal muscle
28	Actin, cytoplasmic 1	Actin, cytoplasmic 1	Actin, cytoplasmic 1	Actin, cytoplasmic 1	Actin, cytoplasmic 1	Actin, cytoplasmic 1
29	Actin, cytoplasmic 2	Actin, cytoplasmic 2	Actin, cytoplasmic 2	Actin, cytoplasmic 2	Actin, cytoplasmic 2	Actin, cytoplasmic 2
30	Activated RNA polymerase II transcriptional coactivator p15	Activated RNA polymerase II transcriptional coactivator p15		Activated RNA polymerase II transcriptional coactivator p15	Activated RNA polymerase II transcriptional coactivator p15	Activated RNA polymerase II transcriptional coactivator p15

31	Acyl-CoA-binding protein	Acyl-CoA-binding protein		Acyl-CoA-binding protein	Acyl-CoA-binding protein	Acyl-CoA-binding protein
32	Acylamino-acid-releasing enzyme	Acylamino-acid-releasing enzyme	Acylamino-acid-releasing enzyme	Acylamino-acid-releasing enzyme	Acylamino-acid-releasing enzyme	Acylamino-acid-releasing enzyme
33	Adapter SH3BGRL	Adapter SH3BGRL	Adapter SH3BGRL	Adapter SH3BGRL	Adapter SH3BGRL	Adapter SH3BGRL
34		Adenine phosphoribosyltransferase		Adenine phosphoribosyltransferase	Adenine phosphoribosyltransferase	Adenine phosphoribosyltransferase
35	Adenosine 5'-monophosphoramidase HINT1	Adenosine 5'-monophosphoramidase HINT1	Adenosine 5'-monophosphoramidase HINT1	Adenosine 5'-monophosphoramidase HINT1	Adenosine 5'-monophosphoramidase HINT1	Adenosine 5'-monophosphoramidase HINT1
36	Adenosine kinase	Adenosine kinase	Adenosine kinase	Adenosine kinase	Adenosine kinase	Adenosine kinase
37	Adenosylhomocysteinase	Adenosylhomocysteinase	Adenosylhomocysteinase	Adenosylhomocysteinase	Adenosylhomocysteinase	Adenosylhomocysteinase
38	Adenylate kinase 2, mitochondrial	Adenylate kinase 2, mitochondrial	Adenylate kinase 2, mitochondrial	Adenylate kinase 2, mitochondrial	Adenylate kinase 2, mitochondrial	Adenylate kinase 2, mitochondrial
39	Adenylate kinase isoenzyme 1	Adenylate kinase isoenzyme 1	Adenylate kinase isoenzyme 1	Adenylate kinase isoenzyme 1	Adenylate kinase isoenzyme 1	Adenylate kinase isoenzyme 1
40	Adenylosuccinate synthetase isozyme 2	Adenylosuccinate synthetase isozyme 2	Adenylosuccinate synthetase isozyme 2	Adenylosuccinate synthetase isozyme 2	Adenylosuccinate synthetase isozyme 2	Adenylosuccinate synthetase isozyme 2
41	Adenylyl cyclase-associated protein 1	Adenylyl cyclase-associated protein 1	Adenylyl cyclase-associated protein 1	Adenylyl cyclase-associated protein 1	Adenylyl cyclase-associated protein 1	Adenylyl cyclase-associated protein 1
42	ADP-ribosyl cyclase/cyclic ADP-ribose hydrolase 2	ADP-ribosyl cyclase/cyclic ADP-ribose hydrolase 2	ADP-ribosyl cyclase/cyclic ADP-ribose hydrolase 2	ADP-ribosyl cyclase/cyclic ADP-ribose hydrolase 2	ADP-ribosyl cyclase/cyclic ADP-ribose hydrolase 2	ADP-ribosyl cyclase/cyclic ADP-ribose hydrolase 2
43	ADP-ribosylation factor 3	ADP-ribosylation factor 3	ADP-ribosylation factor 3	ADP-ribosylation factor 3	ADP-ribosylation factor 3	ADP-ribosylation factor 3
44				ADP-ribosylation factor-like protein 8B	ADP-ribosylation factor-like protein 8B	ADP-ribosylation factor-like protein 8B
45		ADP-sugar pyrophosphatase	ADP-sugar pyrophosphatase	ADP-sugar pyrophosphatase	ADP-sugar pyrophosphatase	ADP-sugar pyrophosphatase
46	Albumin	Albumin	Albumin	Albumin	Albumin	Albumin
47		Alcohol dehydrogenase class-3		Alcohol dehydrogenase class-3	Alcohol dehydrogenase class-3	Alcohol dehydrogenase class-3
48	Aldo-keto reductase family 1 member A1	Aldo-keto reductase family 1 member A1	Aldo-keto reductase family 1 member A1	Aldo-keto reductase family 1 member A1	Aldo-keto reductase family 1 member A1	Aldo-keto reductase family 1 member A1
49	Allograft inflammatory factor 1	Allograft inflammatory factor 1	Allograft inflammatory factor 1	Allograft inflammatory factor 1	Allograft inflammatory factor 1	Allograft inflammatory factor 1
50	Alpha-1-acid glycoprotein 1	Alpha-1-acid glycoprotein 1	Alpha-1-acid glycoprotein 1	Alpha-1-acid glycoprotein 1	Alpha-1-acid glycoprotein 1	Alpha-1-acid glycoprotein 1
51	Alpha-1-acid glycoprotein 2	Alpha-1-acid glycoprotein 2	Alpha-1-acid glycoprotein 2	Alpha-1-acid glycoprotein 2	Alpha-1-acid glycoprotein 2	Alpha-1-acid glycoprotein 2
52	Alpha-1-antitrypsin	Alpha-1-antitrypsin	Alpha-1-antitrypsin	Alpha-1-antitrypsin	Alpha-1-antitrypsin	
53	Alpha-1B-glycoprotein	Alpha-1B-glycoprotein	Alpha-1B-glycoprotein	Alpha-1B-glycoprotein	Alpha-1B-glycoprotein	Alpha-1B-glycoprotein
54			Alpha-2-HS-glycoprotein	Alpha-2-HS-glycoprotein	Alpha-2-HS-glycoprotein	Alpha-2-HS-glycoprotein
55	Alpha-actinin-1	Alpha-actinin-1	Alpha-actinin-1	Alpha-actinin-1	Alpha-actinin-1	Alpha-actinin-1
56	Alpha-actinin-4	Alpha-actinin-4	Alpha-actinin-4	Alpha-actinin-4	Alpha-actinin-4	Alpha-actinin-4
57		Alpha-adducin	Alpha-adducin	Alpha-adducin	Alpha-adducin	Alpha-adducin
58	Alpha-centractin	Alpha-centractin	Alpha-centractin		Alpha-centractin	Alpha-centractin
59	Alpha-endosulfine	Alpha-endosulfine	Alpha-endosulfine	Alpha-endosulfine	Alpha-endosulfine	Alpha-endosulfine
60	Alpha-enolase	Alpha-enolase	Alpha-enolase	Alpha-enolase	Alpha-enolase	Alpha-enolase
61		Amiloride-sensitive amine oxidase [copper-containing]		Amiloride-sensitive amine oxidase [copper-containing]	Amiloride-sensitive amine oxidase [copper-containing]	
62	Aminoacyl tRNA synthase complex-interacting multifunctional protein 1	Aminoacyl tRNA synthase complex-interacting multifunctional protein 1	Aminoacyl tRNA synthase complex-interacting multifunctional protein 1	Aminoacyl tRNA synthase complex-interacting multifunctional protein 1	Aminoacyl tRNA synthase complex-interacting multifunctional protein 1	Aminoacyl tRNA synthase complex-interacting multifunctional protein 1

63		Aminopeptidase B	Aminopeptidase B	Aminopeptidase B	Aminopeptidase B	Aminopeptidase B
64	Aminopeptidase N	Aminopeptidase N	Aminopeptidase N	Aminopeptidase N	Aminopeptidase N	Aminopeptidase N
65				AMP deaminase 3	AMP deaminase 3	AMP deaminase 3
66	Amyloid-beta precursor protein	Amyloid-beta precursor protein	Amyloid-beta precursor protein			Amyloid-beta precursor protein
67		Ankyrin-1			Ankyrin-1	Ankyrin-1
68	Annexin A1	Annexin A1	Annexin A1	Annexin A1	Annexin A1	Annexin A1
69	Annexin A11	Annexin A11	Annexin A11	Annexin A11	Annexin A11	Annexin A11
70	Annexin A3	Annexin A3	Annexin A3	Annexin A3	Annexin A3	Annexin A3
71	Annexin A4	Annexin A4	Annexin A4	Annexin A4	Annexin A4	Annexin A4
72	Annexin A5	Annexin A5	Annexin A5	Annexin A5	Annexin A5	Annexin A5
73	Annexin A6	Annexin A6	Annexin A6	Annexin A6	Annexin A6	Annexin A6
74	Antithrombin-III	Antithrombin-III	Antithrombin-III	Antithrombin-III	Antithrombin-III	Antithrombin-III
75				AP-1 complex subunit beta-1	AP-1 complex subunit beta-1	AP-1 complex subunit beta-1
76	Apolipoprotein A-I	Apolipoprotein A-I	Apolipoprotein A-I	Apolipoprotein A-I	Apolipoprotein A-I	Apolipoprotein A-I
77	Apolipoprotein A-II	Apolipoprotein A-II	Apolipoprotein A-II		Apolipoprotein A-II	Apolipoprotein A-II
78	Apolipoprotein B receptor	Apolipoprotein B receptor	Apolipoprotein B receptor	Apolipoprotein B receptor	Apolipoprotein B receptor	Apolipoprotein B receptor
79	Apoptosis regulator BAX	Apoptosis regulator BAX	Apoptosis regulator BAX	Apoptosis regulator BAX	Apoptosis regulator BAX	Apoptosis regulator BAX
80		Apoptosis-associated speck-like protein containing a CARD	Apoptosis-associated speck-like protein containing a CARD	Apoptosis-associated speck-like protein containing a CARD	Apoptosis-associated speck-like protein containing a CARD	Apoptosis-associated speck-like protein containing a CARD
81	Arf-GAP with coiled-coil, ANK repeat and PH domain-containing protein 1	Arf-GAP with coiled-coil, ANK repeat and PH domain-containing protein 1	Arf-GAP with coiled-coil, ANK repeat and PH domain-containing protein 1	Arf-GAP with coiled-coil, ANK repeat and PH domain-containing protein 1	Arf-GAP with coiled-coil, ANK repeat and PH domain-containing protein 1	Arf-GAP with coiled-coil, ANK repeat and PH domain-containing protein 1
82	Arf-GAP with coiled-coil, ANK repeat and PH domain-containing protein 2	Arf-GAP with coiled-coil, ANK repeat and PH domain-containing protein 2	Arf-GAP with coiled-coil, ANK repeat and PH domain-containing protein 2	Arf-GAP with coiled-coil, ANK repeat and PH domain-containing protein 2		Arf-GAP with coiled-coil, ANK repeat and PH domain-containing protein 2
83	Arf-GAP with Rho-GAP domain, ANK repeat and PH domain-containing protein 1	Arf-GAP with Rho-GAP domain, ANK repeat and PH domain-containing protein 1	Arf-GAP with Rho-GAP domain, ANK repeat and PH domain-containing protein 1	Arf-GAP with Rho-GAP domain, ANK repeat and PH domain-containing protein 1	Arf-GAP with Rho-GAP domain, ANK repeat and PH domain-containing protein 1	Arf-GAP with Rho-GAP domain, ANK repeat and PH domain-containing protein 1
84	Arginase-1	Arginase-1	Arginase-1	Arginase-1	Arginase-1	Arginase-1
85		Arylsulfatase A	Arylsulfatase A		Arylsulfatase A	Arylsulfatase A
86	Arylsulfatase B	Arylsulfatase B	Arylsulfatase B	Arylsulfatase B	Arylsulfatase B	Arylsulfatase B
87	Aspartate aminotransferase, cytoplasmic	Aspartate aminotransferase, cytoplasmic	Aspartate aminotransferase, cytoplasmic	Aspartate aminotransferase, cytoplasmic	Aspartate aminotransferase, cytoplasmic	Aspartate aminotransferase, cytoplasmic
88	ATP synthase subunit beta, mitochondrial	ATP synthase subunit beta, mitochondrial	ATP synthase subunit beta, mitochondrial	ATP synthase subunit beta, mitochondrial	ATP synthase subunit beta, mitochondrial	ATP synthase subunit beta, mitochondrial
89	ATP-citrate synthase	ATP-citrate synthase	ATP-citrate synthase	ATP-citrate synthase	ATP-citrate synthase	ATP-citrate synthase
90		ATP-dependent 6-phosphofructokinase, liver type		ATP-dependent 6-phosphofructokinase, liver type	ATP-dependent 6-phosphofructokinase, liver type	ATP-dependent 6-phosphofructokinase, liver type
91		ATPase inhibitor, mitochondrial	ATPase inhibitor, mitochondrial		ATPase inhibitor, mitochondrial	
92	Azurocidin	Azurocidin	Azurocidin	Azurocidin	Azurocidin	Azurocidin
93		B-cell receptor-associated protein 31	B-cell receptor-associated protein 31	B-cell receptor-associated protein 31	B-cell receptor-associated protein 31	B-cell receptor-associated protein 31
94	Bactericidal permeability-increasing protein	Bactericidal permeability-increasing protein	Bactericidal permeability-increasing protein	Bactericidal permeability-increasing protein	Bactericidal permeability-increasing protein	Bactericidal permeability-increasing protein

95	Band 3 anion transport protein	Band 3 anion transport protein	Band 3 anion transport protein	Band 3 anion transport protein	Band 3 anion transport protein	Band 3 anion transport protein
96						
97	Beta-2-glycoprotein 1	Beta-2-glycoprotein 1	Beta-2-glycoprotein 1		Beta-2-glycoprotein 1	
98	Beta-2-microglobulin	Beta-2-microglobulin	Beta-2-microglobulin	Beta-2-microglobulin	Beta-2-microglobulin	Beta-2-microglobulin
99	Beta-hexosaminidase subunit beta	Beta-hexosaminidase subunit beta	Beta-hexosaminidase subunit beta		Beta-hexosaminidase subunit beta	Beta-hexosaminidase subunit beta
100	BH3-interacting domain death agonist	BH3-interacting domain death agonist	BH3-interacting domain death agonist	BH3-interacting domain death agonist	BH3-interacting domain death agonist	BH3-interacting domain death agonist
101	Bifunctional glutamate/proline--tRNA ligase	Bifunctional glutamate/proline--tRNA ligase	Bifunctional glutamate/proline--tRNA ligase	Bifunctional glutamate/proline--tRNA ligase	Bifunctional glutamate/proline--tRNA ligase	
102		Bifunctional phosphoribosylaminoimidazole	Bifunctional phosphoribosylaminoimidazole	Bifunctional phosphoribosylaminoimidazole	Bifunctional phosphoribosylaminoimidazole	Bifunctional phosphoribosylaminoimidazole
103	Bisphosphoglycerate mutase	Bisphosphoglycerate mutase	Bisphosphoglycerate mutase		Bisphosphoglycerate mutase	Bisphosphoglycerate mutase
104	Bone marrow proteoglycan	Bone marrow proteoglycan	Bone marrow proteoglycan	Bone marrow proteoglycan	Bone marrow proteoglycan	Bone marrow proteoglycan
105	BPI fold-containing family B member 1	BPI fold-containing family B member 1		BPI fold-containing family B member 1	BPI fold-containing family B member 1	
106	Brain acid soluble protein 1	Brain acid soluble protein 1	Brain acid soluble protein 1	Brain acid soluble protein 1	Brain acid soluble protein 1	Brain acid soluble protein 1
107	Bridging integrator 2	Bridging integrator 2	Bridging integrator 2	Bridging integrator 2	Bridging integrator 2	Bridging integrator 2
108	C-type lectin domain family 12 member A	C-type lectin domain family 12 member A	C-type lectin domain family 12 member A		C-type lectin domain family 12 member A	C-type lectin domain family 12 member A
109	C-X-C motif chemokine 2	C-X-C motif chemokine 2	C-X-C motif chemokine 2	C-X-C motif chemokine 2	C-X-C motif chemokine 2	C-X-C motif chemokine 2
110	Calcium-binding protein 39	Calcium-binding protein 39	Calcium-binding protein 39	Calcium-binding protein 39	Calcium-binding protein 39	Calcium-binding protein 39
111	Calcyclin-binding protein	Calcyclin-binding protein	Calcyclin-binding protein	Calcyclin-binding protein	Calcyclin-binding protein	Calcyclin-binding protein
112		Caldesmon	Caldesmon	Caldesmon	Caldesmon	Caldesmon
113	Calmodulin-1	Calmodulin-1	Calmodulin-1	Calmodulin-1	Calmodulin-1	Calmodulin-1
114	Calmodulin-like protein 5	Calmodulin-like protein 5	Calmodulin-like protein 5	Calmodulin-like protein 5	Calmodulin-like protein 5	Calmodulin-like protein 5
115	Calnexin	Calnexin	Calnexin	Calnexin	Calnexin	Calnexin
116		Calpain small subunit 1	Calpain small subunit 1	Calpain small subunit 1	Calpain small subunit 1	Calpain small subunit 1
117	Calpain-1 catalytic subunit	Calpain-1 catalytic subunit	Calpain-1 catalytic subunit	Calpain-1 catalytic subunit	Calpain-1 catalytic subunit	Calpain-1 catalytic subunit
118	Calpastatin	Calpastatin	Calpastatin	Calpastatin	Calpastatin	Calpastatin
119	Calponin-2	Calponin-2	Calponin-2	Calponin-2	Calponin-2	Calponin-2
120		Calreticulin	Calreticulin	Calreticulin	Calreticulin	Calreticulin
121	cAMP-dependent protein kinase catalytic subunit alpha	cAMP-dependent protein kinase catalytic subunit alpha	cAMP-dependent protein kinase catalytic subunit alpha	cAMP-dependent protein kinase catalytic subunit alpha	cAMP-dependent protein kinase catalytic subunit alpha	cAMP-dependent protein kinase catalytic subunit alpha
122		cAMP-dependent protein kinase type I-alpha regulatory subunit		cAMP-dependent protein kinase type I-alpha regulatory subunit	cAMP-dependent protein kinase type I-alpha regulatory subunit	cAMP-dependent protein kinase type I-alpha regulatory subunit
123	CapZ-interacting protein	CapZ-interacting protein	CapZ-interacting protein	CapZ-interacting protein	CapZ-interacting protein	CapZ-interacting protein
124	Carbonic anhydrase 1	Carbonic anhydrase 1	Carbonic anhydrase 1	Carbonic anhydrase 1	Carbonic anhydrase 1	Carbonic anhydrase 1
125	Carbonic anhydrase 2	Carbonic anhydrase 2	Carbonic anhydrase 2		Carbonic anhydrase 2	Carbonic anhydrase 2
126	Carbonic anhydrase 4	Carbonic anhydrase 4	Carbonic anhydrase 4			Carbonic anhydrase 4

127	Carbonyl reductase [NADPH] 1	Carbonyl reductase [NADPH] 1	Carbonyl reductase [NADPH] 1	Carbonyl reductase [NADPH] 1	Carbonyl reductase [NADPH] 1	Carbonyl reductase [NADPH] 1
128	Carcinoembryonic antigen-related cell adhesion molecule 8	Carcinoembryonic antigen-related cell adhesion molecule 8	Carcinoembryonic antigen-related cell adhesion molecule 8	Carcinoembryonic antigen-related cell adhesion molecule 8	Carcinoembryonic antigen-related cell adhesion molecule 8	Carcinoembryonic antigen-related cell adhesion molecule 8
129	Caspase recruitment domain-containing protein 16	Caspase recruitment domain-containing protein 16	Caspase recruitment domain-containing protein 16		Caspase recruitment domain-containing protein 16	Caspase recruitment domain-containing protein 16
130	Catalase	Catalase	Catalase	Catalase	Catalase	Catalase
131	Cathelicidin antimicrobial peptide	Cathelicidin antimicrobial peptide	Cathelicidin antimicrobial peptide	Cathelicidin antimicrobial peptide	Cathelicidin antimicrobial peptide	Cathelicidin antimicrobial peptide
132	Cathepsin D	Cathepsin D		Cathepsin D	Cathepsin D	
133	Cathepsin G	Cathepsin G	Cathepsin G	Cathepsin G	Cathepsin G	Cathepsin G
134	Cathepsin S	Cathepsin S	Cathepsin S	Cathepsin S	Cathepsin S	Cathepsin S
135	Cathepsin Z	Cathepsin Z	Cathepsin Z	Cathepsin Z	Cathepsin Z	Cathepsin Z
136	Cation-independent mannose-6-phosphate receptor	Cation-independent mannose-6-phosphate receptor	Cation-independent mannose-6-phosphate receptor	Cation-independent mannose-6-phosphate receptor	Cation-independent mannose-6-phosphate receptor	Cation-independent mannose-6-phosphate receptor
137	Caveolae-associated protein 2	Caveolae-associated protein 2	Caveolae-associated protein 2		Caveolae-associated protein 2	Caveolae-associated protein 2
138	CD177 antigen	CD177 antigen	CD177 antigen	CD177 antigen	CD177 antigen	CD177 antigen
139	CD44 antigen	CD44 antigen	CD44 antigen	CD44 antigen	CD44 antigen	CD44 antigen
140	CD59 glycoprotein	CD59 glycoprotein	CD59 glycoprotein	CD59 glycoprotein	CD59 glycoprotein	CD59 glycoprotein
141	Cell division control protein 42 homolog	Cell division control protein 42 homolog	Cell division control protein 42 homolog	Cell division control protein 42 homolog	Cell division control protein 42 homolog	Cell division control protein 42 homolog
142	Charged multivesicular body protein 4b	Charged multivesicular body protein 4b	Charged multivesicular body protein 4b	Charged multivesicular body protein 4b	Charged multivesicular body protein 4b	Charged multivesicular body protein 4b
143	Chitinase-3-like protein 1	Chitinase-3-like protein 1	Chitinase-3-like protein 1	Chitinase-3-like protein 1	Chitinase-3-like protein 1	Chitinase-3-like protein 1
144	Chitotriosidase-1	Chitotriosidase-1	Chitotriosidase-1	Chitotriosidase-1	Chitotriosidase-1	Chitotriosidase-1
145	Chloride intracellular channel protein 1	Chloride intracellular channel protein 1	Chloride intracellular channel protein 1	Chloride intracellular channel protein 1	Chloride intracellular channel protein 1	Chloride intracellular channel protein 1
146	Chromobox protein homolog 3	Chromobox protein homolog 3	Chromobox protein homolog 3	Chromobox protein homolog 3	Chromobox protein homolog 3	Chromobox protein homolog 3
147	Clathrin heavy chain 1	Clathrin heavy chain 1	Clathrin heavy chain 1	Clathrin heavy chain 1	Clathrin heavy chain 1	Clathrin heavy chain 1
148	CMRF35-like molecule 8	CMRF35-like molecule 8	CMRF35-like molecule 8	CMRF35-like molecule 8	CMRF35-like molecule 8	CMRF35-like molecule 8
149	Coactosin-like protein	Coactosin-like protein	Coactosin-like protein	Coactosin-like protein	Coactosin-like protein	Coactosin-like protein
150	Coagulation factor X	Coagulation factor X	Coagulation factor X	Coagulation factor X	Coagulation factor X	Coagulation factor X
151		Coatomer subunit alpha	Coatomer subunit alpha	Coatomer subunit alpha	Coatomer subunit alpha	Coatomer subunit alpha
152		Coatomer subunit delta	Coatomer subunit delta	Coatomer subunit delta	Coatomer subunit delta	Coatomer subunit delta
153			Coatomer subunit epsilon	Coatomer subunit epsilon	Coatomer subunit epsilon	Coatomer subunit epsilon
154	Cofilin-1	Cofilin-1	Cofilin-1	Cofilin-1	Cofilin-1	Cofilin-1
155	Coiled-coil domain-containing protein 88B			Coiled-coil domain-containing protein 88B	Coiled-coil domain-containing protein 88B	Coiled-coil domain-containing protein 88B
156	Collagen alpha-1(I) chain	Collagen alpha-1(I) chain	Collagen alpha-1(I) chain	Collagen alpha-1(I) chain	Collagen alpha-1(I) chain	Collagen alpha-1(I) chain
157	Collagen alpha-2(I) chain	Collagen alpha-2(I) chain	Collagen alpha-2(I) chain	Collagen alpha-2(I) chain	Collagen alpha-2(I) chain	Collagen alpha-2(I) chain
158	Complement C3	Complement C3	Complement C3	Complement C3	Complement C3	Complement C3

159	Complement component C1q receptor	Complement component C1q receptor	Complement component C1q receptor	Complement component C1q receptor	Complement component C1q receptor	Complement component C1q receptor
160	Complement decay-accelerating factor	Complement decay-accelerating factor	Complement decay-accelerating factor	Complement decay-accelerating factor	Complement decay-accelerating factor	Complement decay-accelerating factor
161	Complement factor D		Complement factor D	Complement factor D		Complement factor D
162	Complement receptor type 1	Complement receptor type 1	Complement receptor type 1	Complement receptor type 1	Complement receptor type 1	Complement receptor type 1
163	Copine-3	Copine-3	Copine-3	Copine-3	Copine-3	Copine-3
164				Copper homeostasis protein cutC homolog	Copper homeostasis protein cutC homolog	Copper homeostasis protein cutC homolog
165	Core histone macro-H2A.1	Core histone macro-H2A.1	Core histone macro-H2A.1	Core histone macro-H2A.1	Core histone macro-H2A.1	Core histone macro-H2A.1
166	Coronin-1A	Coronin-1A	Coronin-1A	Coronin-1A	Coronin-1A	Coronin-1A
167	Coronin-1C	Coronin-1C	Coronin-1C	Coronin-1C	Coronin-1C	Coronin-1C
168	CYFIP-related Rac1 interactor B	CYFIP-related Rac1 interactor B	CYFIP-related Rac1 interactor B	CYFIP-related Rac1 interactor B	CYFIP-related Rac1 interactor B	CYFIP-related Rac1 interactor B
169	Cystatin-B	Cystatin-B	Cystatin-B	Cystatin-B	Cystatin-B	Cystatin-B
170	Cystatin-C	Cystatin-C				
171	Cystatin-F	Cystatin-F	Cystatin-F	Cystatin-F	Cystatin-F	Cystatin-F
172	Cysteine and glycine-rich protein 1	Cysteine and glycine-rich protein 1	Cysteine and glycine-rich protein 1	Cysteine and glycine-rich protein 1	Cysteine and glycine-rich protein 1	
173	Cysteine-rich secretory protein 3	Cysteine-rich secretory protein 3	Cysteine-rich secretory protein 3	Cysteine-rich secretory protein 3	Cysteine-rich secretory protein 3	Cysteine-rich secretory protein 3
174	Cysteine-rich secretory protein LCCL domain-containing 2	Cysteine-rich secretory protein LCCL domain-containing 2	Cysteine-rich secretory protein LCCL domain-containing 2	Cysteine-rich secretory protein LCCL domain-containing 2	Cysteine-rich secretory protein LCCL domain-containing 2	Cysteine-rich secretory protein LCCL domain-containing 2
175	Cytidine deaminase	Cytidine deaminase	Cytidine deaminase	Cytidine deaminase	Cytidine deaminase	Cytidine deaminase
176	Cytochrome b-245 heavy chain	Cytochrome b-245 heavy chain	Cytochrome b-245 heavy chain	Cytochrome b-245 heavy chain	Cytochrome b-245 heavy chain	Cytochrome b-245 heavy chain
177	Cytochrome c oxidase subunit 6B1	Cytochrome c oxidase subunit 6B1	Cytochrome c oxidase subunit 6B1	Cytochrome c oxidase subunit 6B1	Cytochrome c oxidase subunit 6B1	Cytochrome c oxidase subunit 6B1
178				Cytoplasmic dynein 1 heavy chain 1	Cytoplasmic dynein 1 heavy chain 1	Cytoplasmic dynein 1 heavy chain 1
179		Cytoplasmic dynein 1 intermediate chain 2		Cytoplasmic dynein 1 intermediate chain 2	Cytoplasmic dynein 1 intermediate chain 2	Cytoplasmic dynein 1 intermediate chain 2
180	Cytosol aminopeptidase	Cytosol aminopeptidase	Cytosol aminopeptidase	Cytosol aminopeptidase	Cytosol aminopeptidase	Cytosol aminopeptidase
181	D-dopachrome decarboxylase	D-dopachrome decarboxylase	D-dopachrome decarboxylase	D-dopachrome decarboxylase	D-dopachrome decarboxylase	D-dopachrome decarboxylase
182	Deleted in malignant brain tumors 1 protein	Deleted in malignant brain tumors 1 protein	Deleted in malignant brain tumors 1 protein	Deleted in malignant brain tumors 1 protein		Deleted in malignant brain tumors 1 protein
183	Delta-aminolevulinic acid dehydratase	Delta-aminolevulinic acid dehydratase	Delta-aminolevulinic acid dehydratase	Delta-aminolevulinic acid dehydratase	Delta-aminolevulinic acid dehydratase	Delta-aminolevulinic acid dehydratase
184		Delta(14)-sterol reductase LBR	Delta(14)-sterol reductase LBR	Delta(14)-sterol reductase LBR	Delta(14)-sterol reductase LBR	
185		Density-regulated protein		Density-regulated protein	Density-regulated protein	Density-regulated protein
186	Dermcidin	Dermcidin	Dermcidin	Dermcidin	Dermcidin	Dermcidin
187	Desmoglein-1	Desmoglein-1	Desmoglein-1	Desmoglein-1		
188	Desmoplakin		Desmoplakin			Desmoplakin
189			Destrin	Destrin	Destrin	
190	Di-N-acetylchitobiase	Di-N-acetylchitobiase	Di-N-acetylchitobiase	Di-N-acetylchitobiase	Di-N-acetylchitobiase	Di-N-acetylchitobiase

191	Differentially expressed in FDCP 6 homolog	Differentially expressed in FDCP 6 homolog	Differentially expressed in FDCP 6 homolog	Differentially expressed in FDCP 6 homolog	Differentially expressed in FDCP 6 homolog	Differentially expressed in FDCP 6 homolog
192	Dihydropteridine reductase	Dihydropteridine reductase	Dihydropteridine reductase	Dihydropteridine reductase	Dihydropteridine reductase	Dihydropteridine reductase
193		Dihydropyrimidinase-related protein 2	Dihydropyrimidinase-related protein 2	Dihydropyrimidinase-related protein 2	Dihydropyrimidinase-related protein 2	Dihydropyrimidinase-related protein 2
194		Dipeptidyl peptidase 1	Dipeptidyl peptidase 1	Dipeptidyl peptidase 1	Dipeptidyl peptidase 1	
195	Disintegrin and metalloproteinase domain-containing protein 8	Disintegrin and metalloproteinase domain-containing protein 8	Disintegrin and metalloproteinase domain-containing protein 8	Disintegrin and metalloproteinase domain-containing protein 8	Disintegrin and metalloproteinase domain-containing protein 8	Disintegrin and metalloproteinase domain-containing protein 8
196			DNA damage-binding protein 1	DNA damage-binding protein 1		DNA damage-binding protein 1
197	DNA-(apurinic or apyrimidinic site) endonuclease	DNA-(apurinic or apyrimidinic site) endonuclease	DNA-(apurinic or apyrimidinic site) endonuclease	DNA-(apurinic or apyrimidinic site) endonuclease	DNA-(apurinic or apyrimidinic site) endonuclease	DNA-(apurinic or apyrimidinic site) endonuclease
198		Drebrin-like protein	Drebrin-like protein	Drebrin-like protein	Drebrin-like protein	Drebrin-like protein
199		Dual specificity mitogen-activated protein kinase kinase 1	Dual specificity mitogen-activated protein kinase kinase 1	Dual specificity mitogen-activated protein kinase kinase 1	Dual specificity mitogen-activated protein kinase kinase 1	
200		Dual specificity mitogen-activated protein kinase kinase 2	Dual specificity mitogen-activated protein kinase kinase 2	Dual specificity mitogen-activated protein kinase kinase 2	Dual specificity mitogen-activated protein kinase kinase 2	Dual specificity mitogen-activated protein kinase kinase 2
201	Dynamin-2	Dynamin-2	Dynamin-2	Dynamin-2	Dynamin-2	Dynamin-2
202				Dynein light chain 2, cytoplasmic	Dynein light chain 2, cytoplasmic	Dynein light chain 2, cytoplasmic
203		Dynein light chain roadblock-type 2	Dynein light chain roadblock-type 2	Dynein light chain roadblock-type 2	Dynein light chain roadblock-type 2	Dynein light chain roadblock-type 2
204	EF-hand domain-containing protein D2	EF-hand domain-containing protein D2	EF-hand domain-containing protein D2	EF-hand domain-containing protein D2	EF-hand domain-containing protein D2	EF-hand domain-containing protein D2
205	EH domain-containing protein 1	EH domain-containing protein 1	EH domain-containing protein 1	EH domain-containing protein 1	EH domain-containing protein 1	EH domain-containing protein 1
206	Elongation factor 1-alpha 1	Elongation factor 1-alpha 1	Elongation factor 1-alpha 1	Elongation factor 1-alpha 1	Elongation factor 1-alpha 1	Elongation factor 1-alpha 1
207	Elongation factor 1-delta	Elongation factor 1-delta	Elongation factor 1-delta	Elongation factor 1-delta	Elongation factor 1-delta	Elongation factor 1-delta
208	Elongation factor 1-gamma	Elongation factor 1-gamma	Elongation factor 1-gamma	Elongation factor 1-gamma	Elongation factor 1-gamma	Elongation factor 1-gamma
209	Elongation factor 2	Elongation factor 2	Elongation factor 2	Elongation factor 2	Elongation factor 2	Elongation factor 2
210	Endoplasmic reticulum chaperone BiP	Endoplasmic reticulum chaperone BiP	Endoplasmic reticulum chaperone BiP	Endoplasmic reticulum chaperone BiP	Endoplasmic reticulum chaperone BiP	Endoplasmic reticulum chaperone BiP
211	Endoplasmic reticulum resident protein 44	Endoplasmic reticulum resident protein 44	Endoplasmic reticulum resident protein 44	Endoplasmic reticulum resident protein 44	Endoplasmic reticulum resident protein 44	Endoplasmic reticulum resident protein 44
212	Endoplasmin	Endoplasmin	Endoplasmin	Endoplasmin	Endoplasmin	Endoplasmin
213	Enhancer of rudimentary homolog	Enhancer of rudimentary homolog	Enhancer of rudimentary homolog	Enhancer of rudimentary homolog	Enhancer of rudimentary homolog	Enhancer of rudimentary homolog
214	Eosinophil cationic protein	Eosinophil cationic protein	Eosinophil cationic protein	Eosinophil cationic protein	Eosinophil cationic protein	Eosinophil cationic protein
215	Eosinophil peroxidase	Eosinophil peroxidase	Eosinophil peroxidase	Eosinophil peroxidase	Eosinophil peroxidase	Eosinophil peroxidase
216	Ester hydrolase C11orf54	Ester hydrolase C11orf54	Ester hydrolase C11orf54	Ester hydrolase C11orf54	Ester hydrolase C11orf54	Ester hydrolase C11orf54
217				Eukaryotic translation initiation factor 2 subunit 1	Eukaryotic translation initiation factor 2 subunit 1	Eukaryotic translation initiation factor 2 subunit 1
218	Eukaryotic translation initiation factor 2 subunit 2	Eukaryotic translation initiation factor 2 subunit 2	Eukaryotic translation initiation factor 2 subunit 2	Eukaryotic translation initiation factor 2 subunit 2	Eukaryotic translation initiation factor 2 subunit 2	Eukaryotic translation initiation factor 2 subunit 2
219	Eukaryotic translation initiation factor 3 subunit A	Eukaryotic translation initiation factor 3 subunit A	Eukaryotic translation initiation factor 3 subunit A	Eukaryotic translation initiation factor 3 subunit A	Eukaryotic translation initiation factor 3 subunit A	Eukaryotic translation initiation factor 3 subunit A
220	Eukaryotic translation initiation factor 3 subunit J	Eukaryotic translation initiation factor 3 subunit J	Eukaryotic translation initiation factor 3 subunit J	Eukaryotic translation initiation factor 3 subunit J	Eukaryotic translation initiation factor 3 subunit J	Eukaryotic translation initiation factor 3 subunit J
221	Eukaryotic translation initiation factor 4B	Eukaryotic translation initiation factor 4B	Eukaryotic translation initiation factor 4B		Eukaryotic translation initiation factor 4B	Eukaryotic translation initiation factor 4B
222	Eukaryotic translation initiation factor 5A-1	Eukaryotic translation initiation factor 5A-1	Eukaryotic translation initiation factor 5A-1	Eukaryotic translation initiation factor 5A-1		Eukaryotic translation initiation factor 5A-1

223	Extended synaptotagmin-1	Extended synaptotagmin-1	Extended synaptotagmin-1	Extended synaptotagmin-1	Extended synaptotagmin-1	Extended synaptotagmin-1
224	Ezrin	Ezrin	Ezrin	Ezrin	Ezrin	Ezrin
225	F-actin-capping protein subunit alpha-1	F-actin-capping protein subunit alpha-1	F-actin-capping protein subunit alpha-1	F-actin-capping protein subunit alpha-1	F-actin-capping protein subunit alpha-1	F-actin-capping protein subunit alpha-1
226	F-actin-capping protein subunit alpha-2	F-actin-capping protein subunit alpha-2	F-actin-capping protein subunit alpha-2	F-actin-capping protein subunit alpha-2	F-actin-capping protein subunit alpha-2	F-actin-capping protein subunit alpha-2
227	F-actin-capping protein subunit beta	F-actin-capping protein subunit beta	F-actin-capping protein subunit beta	F-actin-capping protein subunit beta	F-actin-capping protein subunit beta	F-actin-capping protein subunit beta
228		Far upstream element-binding protein 1		Far upstream element-binding protein 1	Far upstream element-binding protein 1	Far upstream element-binding protein 1
229	Fatty acid-binding protein 5	Fatty acid-binding protein 5	Fatty acid-binding protein 5	Fatty acid-binding protein 5	Fatty acid-binding protein 5	Fatty acid-binding protein 5
230			Fermitin family homolog 3	Fermitin family homolog 3	Fermitin family homolog 3	Fermitin family homolog 3
231	Fibrinogen alpha chain	Fibrinogen alpha chain	Fibrinogen alpha chain	Fibrinogen alpha chain	Fibrinogen alpha chain	Fibrinogen alpha chain
232	Fibrinogen beta chain	Fibrinogen beta chain	Fibrinogen beta chain	Fibrinogen beta chain	Fibrinogen beta chain	Fibrinogen beta chain
233	Fibrinogen gamma chain	Fibrinogen gamma chain	Fibrinogen gamma chain	Fibrinogen gamma chain	Fibrinogen gamma chain	Fibrinogen gamma chain
234	Fibroleukin	Fibroleukin	Fibroleukin	Fibroleukin	Fibroleukin	Fibroleukin
235	Ficolin-1	Ficolin-1	Ficolin-1	Ficolin-1	Ficolin-1	Ficolin-1
236		Filaggrin	Filaggrin		Filaggrin	Filaggrin
237	Filaggrin-2	Filaggrin-2	Filaggrin-2			
238	Filamin-A	Filamin-A	Filamin-A	Filamin-A	Filamin-A	Filamin-A
239		FK506-binding protein 15		FK506-binding protein 15	FK506-binding protein 15	FK506-binding protein 15
240	Flavin reductase (NADPH)	Flavin reductase (NADPH)	Flavin reductase (NADPH)	Flavin reductase (NADPH)	Flavin reductase (NADPH)	Flavin reductase (NADPH)
241	Formin-like protein 1	Formin-like protein 1	Formin-like protein 1	Formin-like protein 1	Formin-like protein 1	Formin-like protein 1
242	Fructose-1,6-bisphosphatase 1	Fructose-1,6-bisphosphatase 1	Fructose-1,6-bisphosphatase 1	Fructose-1,6-bisphosphatase 1	Fructose-1,6-bisphosphatase 1	Fructose-1,6-bisphosphatase 1
243	Fructose-bisphosphate aldolase A	Fructose-bisphosphate aldolase A	Fructose-bisphosphate aldolase A	Fructose-bisphosphate aldolase A	Fructose-bisphosphate aldolase A	Fructose-bisphosphate aldolase A
244	Fructose-bisphosphate aldolase C	Fructose-bisphosphate aldolase C	Fructose-bisphosphate aldolase C	Fructose-bisphosphate aldolase C	Fructose-bisphosphate aldolase C	Fructose-bisphosphate aldolase C
245	Fumarylacetoacetase	Fumarylacetoacetase	Fumarylacetoacetase	Fumarylacetoacetase	Fumarylacetoacetase	Fumarylacetoacetase
246		FYN-binding protein 1	FYN-binding protein 1	FYN-binding protein 1	FYN-binding protein 1	FYN-binding protein 1
247	Galectin-1	Galectin-1	Galectin-1	Galectin-1	Galectin-1	Galectin-1
248	Galectin-10	Galectin-10	Galectin-10	Galectin-10	Galectin-10	Galectin-10
249	Galectin-3	Galectin-3	Galectin-3	Galectin-3	Galectin-3	Galectin-3
250		Gamma-glutamyl hydrolase		Gamma-glutamyl hydrolase	Gamma-glutamyl hydrolase	Gamma-glutamyl hydrolase
251	Gamma-glutamylcyclotransferase	Gamma-glutamylcyclotransferase	Gamma-glutamylcyclotransferase	Gamma-glutamylcyclotransferase	Gamma-glutamylcyclotransferase	Gamma-glutamylcyclotransferase
252	Gamma-interferon-inducible protein 16	Gamma-interferon-inducible protein 16	Gamma-interferon-inducible protein 16	Gamma-interferon-inducible protein 16	Gamma-interferon-inducible protein 16	Gamma-interferon-inducible protein 16
253		Gamma-parvin	Gamma-parvin	Gamma-parvin	Gamma-parvin	Gamma-parvin
254	GDP-L-fucose synthase	GDP-L-fucose synthase	GDP-L-fucose synthase	GDP-L-fucose synthase	GDP-L-fucose synthase	GDP-L-fucose synthase

255	Gelsolin	Gelsolin	Gelsolin	Gelsolin	Gelsolin	Gelsolin
256			GEM-interacting protein		GEM-interacting protein	
257	Glia maturation factor gamma	Glia maturation factor gamma	Glia maturation factor gamma	Glia maturation factor gamma	Glia maturation factor gamma	Glia maturation factor gamma
258	Glucosamine-6-phosphate isomerase 1	Glucosamine-6-phosphate isomerase 1	Glucosamine-6-phosphate isomerase 1	Glucosamine-6-phosphate isomerase 1	Glucosamine-6-phosphate isomerase 1	Glucosamine-6-phosphate isomerase 1
259	Glucose-6-phosphate 1-dehydrogenase	Glucose-6-phosphate 1-dehydrogenase	Glucose-6-phosphate 1-dehydrogenase	Glucose-6-phosphate 1-dehydrogenase	Glucose-6-phosphate 1-dehydrogenase	Glucose-6-phosphate 1-dehydrogenase
260	Glucose-6-phosphate isomerase	Glucose-6-phosphate isomerase	Glucose-6-phosphate isomerase	Glucose-6-phosphate isomerase	Glucose-6-phosphate isomerase	Glucose-6-phosphate isomerase
261		Glucosidase 2 subunit beta		Glucosidase 2 subunit beta	Glucosidase 2 subunit beta	Glucosidase 2 subunit beta
262	Glutaminyl-peptide cyclotransferase	Glutaminyl-peptide cyclotransferase	Glutaminyl-peptide cyclotransferase	Glutaminyl-peptide cyclotransferase	Glutaminyl-peptide cyclotransferase	Glutaminyl-peptide cyclotransferase
263	Glutaredoxin-1	Glutaredoxin-1	Glutaredoxin-1	Glutaredoxin-1	Glutaredoxin-1	Glutaredoxin-1
264	Glutathione reductase, mitochondrial	Glutathione reductase, mitochondrial	Glutathione reductase, mitochondrial	Glutathione reductase, mitochondrial	Glutathione reductase, mitochondrial	Glutathione reductase, mitochondrial
265				Glutathione S-transferase Mu 4		
266	Glutathione S-transferase omega-1	Glutathione S-transferase omega-1	Glutathione S-transferase omega-1	Glutathione S-transferase omega-1	Glutathione S-transferase omega-1	Glutathione S-transferase omega-1
267	Glutathione S-transferase P	Glutathione S-transferase P	Glutathione S-transferase P	Glutathione S-transferase P	Glutathione S-transferase P	Glutathione S-transferase P
268	Glyceraldehyde-3-phosphate dehydrogenase	Glyceraldehyde-3-phosphate dehydrogenase	Glyceraldehyde-3-phosphate dehydrogenase	Glyceraldehyde-3-phosphate dehydrogenase	Glyceraldehyde-3-phosphate dehydrogenase	Glyceraldehyde-3-phosphate dehydrogenase
269	Glycogen [starch] synthase, muscle	Glycogen [starch] synthase, muscle	Glycogen [starch] synthase, muscle	Glycogen [starch] synthase, muscle	Glycogen [starch] synthase, muscle	Glycogen [starch] synthase, muscle
270	Glycogen phosphorylase, liver form	Glycogen phosphorylase, liver form	Glycogen phosphorylase, liver form	Glycogen phosphorylase, liver form	Glycogen phosphorylase, liver form	Glycogen phosphorylase, liver form
271	Glycogenin-1	Glycogenin-1	Glycogenin-1	Glycogenin-1	Glycogenin-1	Glycogenin-1
272	Glyoxalase domain-containing protein 4	Glyoxalase domain-containing protein 4	Glyoxalase domain-containing protein 4	Glyoxalase domain-containing protein 4	Glyoxalase domain-containing protein 4	Glyoxalase domain-containing protein 4
273					Golgi apparatus protein 1	
274	Golgi-associated plant pathogenesis-related protein 1	Golgi-associated plant pathogenesis-related protein 1	Golgi-associated plant pathogenesis-related protein 1	Golgi-associated plant pathogenesis-related protein 1	Golgi-associated plant pathogenesis-related protein 1	Golgi-associated plant pathogenesis-related protein 1
275	Grancalcin	Grancalcin	Grancalcin	Grancalcin	Grancalcin	Grancalcin
276	Growth factor receptor-bound protein 2	Growth factor receptor-bound protein 2	Growth factor receptor-bound protein 2	Growth factor receptor-bound protein 2	Growth factor receptor-bound protein 2	Growth factor receptor-bound protein 2
277	GTP-binding nuclear protein Ran	GTP-binding nuclear protein Ran	GTP-binding nuclear protein Ran	GTP-binding nuclear protein Ran	GTP-binding nuclear protein Ran	GTP-binding nuclear protein Ran
278	GTPase IMAP family member 4	GTPase IMAP family member 4		GTPase IMAP family member 4	GTPase IMAP family member 4	GTPase IMAP family member 4
279	Guanine nucleotide-binding protein G(i) subunit alpha-2	Guanine nucleotide-binding protein G(i) subunit alpha-2	Guanine nucleotide-binding protein G(i) subunit alpha-2	Guanine nucleotide-binding protein G(i) subunit alpha-2	Guanine nucleotide-binding protein G(i) subunit alpha-2	Guanine nucleotide-binding protein G(i) subunit alpha-2
280	Guanine nucleotide-binding protein G(I)/G(S)/G(T) subunit beta-2	Guanine nucleotide-binding protein G(I)/G(S)/G(T) subunit beta-2	Guanine nucleotide-binding protein G(I)/G(S)/G(T) subunit beta-2		Guanine nucleotide-binding protein G(I)/G(S)/G(T) subunit beta-2	
281		Haloacid dehalogenase-like hydrolase domain-containing protein 2	Haloacid dehalogenase-like hydrolase domain-containing protein 2	Haloacid dehalogenase-like hydrolase domain-containing protein 2	Haloacid dehalogenase-like hydrolase domain-containing protein 2	Haloacid dehalogenase-like hydrolase domain-containing protein 2
282	Haptoglobin	Haptoglobin	Haptoglobin	Haptoglobin	Haptoglobin	Haptoglobin
283	Heat shock 70 kDa protein 1B	Heat shock 70 kDa protein 1B	Heat shock 70 kDa protein 1B	Heat shock 70 kDa protein 1B	Heat shock 70 kDa protein 1B	Heat shock 70 kDa protein 1B
284		Heat shock 70 kDa protein 4		Heat shock 70 kDa protein 4	Heat shock 70 kDa protein 4	Heat shock 70 kDa protein 4
285	Heat shock cognate 71 kDa protein	Heat shock cognate 71 kDa protein	Heat shock cognate 71 kDa protein	Heat shock cognate 71 kDa protein	Heat shock cognate 71 kDa protein	Heat shock cognate 71 kDa protein
286	Heat shock protein beta-1	Heat shock protein beta-1			Heat shock protein beta-1	Heat shock protein beta-1

287	Heat shock protein HSP 90-alpha	Heat shock protein HSP 90-alpha	Heat shock protein HSP 90-alpha	Heat shock protein HSP 90-alpha	Heat shock protein HSP 90-alpha	Heat shock protein HSP 90-alpha
288	Heat shock protein HSP 90-beta	Heat shock protein HSP 90-beta	Heat shock protein HSP 90-beta	Heat shock protein HSP 90-beta	Heat shock protein HSP 90-beta	Heat shock protein HSP 90-beta
289	Hematopoietic lineage cell-specific protein	Hematopoietic lineage cell-specific protein	Hematopoietic lineage cell-specific protein	Hematopoietic lineage cell-specific protein	Hematopoietic lineage cell-specific protein	Hematopoietic lineage cell-specific protein
290				Heme-binding protein 2	Heme-binding protein 2	Heme-binding protein 2
291	Hemoglobin subunit alpha	Hemoglobin subunit alpha	Hemoglobin subunit alpha	Hemoglobin subunit alpha	Hemoglobin subunit alpha	Hemoglobin subunit alpha
292	Hemoglobin subunit beta	Hemoglobin subunit beta	Hemoglobin subunit beta	Hemoglobin subunit beta	Hemoglobin subunit beta	Hemoglobin subunit beta
293	Hemoglobin subunit delta	Hemoglobin subunit delta	Hemoglobin subunit delta	Hemoglobin subunit delta	Hemoglobin subunit delta	Hemoglobin subunit delta
294	Hemoglobin subunit gamma-1	Hemoglobin subunit gamma-1			Hemoglobin subunit gamma-1	
295	Hemopexin	Hemopexin	Hemopexin	Hemopexin	Hemopexin	Hemopexin
296	Hepatoma-derived growth factor	Hepatoma-derived growth factor	Hepatoma-derived growth factor	Hepatoma-derived growth factor	Hepatoma-derived growth factor	Hepatoma-derived growth factor
297	Heterochromatin protein 1-binding protein 3	Heterochromatin protein 1-binding protein 3	Heterochromatin protein 1-binding protein 3	Heterochromatin protein 1-binding protein 3	Heterochromatin protein 1-binding protein 3	Heterochromatin protein 1-binding protein 3
298	Heterogeneous nuclear ribonucleoprotein A1	Heterogeneous nuclear ribonucleoprotein A1	Heterogeneous nuclear ribonucleoprotein A1	Heterogeneous nuclear ribonucleoprotein A1	Heterogeneous nuclear ribonucleoprotein A1	Heterogeneous nuclear ribonucleoprotein A1
299	Heterogeneous nuclear ribonucleoprotein A3	Heterogeneous nuclear ribonucleoprotein A3	Heterogeneous nuclear ribonucleoprotein A3	Heterogeneous nuclear ribonucleoprotein A3	Heterogeneous nuclear ribonucleoprotein A3	Heterogeneous nuclear ribonucleoprotein A3
300	Heterogeneous nuclear ribonucleoprotein D0	Heterogeneous nuclear ribonucleoprotein D0	Heterogeneous nuclear ribonucleoprotein D0	Heterogeneous nuclear ribonucleoprotein D0	Heterogeneous nuclear ribonucleoprotein D0	Heterogeneous nuclear ribonucleoprotein D0
301	Heterogeneous nuclear ribonucleoprotein K	Heterogeneous nuclear ribonucleoprotein K	Heterogeneous nuclear ribonucleoprotein K	Heterogeneous nuclear ribonucleoprotein K	Heterogeneous nuclear ribonucleoprotein K	Heterogeneous nuclear ribonucleoprotein K
302	Heterogeneous nuclear ribonucleoprotein L	Heterogeneous nuclear ribonucleoprotein L	Heterogeneous nuclear ribonucleoprotein L	Heterogeneous nuclear ribonucleoprotein L	Heterogeneous nuclear ribonucleoprotein L	Heterogeneous nuclear ribonucleoprotein L
303	Heterogeneous nuclear ribonucleoprotein M	Heterogeneous nuclear ribonucleoprotein M	Heterogeneous nuclear ribonucleoprotein M	Heterogeneous nuclear ribonucleoprotein M	Heterogeneous nuclear ribonucleoprotein M	Heterogeneous nuclear ribonucleoprotein M
304	Heterogeneous nuclear ribonucleoprotein U	Heterogeneous nuclear ribonucleoprotein U	Heterogeneous nuclear ribonucleoprotein U	Heterogeneous nuclear ribonucleoprotein U	Heterogeneous nuclear ribonucleoprotein U	Heterogeneous nuclear ribonucleoprotein U
305		Heterogeneous nuclear ribonucleoprotein U-like protein 1		Heterogeneous nuclear ribonucleoprotein U-like protein 1	Heterogeneous nuclear ribonucleoprotein U-like protein 1	Heterogeneous nuclear ribonucleoprotein U-like protein 1
306		Heterogeneous nuclear ribonucleoprotein U-like protein 2				
307	Heterogeneous nuclear ribonucleoproteins A2/B1	Heterogeneous nuclear ribonucleoproteins A2/B1	Heterogeneous nuclear ribonucleoproteins A2/B1	Heterogeneous nuclear ribonucleoproteins A2/B1	Heterogeneous nuclear ribonucleoproteins A2/B1	Heterogeneous nuclear ribonucleoproteins A2/B1
308	Heterogeneous nuclear ribonucleoproteins C1/C2	Heterogeneous nuclear ribonucleoproteins C1/C2	Heterogeneous nuclear ribonucleoproteins C1/C2	Heterogeneous nuclear ribonucleoproteins C1/C2	Heterogeneous nuclear ribonucleoproteins C1/C2	Heterogeneous nuclear ribonucleoproteins C1/C2
309	Hexokinase-3	Hexokinase-3	Hexokinase-3	Hexokinase-3	Hexokinase-3	Hexokinase-3
310	High mobility group nucleosome-binding domain-containing protein 3	High mobility group nucleosome-binding domain-containing protein 3	High mobility group nucleosome-binding domain-containing protein 3	High mobility group nucleosome-binding domain-containing protein 3	High mobility group nucleosome-binding domain-containing protein 3	High mobility group nucleosome-binding domain-containing protein 3
311	High mobility group nucleosome-binding domain-containing protein 4	High mobility group nucleosome-binding domain-containing protein 4	High mobility group nucleosome-binding domain-containing protein 4	High mobility group nucleosome-binding domain-containing protein 4	High mobility group nucleosome-binding domain-containing protein 4	
312	High mobility group nucleosome-binding domain-containing protein 5	High mobility group nucleosome-binding domain-containing protein 5				
313	High mobility group protein B1	High mobility group protein B1	High mobility group protein B1	High mobility group protein B1	High mobility group protein B1	High mobility group protein B1
314	High mobility group protein B2	High mobility group protein B2	High mobility group protein B2	High mobility group protein B2	High mobility group protein B2	High mobility group protein B2
315	High mobility group protein B3	High mobility group protein B3	High mobility group protein B3	High mobility group protein B3	High mobility group protein B3	High mobility group protein B3
316	High mobility group protein HMG-I/HMG-Y	High mobility group protein HMG-I/HMG-Y	High mobility group protein HMG-I/HMG-Y	High mobility group protein HMG-I/HMG-Y	High mobility group protein HMG-I/HMG-Y	High mobility group protein HMG-I/HMG-Y
317	Histone H1.0	Histone H1.0	Histone H1.0	Histone H1.0	Histone H1.0	Histone H1.0
318	Histone H1.10	Histone H1.10	Histone H1.10	Histone H1.10	Histone H1.10	Histone H1.10

319	Histone H1.2	Histone H1.2	Histone H1.2	Histone H1.2	Histone H1.2	Histone H1.2
320	Histone H1.3	Histone H1.3	Histone H1.3	Histone H1.3	Histone H1.3	Histone H1.3
321	Histone H1.4	Histone H1.4	Histone H1.4	Histone H1.4	Histone H1.4	Histone H1.4
322	Histone H1.5	Histone H1.5	Histone H1.5	Histone H1.5	Histone H1.5	Histone H1.5
323	Histone H2A type 2-C	Histone H2A type 2-C	Histone H2A type 2-C	Histone H2A type 2-C	Histone H2A type 2-C	Histone H2A type 2-C
324	Histone H2A type 3	Histone H2A type 3	Histone H2A type 3	Histone H2A type 3	Histone H2A type 3	Histone H2A type 3
325	Histone H2A.V	Histone H2A.V	Histone H2A.V	Histone H2A.V	Histone H2A.V	Histone H2A.V
326	Histone H2AX	Histone H2AX	Histone H2AX	Histone H2AX	Histone H2AX	Histone H2AX
327	Histone H2B type 1-K	Histone H2B type 1-K	Histone H2B type 1-K	Histone H2B type 1-K	Histone H2B type 1-K	Histone H2B type 1-K
328	Histone H3-7	Histone H3-7	Histone H3-7	Histone H3-7	Histone H3-7	Histone H3-7
329	Histone H3.1	Histone H3.1	Histone H3.1	Histone H3.1	Histone H3.1	Histone H3.1
330	Histone H4	Histone H4	Histone H4	Histone H4	Histone H4	Histone H4
331				Histone-binding protein RBBP4	Histone-binding protein RBBP4	Histone-binding protein RBBP4
332			HLA class I histocompatibility antigen, B alpha chain	HLA class I histocompatibility antigen, B alpha chain	HLA class I histocompatibility antigen, B alpha chain	
333	Hornerin	Hornerin	Hornerin	Hornerin	Hornerin	Hornerin
334	Hsp90 co-chaperone Cdc37	Hsp90 co-chaperone Cdc37	Hsp90 co-chaperone Cdc37	Hsp90 co-chaperone Cdc37	Hsp90 co-chaperone Cdc37	Hsp90 co-chaperone Cdc37
335	Hydroxyacylglutathione hydrolase, mitochondrial	Hydroxyacylglutathione hydrolase, mitochondrial	Hydroxyacylglutathione hydrolase, mitochondrial	Hydroxyacylglutathione hydrolase, mitochondrial	Hydroxyacylglutathione hydrolase, mitochondrial	Hydroxyacylglutathione hydrolase, mitochondrial
336	Hypoxanthine-guanine phosphoribosyltransferase	Hypoxanthine-guanine phosphoribosyltransferase	Hypoxanthine-guanine phosphoribosyltransferase	Hypoxanthine-guanine phosphoribosyltransferase	Hypoxanthine-guanine phosphoribosyltransferase	Hypoxanthine-guanine phosphoribosyltransferase
337		Hypoxia up-regulated protein 1	Hypoxia up-regulated protein 1	Hypoxia up-regulated protein 1	Hypoxia up-regulated protein 1	Hypoxia up-regulated protein 1
338	Immunoglobulin heavy constant alpha 1	Immunoglobulin heavy constant alpha 1	Immunoglobulin heavy constant alpha 1	Immunoglobulin heavy constant alpha 1	Immunoglobulin heavy constant alpha 1	Immunoglobulin heavy constant alpha 1
339	Immunoglobulin heavy constant gamma 1	Immunoglobulin heavy constant gamma 1	Immunoglobulin heavy constant gamma 1	Immunoglobulin heavy constant gamma 1	Immunoglobulin heavy constant gamma 1	Immunoglobulin heavy constant gamma 1
340	Immunoglobulin heavy constant gamma 2	Immunoglobulin heavy constant gamma 2	Immunoglobulin heavy constant gamma 2	Immunoglobulin heavy constant gamma 2	Immunoglobulin heavy constant gamma 2	Immunoglobulin heavy constant gamma 2
341	Immunoglobulin heavy constant gamma 3	Immunoglobulin heavy constant gamma 3	Immunoglobulin heavy constant gamma 3	Immunoglobulin heavy constant gamma 3	Immunoglobulin heavy constant gamma 3	Immunoglobulin heavy constant gamma 3
342	Immunoglobulin heavy constant mu		Immunoglobulin heavy constant mu	Immunoglobulin heavy constant mu	Immunoglobulin heavy constant mu	Immunoglobulin heavy constant mu
343	Immunoglobulin J chain	Immunoglobulin J chain				
344	Immunoglobulin kappa constant	Immunoglobulin kappa constant	Immunoglobulin kappa constant	Immunoglobulin kappa constant	Immunoglobulin kappa constant	Immunoglobulin kappa constant
345	Immunoglobulin lambda constant 2			Immunoglobulin lambda constant 2		Immunoglobulin lambda constant 2
346	Immunoglobulin superfamily member 2	Immunoglobulin superfamily member 2	Immunoglobulin superfamily member 2	Immunoglobulin superfamily member 2		Immunoglobulin superfamily member 2
347					Importin subunit beta-1	Importin subunit beta-1
348		Inositol monophosphatase 1	Inositol monophosphatase 1	Inositol monophosphatase 1	Inositol monophosphatase 1	Inositol monophosphatase 1
349	Integrin alpha-IIb	Integrin alpha-IIb	Integrin alpha-IIb	Integrin alpha-IIb	Integrin alpha-IIb	Integrin alpha-IIb
350	Integrin alpha-M	Integrin alpha-M	Integrin alpha-M	Integrin alpha-M	Integrin alpha-M	Integrin alpha-M

351	Integrin beta-2	Integrin beta-2	Integrin beta-2	Integrin beta-2	Integrin beta-2	Integrin beta-2
352	Integrin beta-3	Integrin beta-3	Integrin beta-3		Integrin beta-3	Integrin beta-3
353	Intercellular adhesion molecule 3	Intercellular adhesion molecule 3	Intercellular adhesion molecule 3	Intercellular adhesion molecule 3	Intercellular adhesion molecule 3	Intercellular adhesion molecule 3
354				Interferon-induced 35 kDa protein	Interferon-induced 35 kDa protein	Interferon-induced 35 kDa protein
355	Interleukin-18		Interleukin-18	Interleukin-18	Interleukin-18	Interleukin-18
356	Interleukin-8	Interleukin-8	Interleukin-8	Interleukin-8	Interleukin-8	Interleukin-8
357		Isocitrate dehydrogenase [NADP] cytoplasmic		Isocitrate dehydrogenase [NADP] cytoplasmic	Isocitrate dehydrogenase [NADP] cytoplasmic	Isocitrate dehydrogenase [NADP] cytoplasmic
358		Jupiter microtubule associated homolog 1		Jupiter microtubule associated homolog 1	Jupiter microtubule associated homolog 1	Jupiter microtubule associated homolog 1
359	Keratin, type I cytoskeletal 10	Keratin, type I cytoskeletal 10	Keratin, type I cytoskeletal 10	Keratin, type I cytoskeletal 10	Keratin, type I cytoskeletal 10	Keratin, type I cytoskeletal 10
360		Keratin, type I cytoskeletal 13				
361	Keratin, type I cytoskeletal 14	Keratin, type I cytoskeletal 14	Keratin, type I cytoskeletal 14	Keratin, type I cytoskeletal 14	Keratin, type I cytoskeletal 14	Keratin, type I cytoskeletal 14
362	Keratin, type I cytoskeletal 16	Keratin, type I cytoskeletal 16	Keratin, type I cytoskeletal 16	Keratin, type I cytoskeletal 16	Keratin, type I cytoskeletal 16	
363	Keratin, type I cytoskeletal 17	Keratin, type I cytoskeletal 17	Keratin, type I cytoskeletal 17		Keratin, type I cytoskeletal 17	
364	Keratin, type I cytoskeletal 9	Keratin, type I cytoskeletal 9	Keratin, type I cytoskeletal 9	Keratin, type I cytoskeletal 9	Keratin, type I cytoskeletal 9	Keratin, type I cytoskeletal 9
365	Keratin, type II cytoskeletal 1	Keratin, type II cytoskeletal 1	Keratin, type II cytoskeletal 1	Keratin, type II cytoskeletal 1	Keratin, type II cytoskeletal 1	Keratin, type II cytoskeletal 1
366	Keratin, type II cytoskeletal 2 epidermal	Keratin, type II cytoskeletal 2 epidermal	Keratin, type II cytoskeletal 2 epidermal	Keratin, type II cytoskeletal 2 epidermal	Keratin, type II cytoskeletal 2 epidermal	Keratin, type II cytoskeletal 2 epidermal
367		Keratin, type II cytoskeletal 4	Keratin, type II cytoskeletal 4	Keratin, type II cytoskeletal 4		
368	Keratin, type II cytoskeletal 5	Keratin, type II cytoskeletal 5	Keratin, type II cytoskeletal 5	Keratin, type II cytoskeletal 5	Keratin, type II cytoskeletal 5	Keratin, type II cytoskeletal 5
369	Keratin, type II cytoskeletal 6A	Keratin, type II cytoskeletal 6A	Keratin, type II cytoskeletal 6A	Keratin, type II cytoskeletal 6A	Keratin, type II cytoskeletal 6A	Keratin, type II cytoskeletal 6A
370	Keratinocyte proline-rich protein	Keratinocyte proline-rich protein		Keratinocyte proline-rich protein	Keratinocyte proline-rich protein	Keratinocyte proline-rich protein
371		KH domain-containing, RNA-binding, signal transduction-associated protein 1	KH domain-containing, RNA-binding, signal transduction-associated protein 1	KH domain-containing, RNA-binding, signal transduction-associated protein 1	KH domain-containing, RNA-binding, signal transduction-associated protein 1	KH domain-containing, RNA-binding, signal transduction-associated protein 1
372		Kinesin-1 heavy chain	Kinesin-1 heavy chain	Kinesin-1 heavy chain	Kinesin-1 heavy chain	
373	Kininogen-1		Kininogen-1		Kininogen-1	Kininogen-1
374	L-lactate dehydrogenase A chain	L-lactate dehydrogenase A chain	L-lactate dehydrogenase A chain	L-lactate dehydrogenase A chain	L-lactate dehydrogenase A chain	L-lactate dehydrogenase A chain
375	L-lactate dehydrogenase B chain	L-lactate dehydrogenase B chain	L-lactate dehydrogenase B chain	L-lactate dehydrogenase B chain	L-lactate dehydrogenase B chain	L-lactate dehydrogenase B chain
376	L-selectin	L-selectin	L-selectin	L-selectin	L-selectin	L-selectin
377	Lactotransferrin	Lactotransferrin	Lactotransferrin	Lactotransferrin	Lactotransferrin	Lactotransferrin
378	Lactoylglutathione lyase	Lactoylglutathione lyase	Lactoylglutathione lyase	Lactoylglutathione lyase	Lactoylglutathione lyase	Lactoylglutathione lyase
379	Lamin-B1	Lamin-B1	Lamin-B1	Lamin-B1	Lamin-B1	Lamin-B1
380	Lamin-B2	Lamin-B2	Lamin-B2	Lamin-B2	Lamin-B2	Lamin-B2
381	Lamina-associated polypeptide 2, isoforms beta/gamma	Lamina-associated polypeptide 2, isoforms beta/gamma	Lamina-associated polypeptide 2, isoforms beta/gamma	Lamina-associated polypeptide 2, isoforms beta/gamma	Lamina-associated polypeptide 2, isoforms beta/gamma	Lamina-associated polypeptide 2, isoforms beta/gamma
382	Large ribosomal subunit protein eL13	Large ribosomal subunit protein eL13	Large ribosomal subunit protein eL13	Large ribosomal subunit protein eL13	Large ribosomal subunit protein eL13	Large ribosomal subunit protein eL13

383	Large ribosomal subunit protein eL6	Large ribosomal subunit protein eL6	Large ribosomal subunit protein eL6	Large ribosomal subunit protein eL6	Large ribosomal subunit protein eL6	Large ribosomal subunit protein eL6
384				Large ribosomal subunit protein P2	Large ribosomal subunit protein P2	Large ribosomal subunit protein P2
385	Large ribosomal subunit protein uL11			Large ribosomal subunit protein uL11	Large ribosomal subunit protein uL11	Large ribosomal subunit protein uL11
386	Large ribosomal subunit protein uL23	Large ribosomal subunit protein uL23	Large ribosomal subunit protein uL23	Large ribosomal subunit protein uL23	Large ribosomal subunit protein uL23	Large ribosomal subunit protein uL23
387	Latent-transforming growth factor beta-binding protein 1	Latent-transforming growth factor beta-binding protein 1	Latent-transforming growth factor beta-binding protein 1			
388	Leucine-rich alpha-2-glycoprotein	Leucine-rich alpha-2-glycoprotein	Leucine-rich alpha-2-glycoprotein	Leucine-rich alpha-2-glycoprotein	Leucine-rich alpha-2-glycoprotein	Leucine-rich alpha-2-glycoprotein
389	Leucine-rich repeat flightless-interacting protein 1	Leucine-rich repeat flightless-interacting protein 1	Leucine-rich repeat flightless-interacting protein 1	Leucine-rich repeat flightless-interacting protein 1	Leucine-rich repeat flightless-interacting protein 1	Leucine-rich repeat flightless-interacting protein 1
390	Leukocyte elastase inhibitor	Leukocyte elastase inhibitor	Leukocyte elastase inhibitor	Leukocyte elastase inhibitor	Leukocyte elastase inhibitor	Leukocyte elastase inhibitor
391	Leukocyte immunoglobulin-like receptor subfamily A member 3	Leukocyte immunoglobulin-like receptor subfamily A member 3	Leukocyte immunoglobulin-like receptor subfamily A member 3	Leukocyte immunoglobulin-like receptor subfamily A member 3	Leukocyte immunoglobulin-like receptor subfamily A member 3	Leukocyte immunoglobulin-like receptor subfamily A member 3
392	Leukocyte immunoglobulin-like receptor subfamily A member 6	Leukocyte immunoglobulin-like receptor subfamily A member 6	Leukocyte immunoglobulin-like receptor subfamily A member 6	Leukocyte immunoglobulin-like receptor subfamily A member 6	Leukocyte immunoglobulin-like receptor subfamily A member 6	
393	Leukotriene A-4 hydrolase	Leukotriene A-4 hydrolase	Leukotriene A-4 hydrolase	Leukotriene A-4 hydrolase	Leukotriene A-4 hydrolase	Leukotriene A-4 hydrolase
394	LIM and SH3 domain protein 1	LIM and SH3 domain protein 1	LIM and SH3 domain protein 1	LIM and SH3 domain protein 1	LIM and SH3 domain protein 1	LIM and SH3 domain protein 1
395			Low affinity immunoglobulin gamma Fc region receptor III-A		Low affinity immunoglobulin gamma Fc region receptor III-A	Low affinity immunoglobulin gamma Fc region receptor III-A
396	Low affinity immunoglobulin gamma Fc region receptor III-B	Low affinity immunoglobulin gamma Fc region receptor III-B	Low affinity immunoglobulin gamma Fc region receptor III-B	Low affinity immunoglobulin gamma Fc region receptor III-B	Low affinity immunoglobulin gamma Fc region receptor III-B	Low affinity immunoglobulin gamma Fc region receptor III-B
397	Lymphocyte-specific protein 1	Lymphocyte-specific protein 1	Lymphocyte-specific protein 1	Lymphocyte-specific protein 1	Lymphocyte-specific protein 1	Lymphocyte-specific protein 1
398	Lysosomal alpha-mannosidase	Lysosomal alpha-mannosidase	Lysosomal alpha-mannosidase		Lysosomal alpha-mannosidase	Lysosomal alpha-mannosidase
399	Lysosome-associated membrane glycoprotein 1	Lysosome-associated membrane glycoprotein 1	Lysosome-associated membrane glycoprotein 1	Lysosome-associated membrane glycoprotein 1	Lysosome-associated membrane glycoprotein 1	Lysosome-associated membrane glycoprotein 1
400	Lysozyme C	Lysozyme C	Lysozyme C	Lysozyme C	Lysozyme C	Lysozyme C
401	m7GpppX diphosphatase	m7GpppX diphosphatase	m7GpppX diphosphatase	m7GpppX diphosphatase	m7GpppX diphosphatase	m7GpppX diphosphatase
402	Macrophage migration inhibitory factor	Macrophage migration inhibitory factor	Macrophage migration inhibitory factor	Macrophage migration inhibitory factor	Macrophage migration inhibitory factor	Macrophage migration inhibitory factor
403	Macrophage-capping protein	Macrophage-capping protein	Macrophage-capping protein	Macrophage-capping protein	Macrophage-capping protein	Macrophage-capping protein
404	Major vault protein	Major vault protein	Major vault protein	Major vault protein	Major vault protein	Major vault protein
405	Malate dehydrogenase, cytoplasmic	Malate dehydrogenase, cytoplasmic	Malate dehydrogenase, cytoplasmic	Malate dehydrogenase, cytoplasmic	Malate dehydrogenase, cytoplasmic	Malate dehydrogenase, cytoplasmic
406	Malate dehydrogenase, mitochondrial	Malate dehydrogenase, mitochondrial	Malate dehydrogenase, mitochondrial	Malate dehydrogenase, mitochondrial	Malate dehydrogenase, mitochondrial	Malate dehydrogenase, mitochondrial
407	Maltase-glucoamylase	Maltase-glucoamylase	Maltase-glucoamylase	Maltase-glucoamylase	Maltase-glucoamylase	Maltase-glucoamylase
408	Matrix metalloproteinase-25		Matrix metalloproteinase-25	Matrix metalloproteinase-25	Matrix metalloproteinase-25	Matrix metalloproteinase-25
409	Matrix metalloproteinase-9	Matrix metalloproteinase-9	Matrix metalloproteinase-9	Matrix metalloproteinase-9	Matrix metalloproteinase-9	Matrix metalloproteinase-9
410	Metalloproteinase inhibitor 1	Metalloproteinase inhibitor 1	Metalloproteinase inhibitor 1	Metalloproteinase inhibitor 1	Metalloproteinase inhibitor 1	Metalloproteinase inhibitor 1
411	Metalloproteinase inhibitor 2	Metalloproteinase inhibitor 2	Metalloproteinase inhibitor 2	Metalloproteinase inhibitor 2	Metalloproteinase inhibitor 2	Metalloproteinase inhibitor 2
412	Microtubule-associated protein RP/EB family member 1	Microtubule-associated protein RP/EB family member 1		Microtubule-associated protein RP/EB family member 1	Microtubule-associated protein RP/EB family member 1	Microtubule-associated protein RP/EB family member 1
413	Mitochondrial peptide methionine sulfoxide reductase	Mitochondrial peptide methionine sulfoxide reductase	Mitochondrial peptide methionine sulfoxide reductase		Mitochondrial peptide methionine sulfoxide reductase	Mitochondrial peptide methionine sulfoxide reductase
414				Mitogen-activated protein kinase 1	Mitogen-activated protein kinase 1	Mitogen-activated protein kinase 1

415	Mitogen-activated protein kinase 14			Mitogen-activated protein kinase 14	Mitogen-activated protein kinase 14	Mitogen-activated protein kinase 14
416	Moesin	Moesin	Moesin	Moesin	Moesin	Moesin
417	Monocyte differentiation antigen CD14	Monocyte differentiation antigen CD14	Monocyte differentiation antigen CD14	Monocyte differentiation antigen CD14	Monocyte differentiation antigen CD14	Monocyte differentiation antigen CD14
418	Mucin-5B	Mucin-5B	Mucin-5B	Mucin-5B	Mucin-5B	Mucin-5B
419						Multimerin-1
420	Myeloblastin	Myeloblastin	Myeloblastin	Myeloblastin	Myeloblastin	Myeloblastin
421	Myeloid cell nuclear differentiation antigen	Myeloid cell nuclear differentiation antigen	Myeloid cell nuclear differentiation antigen	Myeloid cell nuclear differentiation antigen	Myeloid cell nuclear differentiation antigen	Myeloid cell nuclear differentiation antigen
422	Myeloid cell surface antigen CD33	Myeloid cell surface antigen CD33	Myeloid cell surface antigen CD33	Myeloid cell surface antigen CD33	Myeloid cell surface antigen CD33	Myeloid cell surface antigen CD33
423	Myeloid-derived growth factor	Myeloid-derived growth factor	Myeloid-derived growth factor	Myeloid-derived growth factor	Myeloid-derived growth factor	Myeloid-derived growth factor
424	Myeloperoxidase	Myeloperoxidase	Myeloperoxidase	Myeloperoxidase	Myeloperoxidase	Myeloperoxidase
425	Myosin light polypeptide 6	Myosin light polypeptide 6	Myosin light polypeptide 6	Myosin light polypeptide 6	Myosin light polypeptide 6	Myosin light polypeptide 6
426	Myosin regulatory light chain 12B	Myosin regulatory light chain 12B	Myosin regulatory light chain 12B	Myosin regulatory light chain 12B	Myosin regulatory light chain 12B	Myosin regulatory light chain 12B
427	Myosin-8	Myosin-8	Myosin-8	Myosin-8	Myosin-8	Myosin-8
428	Myosin-9	Myosin-9	Myosin-9	Myosin-9	Myosin-9	Myosin-9
429	Myotrophin	Myotrophin	Myotrophin	Myotrophin	Myotrophin	Myotrophin
430	Myristoylated alanine-rich C-kinase substrate	Myristoylated alanine-rich C-kinase substrate	Myristoylated alanine-rich C-kinase substrate	Myristoylated alanine-rich C-kinase substrate	Myristoylated alanine-rich C-kinase substrate	Myristoylated alanine-rich C-kinase substrate
431		N-acetyl-D-glucosamine kinase		N-acetyl-D-glucosamine kinase	N-acetyl-D-glucosamine kinase	N-acetyl-D-glucosamine kinase
432	N-acetylglucosamine-6-sulfatase	N-acetylglucosamine-6-sulfatase	N-acetylglucosamine-6-sulfatase	N-acetylglucosamine-6-sulfatase	N-acetylglucosamine-6-sulfatase	N-acetylglucosamine-6-sulfatase
433		N(G),N(G)-dimethylarginine dimethylaminohydrolase 2	N(G),N(G)-dimethylarginine dimethylaminohydrolase 2		N(G),N(G)-dimethylarginine dimethylaminohydrolase 2	
434	Na(+)/H(+) exchange regulatory cofactor NHE-RF1	Na(+)/H(+) exchange regulatory cofactor NHE-RF1	Na(+)/H(+) exchange regulatory cofactor NHE-RF1	Na(+)/H(+) exchange regulatory cofactor NHE-RF1	Na(+)/H(+) exchange regulatory cofactor NHE-RF1	Na(+)/H(+) exchange regulatory cofactor NHE-RF1
435	NAD kinase	NAD kinase		NAD kinase	NAD kinase	NAD kinase
436				NEDD8	NEDD8	
437	Neutral alpha-glucosidase AB	Neutral alpha-glucosidase AB	Neutral alpha-glucosidase AB	Neutral alpha-glucosidase AB	Neutral alpha-glucosidase AB	Neutral alpha-glucosidase AB
438	Neutrophil collagenase	Neutrophil collagenase	Neutrophil collagenase	Neutrophil collagenase	Neutrophil collagenase	Neutrophil collagenase
439	Neutrophil cytosol factor 1	Neutrophil cytosol factor 1	Neutrophil cytosol factor 1	Neutrophil cytosol factor 1	Neutrophil cytosol factor 1	Neutrophil cytosol factor 1
440	Neutrophil cytosol factor 2	Neutrophil cytosol factor 2	Neutrophil cytosol factor 2	Neutrophil cytosol factor 2	Neutrophil cytosol factor 2	Neutrophil cytosol factor 2
441	Neutrophil cytosol factor 4	Neutrophil cytosol factor 4	Neutrophil cytosol factor 4	Neutrophil cytosol factor 4	Neutrophil cytosol factor 4	Neutrophil cytosol factor 4
442	Neutrophil defensin 1	Neutrophil defensin 1	Neutrophil defensin 1	Neutrophil defensin 1	Neutrophil defensin 1	Neutrophil defensin 1
443	Neutrophil defensin 3		Neutrophil defensin 3	Neutrophil defensin 3		Neutrophil defensin 3
444	Neutrophil elastase	Neutrophil elastase	Neutrophil elastase	Neutrophil elastase	Neutrophil elastase	Neutrophil elastase
445	Neutrophil gelatinase-associated lipocalin	Neutrophil gelatinase-associated lipocalin	Neutrophil gelatinase-associated lipocalin	Neutrophil gelatinase-associated lipocalin	Neutrophil gelatinase-associated lipocalin	Neutrophil gelatinase-associated lipocalin
446	Nicotinamide phosphoribosyltransferase	Nicotinamide phosphoribosyltransferase	Nicotinamide phosphoribosyltransferase	Nicotinamide phosphoribosyltransferase	Nicotinamide phosphoribosyltransferase	Nicotinamide phosphoribosyltransferase

447	Nicotinate phosphoribosyltransferase	Nicotinate phosphoribosyltransferase	Nicotinate phosphoribosyltransferase	Nicotinate phosphoribosyltransferase	Nicotinate phosphoribosyltransferase	Nicotinate phosphoribosyltransferase
448	Non-histone chromosomal protein HMG-14	Non-histone chromosomal protein HMG-14	Non-histone chromosomal protein HMG-14	Non-histone chromosomal protein HMG-14	Non-histone chromosomal protein HMG-14	Non-histone chromosomal protein HMG-14
449	Non-histone chromosomal protein HMG-17	Non-histone chromosomal protein HMG-17	Non-histone chromosomal protein HMG-17	Non-histone chromosomal protein HMG-17	Non-histone chromosomal protein HMG-17	Non-histone chromosomal protein HMG-17
450		Non-POU domain-containing octamer-binding protein	Non-POU domain-containing octamer-binding protein	Non-POU domain-containing octamer-binding protein	Non-POU domain-containing octamer-binding protein	Non-POU domain-containing octamer-binding protein
451	Non-secretory ribonuclease	Non-secretory ribonuclease	Non-secretory ribonuclease	Non-secretory ribonuclease	Non-secretory ribonuclease	Non-secretory ribonuclease
452	NPC intracellular cholesterol transporter 2	NPC intracellular cholesterol transporter 2	NPC intracellular cholesterol transporter 2	NPC intracellular cholesterol transporter 2	NPC intracellular cholesterol transporter 2	NPC intracellular cholesterol transporter 2
453	Nuclear transport factor 2	Nuclear transport factor 2	Nuclear transport factor 2	Nuclear transport factor 2	Nuclear transport factor 2	Nuclear transport factor 2
454	Nuclear ubiquitous casein and cyclin-dependent kinase substrate 1		Nuclear ubiquitous casein and cyclin-dependent kinase substrate 1	Nuclear ubiquitous casein and cyclin-dependent kinase substrate 1		Nuclear ubiquitous casein and cyclin-dependent kinase substrate 1
455		Nucleolin		Nucleolin	Nucleolin	
456	Nucleophosmin	Nucleophosmin	Nucleophosmin	Nucleophosmin	Nucleophosmin	Nucleophosmin
457	Nucleoside diphosphate kinase B	Nucleoside diphosphate kinase B	Nucleoside diphosphate kinase B	Nucleoside diphosphate kinase B	Nucleoside diphosphate kinase B	Nucleoside diphosphate kinase B
458		Obg-like ATPase 1			Obg-like ATPase 1	
459	Olfactomedin-4	Olfactomedin-4	Olfactomedin-4	Olfactomedin-4	Olfactomedin-4	Olfactomedin-4
460	Omega-amidase NIT2	Omega-amidase NIT2	Omega-amidase NIT2	Omega-amidase NIT2	Omega-amidase NIT2	Omega-amidase NIT2
461	Osteoclast-stimulating factor 1	Osteoclast-stimulating factor 1	Osteoclast-stimulating factor 1	Osteoclast-stimulating factor 1	Osteoclast-stimulating factor 1	Osteoclast-stimulating factor 1
462	Oxidized low-density lipoprotein receptor 1	Oxidized low-density lipoprotein receptor 1	Oxidized low-density lipoprotein receptor 1	Oxidized low-density lipoprotein receptor 1	Oxidized low-density lipoprotein receptor 1	Oxidized low-density lipoprotein receptor 1
463	Pantetheinase	Pantetheinase	Pantetheinase		Pantetheinase	Pantetheinase
464	Pantetheine hydrolase VNN2	Pantetheine hydrolase VNN2		Pantetheine hydrolase VNN2	Pantetheine hydrolase VNN2	Pantetheine hydrolase VNN2
465	Parathymosin	Parathymosin	Parathymosin	Parathymosin	Parathymosin	Parathymosin
466	Parkinson disease protein 7	Parkinson disease protein 7	Parkinson disease protein 7	Parkinson disease protein 7	Parkinson disease protein 7	Parkinson disease protein 7
467	Pentraxin-related protein PTX3	Pentraxin-related protein PTX3	Pentraxin-related protein PTX3	Pentraxin-related protein PTX3	Pentraxin-related protein PTX3	Pentraxin-related protein PTX3
468	Peptidoglycan recognition protein 1	Peptidoglycan recognition protein 1	Peptidoglycan recognition protein 1	Peptidoglycan recognition protein 1	Peptidoglycan recognition protein 1	Peptidoglycan recognition protein 1
469	Peptidyl-prolyl cis-trans isomerase A	Peptidyl-prolyl cis-trans isomerase A	Peptidyl-prolyl cis-trans isomerase A	Peptidyl-prolyl cis-trans isomerase A	Peptidyl-prolyl cis-trans isomerase A	Peptidyl-prolyl cis-trans isomerase A
470	Peptidyl-prolyl cis-trans isomerase B	Peptidyl-prolyl cis-trans isomerase B	Peptidyl-prolyl cis-trans isomerase B	Peptidyl-prolyl cis-trans isomerase B	Peptidyl-prolyl cis-trans isomerase B	Peptidyl-prolyl cis-trans isomerase B
471	Peptidyl-prolyl cis-trans isomerase FKBP1A	Peptidyl-prolyl cis-trans isomerase FKBP1A	Peptidyl-prolyl cis-trans isomerase FKBP1A	Peptidyl-prolyl cis-trans isomerase FKBP1A	Peptidyl-prolyl cis-trans isomerase FKBP1A	Peptidyl-prolyl cis-trans isomerase FKBP1A
472	Perilipin-3	Perilipin-3	Perilipin-3	Perilipin-3	Perilipin-3	Perilipin-3
473	Peroxiredoxin-1	Peroxiredoxin-1	Peroxiredoxin-1	Peroxiredoxin-1	Peroxiredoxin-1	Peroxiredoxin-1
474	Peroxiredoxin-2	Peroxiredoxin-2	Peroxiredoxin-2	Peroxiredoxin-2	Peroxiredoxin-2	Peroxiredoxin-2
475	Peroxiredoxin-5, mitochondrial	Peroxiredoxin-5, mitochondrial	Peroxiredoxin-5, mitochondrial	Peroxiredoxin-5, mitochondrial	Peroxiredoxin-5, mitochondrial	Peroxiredoxin-5, mitochondrial
476	Peroxiredoxin-6	Peroxiredoxin-6	Peroxiredoxin-6	Peroxiredoxin-6	Peroxiredoxin-6	Peroxiredoxin-6
477	PEST proteolytic signal-containing nuclear protein	PEST proteolytic signal-containing nuclear protein	PEST proteolytic signal-containing nuclear protein	PEST proteolytic signal-containing nuclear protein	PEST proteolytic signal-containing nuclear protein	PEST proteolytic signal-containing nuclear protein
478	Phosphatidylethanolamine-binding protein 1	Phosphatidylethanolamine-binding protein 1	Phosphatidylethanolamine-binding protein 1	Phosphatidylethanolamine-binding protein 1	Phosphatidylethanolamine-binding protein 1	Phosphatidylethanolamine-binding protein 1

479	Phosphatidylinositol 3,4,5-trisphosphate 5-phosphatase 1	Phosphatidylinositol 3,4,5-trisphosphate 5-phosphatase 1	Phosphatidylinositol 3,4,5-trisphosphate 5-phosphatase 1	Phosphatidylinositol 3,4,5-trisphosphate 5-phosphatase 1	Phosphatidylinositol 3,4,5-trisphosphate 5-phosphatase 1	Phosphatidylinositol 3,4,5-trisphosphate 5-phosphatase 1
480	Phosphatidylinositol 5-phosphate 4-kinase type-2 alpha	Phosphatidylinositol 5-phosphate 4-kinase type-2 alpha	Phosphatidylinositol 5-phosphate 4-kinase type-2 alpha	Phosphatidylinositol 5-phosphate 4-kinase type-2 alpha	Phosphatidylinositol 5-phosphate 4-kinase type-2 alpha	Phosphatidylinositol 5-phosphate 4-kinase type-2 alpha
481			Phosphatidylinositol-binding clathrin assembly protein	Phosphatidylinositol-binding clathrin assembly protein		
482	Phosphoglucomutase-1	Phosphoglucomutase-1	Phosphoglucomutase-1	Phosphoglucomutase-1	Phosphoglucomutase-1	Phosphoglucomutase-1
483	Phosphoglycerate kinase 1	Phosphoglycerate kinase 1	Phosphoglycerate kinase 1	Phosphoglycerate kinase 1	Phosphoglycerate kinase 1	Phosphoglycerate kinase 1
484	Phosphoglycerate mutase 1	Phosphoglycerate mutase 1	Phosphoglycerate mutase 1	Phosphoglycerate mutase 1	Phosphoglycerate mutase 1	Phosphoglycerate mutase 1
485	Phospholipase B-like 1	Phospholipase B-like 1	Phospholipase B-like 1	Phospholipase B-like 1	Phospholipase B-like 1	Phospholipase B-like 1
486	Phosphopentomutase	Phosphopentomutase	Phosphopentomutase	Phosphopentomutase	Phosphopentomutase	Phosphopentomutase
487	PI-PLC X domain-containing protein 1	PI-PLC X domain-containing protein 1	PI-PLC X domain-containing protein 1	PI-PLC X domain-containing protein 1	PI-PLC X domain-containing protein 1	PI-PLC X domain-containing protein 1
488	Plasminogen	Plasminogen	Plasminogen			Plasminogen
489	Plastin-2	Plastin-2	Plastin-2	Plastin-2	Plastin-2	Plastin-2
490	Platelet basic protein	Platelet basic protein	Platelet basic protein	Platelet basic protein	Platelet basic protein	Platelet basic protein
491	Platelet endothelial cell adhesion molecule	Platelet endothelial cell adhesion molecule	Platelet endothelial cell adhesion molecule	Platelet endothelial cell adhesion molecule	Platelet endothelial cell adhesion molecule	Platelet endothelial cell adhesion molecule
492	Platelet factor 4	Platelet factor 4	Platelet factor 4	Platelet factor 4	Platelet factor 4	Platelet factor 4
493	Platelet-activating factor acetylhydrolase IB subunit beta	Platelet-activating factor acetylhydrolase IB subunit beta	Platelet-activating factor acetylhydrolase IB subunit beta	Platelet-activating factor acetylhydrolase IB subunit beta	Platelet-activating factor acetylhydrolase IB subunit beta	Platelet-activating factor acetylhydrolase IB subunit beta
494	Plectin	Plectin		Plectin	Plectin	Plectin
495	Plexin-C1	Plexin-C1	Plexin-C1	Plexin-C1	Plexin-C1	Plexin-C1
496	PML-RARA-regulated adapter molecule 1	PML-RARA-regulated adapter molecule 1	PML-RARA-regulated adapter molecule 1	PML-RARA-regulated adapter molecule 1	PML-RARA-regulated adapter molecule 1	PML-RARA-regulated adapter molecule 1
497	Poly(rC)-binding protein 1	Poly(rC)-binding protein 1	Poly(rC)-binding protein 1	Poly(rC)-binding protein 1	Poly(rC)-binding protein 1	Poly(rC)-binding protein 1
498	Polymeric immunoglobulin receptor	Polymeric immunoglobulin receptor		Polymeric immunoglobulin receptor		Polymeric immunoglobulin receptor
499		Polypyrimidine tract-binding protein 1		Polypyrimidine tract-binding protein 1	Polypyrimidine tract-binding protein 1	Polypyrimidine tract-binding protein 1
500	Polyunsaturated fatty acid lipoxygenase ALOX15	Polyunsaturated fatty acid lipoxygenase ALOX15	Polyunsaturated fatty acid lipoxygenase ALOX15	Polyunsaturated fatty acid lipoxygenase ALOX15	Polyunsaturated fatty acid lipoxygenase ALOX15	Polyunsaturated fatty acid lipoxygenase ALOX15
501				Pre-mRNA-processing factor 19	Pre-mRNA-processing factor 19	Pre-mRNA-processing factor 19
502		Prefoldin subunit 2	Prefoldin subunit 2	Prefoldin subunit 2	Prefoldin subunit 2	Prefoldin subunit 2
503	Profilin-1	Profilin-1	Profilin-1	Profilin-1	Profilin-1	Profilin-1
504	Programmed cell death 6-interacting protein	Programmed cell death 6-interacting protein	Programmed cell death 6-interacting protein	Programmed cell death 6-interacting protein	Programmed cell death 6-interacting protein	Programmed cell death 6-interacting protein
505	Programmed cell death protein 5	Programmed cell death protein 5	Programmed cell death protein 5	Programmed cell death protein 5	Programmed cell death protein 5	Programmed cell death protein 5
506	Progranulin	Progranulin	Progranulin	Progranulin	Progranulin	Progranulin
507	Prolactin-inducible protein	Prolactin-inducible protein				
508	Proliferation-associated protein 2G4	Proliferation-associated protein 2G4	Proliferation-associated protein 2G4	Proliferation-associated protein 2G4	Proliferation-associated protein 2G4	Proliferation-associated protein 2G4
509			Proline-serine-threonine phosphatase-interacting protein 2	Proline-serine-threonine phosphatase-interacting protein 2	Proline-serine-threonine phosphatase-interacting protein 2	Proline-serine-threonine phosphatase-interacting protein 2
510	Properdin	Properdin	Properdin	Properdin	Properdin	Properdin

511	Prosaposin	Prosaposin	Prosaposin	Prosaposin	Prosaposin	Prosaposin
512	Proteasome activator complex subunit 1	Proteasome activator complex subunit 1	Proteasome activator complex subunit 1	Proteasome activator complex subunit 1	Proteasome activator complex subunit 1	Proteasome activator complex subunit 1
513		Proteasome activator complex subunit 2		Proteasome activator complex subunit 2	Proteasome activator complex subunit 2	Proteasome activator complex subunit 2
514	Proteasome subunit alpha type-1	Proteasome subunit alpha type-1	Proteasome subunit alpha type-1	Proteasome subunit alpha type-1	Proteasome subunit alpha type-1	Proteasome subunit alpha type-1
515	Proteasome subunit alpha type-2	Proteasome subunit alpha type-2	Proteasome subunit alpha type-2	Proteasome subunit alpha type-2	Proteasome subunit alpha type-2	Proteasome subunit alpha type-2
516	Proteasome subunit alpha type-3	Proteasome subunit alpha type-3	Proteasome subunit alpha type-3	Proteasome subunit alpha type-3	Proteasome subunit alpha type-3	Proteasome subunit alpha type-3
517	Proteasome subunit alpha type-5	Proteasome subunit alpha type-5	Proteasome subunit alpha type-5	Proteasome subunit alpha type-5	Proteasome subunit alpha type-5	Proteasome subunit alpha type-5
518	Proteasome subunit alpha type-6	Proteasome subunit alpha type-6	Proteasome subunit alpha type-6	Proteasome subunit alpha type-6	Proteasome subunit alpha type-6	Proteasome subunit alpha type-6
519	Proteasome subunit alpha type-7	Proteasome subunit alpha type-7	Proteasome subunit alpha type-7	Proteasome subunit alpha type-7	Proteasome subunit alpha type-7	Proteasome subunit alpha type-7
520	Proteasome subunit beta type-1	Proteasome subunit beta type-1	Proteasome subunit beta type-1	Proteasome subunit beta type-1	Proteasome subunit beta type-1	Proteasome subunit beta type-1
521	Proteasome subunit beta type-10	Proteasome subunit beta type-10	Proteasome subunit beta type-10	Proteasome subunit beta type-10	Proteasome subunit beta type-10	Proteasome subunit beta type-10
522	Proteasome subunit beta type-2	Proteasome subunit beta type-2	Proteasome subunit beta type-2	Proteasome subunit beta type-2	Proteasome subunit beta type-2	Proteasome subunit beta type-2
523	Proteasome subunit beta type-3	Proteasome subunit beta type-3	Proteasome subunit beta type-3	Proteasome subunit beta type-3	Proteasome subunit beta type-3	Proteasome subunit beta type-3
524		Proteasome subunit beta type-4		Proteasome subunit beta type-4	Proteasome subunit beta type-4	
525		Proteasome subunit beta type-6	Proteasome subunit beta type-6	Proteasome subunit beta type-6	Proteasome subunit beta type-6	Proteasome subunit beta type-6
526		Proteasome subunit beta type-7	Proteasome subunit beta type-7	Proteasome subunit beta type-7	Proteasome subunit beta type-7	
527	Proteasome subunit beta type-8	Proteasome subunit beta type-8	Proteasome subunit beta type-8	Proteasome subunit beta type-8	Proteasome subunit beta type-8	Proteasome subunit beta type-8
528	Proteasome subunit beta type-9	Proteasome subunit beta type-9	Proteasome subunit beta type-9	Proteasome subunit beta type-9	Proteasome subunit beta type-9	Proteasome subunit beta type-9
529	Protein CDV3 homolog	Protein CDV3 homolog	Protein CDV3 homolog	Protein CDV3 homolog	Protein CDV3 homolog	Protein CDV3 homolog
530	Protein diaphanous homolog 1	Protein diaphanous homolog 1	Protein diaphanous homolog 1	Protein diaphanous homolog 1	Protein diaphanous homolog 1	Protein diaphanous homolog 1
531	Protein disulfide-isomerase	Protein disulfide-isomerase	Protein disulfide-isomerase	Protein disulfide-isomerase	Protein disulfide-isomerase	Protein disulfide-isomerase
532	Protein disulfide-isomerase A3	Protein disulfide-isomerase A3	Protein disulfide-isomerase A3	Protein disulfide-isomerase A3	Protein disulfide-isomerase A3	Protein disulfide-isomerase A3
533	Protein FAM107B	Protein FAM107B	Protein FAM107B	Protein FAM107B	Protein FAM107B	Protein FAM107B
534		Protein flightless-1 homolog		Protein flightless-1 homolog	Protein flightless-1 homolog	Protein flightless-1 homolog
535	Protein kinase C delta type	Protein kinase C delta type	Protein kinase C delta type	Protein kinase C delta type		Protein kinase C delta type
536	Protein Niban 1	Protein Niban 1	Protein Niban 1	Protein Niban 1	Protein Niban 1	Protein Niban 1
537						Protein phosphatase 1 regulatory subunit 12A
538	Protein RCC2		Protein RCC2	Protein RCC2	Protein RCC2	Protein RCC2
539	Protein S100-A11	Protein S100-A11	Protein S100-A11	Protein S100-A11	Protein S100-A11	Protein S100-A11
540	Protein S100-A12	Protein S100-A12	Protein S100-A12	Protein S100-A12	Protein S100-A12	Protein S100-A12
541	Protein S100-A4	Protein S100-A4	Protein S100-A4	Protein S100-A4	Protein S100-A4	Protein S100-A4
542	Protein S100-A6	Protein S100-A6	Protein S100-A6	Protein S100-A6	Protein S100-A6	Protein S100-A6

543	Protein S100-A8	Protein S100-A8	Protein S100-A8	Protein S100-A8	Protein S100-A8	Protein S100-A8
544	Protein S100-A9	Protein S100-A9	Protein S100-A9	Protein S100-A9	Protein S100-A9	Protein S100-A9
545	Protein S100-P	Protein S100-P	Protein S100-P	Protein S100-P	Protein S100-P	Protein S100-P
546	Protein SET	Protein SET			Protein SET	Protein SET
547	Protein unc-13 homolog D	Protein unc-13 homolog D	Protein unc-13 homolog D	Protein unc-13 homolog D	Protein unc-13 homolog D	Protein unc-13 homolog D
548		Protein XRP2	Protein XRP2	Protein XRP2	Protein XRP2	Protein XRP2
549	Protein-arginine deiminase type-2	Protein-arginine deiminase type-2	Protein-arginine deiminase type-2	Protein-arginine deiminase type-2	Protein-arginine deiminase type-2	Protein-arginine deiminase type-2
550	Protein-arginine deiminase type-4	Protein-arginine deiminase type-4	Protein-arginine deiminase type-4	Protein-arginine deiminase type-4	Protein-arginine deiminase type-4	Protein-arginine deiminase type-4
551	Protein-L-isoaspartate(D-aspartate) O-methyltransferase	Protein-L-isoaspartate(D-aspartate) O-methyltransferase	Protein-L-isoaspartate(D-aspartate) O-methyltransferase	Protein-L-isoaspartate(D-aspartate) O-methyltransferase	Protein-L-isoaspartate(D-aspartate) O-methyltransferase	Protein-L-isoaspartate(D-aspartate) O-methyltransferase
552		Proteoglycan 3	Proteoglycan 3	Proteoglycan 3	Proteoglycan 3	Proteoglycan 3
553	Prothymosin alpha	Prothymosin alpha	Prothymosin alpha	Prothymosin alpha	Prothymosin alpha	Prothymosin alpha
554	Purine nucleoside phosphorylase	Purine nucleoside phosphorylase	Purine nucleoside phosphorylase	Purine nucleoside phosphorylase	Purine nucleoside phosphorylase	Purine nucleoside phosphorylase
555		Puromycin-sensitive aminopeptidase	Puromycin-sensitive aminopeptidase	Puromycin-sensitive aminopeptidase	Puromycin-sensitive aminopeptidase	Puromycin-sensitive aminopeptidase
556		Putative ubiquitin-conjugating enzyme E2 N-like		Putative ubiquitin-conjugating enzyme E2 N-like	Putative ubiquitin-conjugating enzyme E2 N-like	Putative ubiquitin-conjugating enzyme E2 N-like
557	Pyridoxal kinase	Pyridoxal kinase	Pyridoxal kinase	Pyridoxal kinase	Pyridoxal kinase	Pyridoxal kinase
558			Pyridoxal phosphate homeostasis protein	Pyridoxal phosphate homeostasis protein	Pyridoxal phosphate homeostasis protein	Pyridoxal phosphate homeostasis protein
559	Pyruvate kinase PKM	Pyruvate kinase PKM	Pyruvate kinase PKM	Pyruvate kinase PKM	Pyruvate kinase PKM	Pyruvate kinase PKM
560	Rab GDP dissociation inhibitor alpha	Rab GDP dissociation inhibitor alpha	Rab GDP dissociation inhibitor alpha	Rab GDP dissociation inhibitor alpha	Rab GDP dissociation inhibitor alpha	Rab GDP dissociation inhibitor alpha
561	Rab GDP dissociation inhibitor beta	Rab GDP dissociation inhibitor beta	Rab GDP dissociation inhibitor beta	Rab GDP dissociation inhibitor beta	Rab GDP dissociation inhibitor beta	Rab GDP dissociation inhibitor beta
562	Rab GTPase-activating protein 1-like, isoform 10	Rab GTPase-activating protein 1-like, isoform 10	Rab GTPase-activating protein 1-like, isoform 10	Rab GTPase-activating protein 1-like, isoform 10	Rab GTPase-activating protein 1-like, isoform 10	Rab GTPase-activating protein 1-like, isoform 10
563	Ras GTPase-activating-like protein IQGAP1	Ras GTPase-activating-like protein IQGAP1	Ras GTPase-activating-like protein IQGAP1	Ras GTPase-activating-like protein IQGAP1	Ras GTPase-activating-like protein IQGAP1	Ras GTPase-activating-like protein IQGAP1
564						
565	Ras-related C3 botulinum toxin substrate 2	Ras-related C3 botulinum toxin substrate 2	Ras-related C3 botulinum toxin substrate 2	Ras-related C3 botulinum toxin substrate 2	Ras-related C3 botulinum toxin substrate 2	Ras-related C3 botulinum toxin substrate 2
566	Ras-related protein Rab-10		Ras-related protein Rab-10	Ras-related protein Rab-10	Ras-related protein Rab-10	Ras-related protein Rab-10
567	Ras-related protein Rab-11A	Ras-related protein Rab-11A	Ras-related protein Rab-11A	Ras-related protein Rab-11A	Ras-related protein Rab-11A	Ras-related protein Rab-11A
568				Ras-related protein Rab-14		
569	Ras-related protein Rab-1A	Ras-related protein Rab-1A	Ras-related protein Rab-1A	Ras-related protein Rab-1A	Ras-related protein Rab-1A	Ras-related protein Rab-1A
570	Ras-related protein Rab-1B	Ras-related protein Rab-1B	Ras-related protein Rab-1B	Ras-related protein Rab-1B	Ras-related protein Rab-1B	Ras-related protein Rab-1B
571				Ras-related protein Rab-21	Ras-related protein Rab-21	Ras-related protein Rab-21
572	Ras-related protein Rab-27A	Ras-related protein Rab-27A	Ras-related protein Rab-27A	Ras-related protein Rab-27A	Ras-related protein Rab-27A	Ras-related protein Rab-27A
573	Ras-related protein Rab-2A	Ras-related protein Rab-2A	Ras-related protein Rab-2A	Ras-related protein Rab-2A	Ras-related protein Rab-2A	Ras-related protein Rab-2A
574		Ras-related protein Rab-3D	Ras-related protein Rab-3D	Ras-related protein Rab-3D	Ras-related protein Rab-3D	Ras-related protein Rab-3D

575						
576	Ras-related protein Rab-5B	Ras-related protein Rab-5B	Ras-related protein Rab-5B	Ras-related protein Rab-5B	Ras-related protein Rab-5B	Ras-related protein Rab-5B
577	Ras-related protein Rab-5C	Ras-related protein Rab-5C		Ras-related protein Rab-5C		Ras-related protein Rab-5C
578		Ras-related protein Rab-6A	Ras-related protein Rab-6A	Ras-related protein Rab-6A	Ras-related protein Rab-6A	Ras-related protein Rab-6A
579		Ras-related protein Rab-7a	Ras-related protein Rab-7a	Ras-related protein Rab-7a	Ras-related protein Rab-7a	Ras-related protein Rab-7a
580	Ras-related protein Rab-8A			Ras-related protein Rab-8A	Ras-related protein Rab-8A	Ras-related protein Rab-8A
581				Ras-related protein Rab-8B	Ras-related protein Rab-8B	Ras-related protein Rab-8B
582		Ras-related protein Ral-B	Ras-related protein Ral-B	Ras-related protein Ral-B	Ras-related protein Ral-B	Ras-related protein Ral-B
583	Ras-related protein Rap-1b	Ras-related protein Rap-1b	Ras-related protein Rap-1b	Ras-related protein Rap-1b	Ras-related protein Rap-1b	Ras-related protein Rap-1b
584	Receptor-type tyrosine-protein phosphatase C	Receptor-type tyrosine-protein phosphatase C	Receptor-type tyrosine-protein phosphatase C	Receptor-type tyrosine-protein phosphatase C	Receptor-type tyrosine-protein phosphatase C	Receptor-type tyrosine-protein phosphatase C
585	Receptor-type tyrosine-protein phosphatase eta	Receptor-type tyrosine-protein phosphatase eta	Receptor-type tyrosine-protein phosphatase eta	Receptor-type tyrosine-protein phosphatase eta	Receptor-type tyrosine-protein phosphatase eta	Receptor-type tyrosine-protein phosphatase eta
586	Resistin	Resistin	Resistin	Resistin	Resistin	Resistin
587	Rho GDP-dissociation inhibitor 1	Rho GDP-dissociation inhibitor 1	Rho GDP-dissociation inhibitor 1	Rho GDP-dissociation inhibitor 1	Rho GDP-dissociation inhibitor 1	Rho GDP-dissociation inhibitor 1
588	Rho GDP-dissociation inhibitor 2	Rho GDP-dissociation inhibitor 2	Rho GDP-dissociation inhibitor 2	Rho GDP-dissociation inhibitor 2	Rho GDP-dissociation inhibitor 2	Rho GDP-dissociation inhibitor 2
589	Rho GTPase-activating protein 25	Rho GTPase-activating protein 25	Rho GTPase-activating protein 25	Rho GTPase-activating protein 25	Rho GTPase-activating protein 25	Rho GTPase-activating protein 25
590	Rho GTPase-activating protein 45	Rho GTPase-activating protein 45	Rho GTPase-activating protein 45	Rho GTPase-activating protein 45	Rho GTPase-activating protein 45	Rho GTPase-activating protein 45
591		Rho guanine nucleotide exchange factor 1	Rho guanine nucleotide exchange factor 1	Rho guanine nucleotide exchange factor 1	Rho guanine nucleotide exchange factor 1	Rho guanine nucleotide exchange factor 1
592	Rho-associated protein kinase 1	Rho-associated protein kinase 1	Rho-associated protein kinase 1	Rho-associated protein kinase 1	Rho-associated protein kinase 1	
593	Rho-related GTP-binding protein RhoG	Rho-related GTP-binding protein RhoG	Rho-related GTP-binding protein RhoG	Rho-related GTP-binding protein RhoG	Rho-related GTP-binding protein RhoG	Rho-related GTP-binding protein RhoG
594		Ribonuclease inhibitor	Ribonuclease inhibitor	Ribonuclease inhibitor	Ribonuclease inhibitor	Ribonuclease inhibitor
595	Ribonuclease T2	Ribonuclease T2	Ribonuclease T2	Ribonuclease T2	Ribonuclease T2	Ribonuclease T2
596	Ribose-5-phosphate isomerase	Ribose-5-phosphate isomerase	Ribose-5-phosphate isomerase	Ribose-5-phosphate isomerase	Ribose-5-phosphate isomerase	Ribose-5-phosphate isomerase
597		Ribosyldihydronicotinamide dehydrogenase [quinone]	Ribosyldihydronicotinamide dehydrogenase [quinone]	Ribosyldihydronicotinamide dehydrogenase [quinone]	Ribosyldihydronicotinamide dehydrogenase [quinone]	Ribosyldihydronicotinamide dehydrogenase [quinone]
598	Ribulose-phosphate 3-epimerase	Ribulose-phosphate 3-epimerase	Ribulose-phosphate 3-epimerase	Ribulose-phosphate 3-epimerase	Ribulose-phosphate 3-epimerase	Ribulose-phosphate 3-epimerase
599	RNA-binding protein 8A	RNA-binding protein 8A	RNA-binding protein 8A	RNA-binding protein 8A	RNA-binding protein 8A	RNA-binding protein 8A
600	RNA-binding protein Raly		RNA-binding protein Raly	RNA-binding protein Raly	RNA-binding protein Raly	RNA-binding protein Raly
601				S-formylglutathione hydrolase	S-formylglutathione hydrolase	S-formylglutathione hydrolase
602	S-methyl-5'-thioadenosine phosphorylase	S-methyl-5'-thioadenosine phosphorylase	S-methyl-5'-thioadenosine phosphorylase	S-methyl-5'-thioadenosine phosphorylase	S-methyl-5'-thioadenosine phosphorylase	S-methyl-5'-thioadenosine phosphorylase
603				SAM and SH3 domain-containing protein 3		
604	Sarcoplasmic/endoplasmic reticulum calcium ATPase 3	Sarcoplasmic/endoplasmic reticulum calcium ATPase 3	Sarcoplasmic/endoplasmic reticulum calcium ATPase 3	Sarcoplasmic/endoplasmic reticulum calcium ATPase 3	Sarcoplasmic/endoplasmic reticulum calcium ATPase 3	Sarcoplasmic/endoplasmic reticulum calcium ATPase 3
605	Semaphorin-4A	Semaphorin-4A			Semaphorin-4A	
606						

607		Septin-2		Septin-2		Septin-2
608	Septin-6	Septin-6	Septin-6	Septin-6	Septin-6	Septin-6
609	Septin-7	Septin-7	Septin-7	Septin-7	Septin-7	Septin-7
610		Septin-9	Septin-9	Septin-9	Septin-9	Septin-9
611	Serglycin	Serglycin	Serglycin	Serglycin	Serglycin	
612	Serine protease 57	Serine protease 57	Serine protease 57	Serine protease 57	Serine protease 57	Serine protease 57
613	Serine--tRNA ligase, cytoplasmic	Serine--tRNA ligase, cytoplasmic		Serine--tRNA ligase, cytoplasmic	Serine--tRNA ligase, cytoplasmic	Serine--tRNA ligase, cytoplasmic
614	Serine/arginine-rich splicing factor 2	Serine/arginine-rich splicing factor 2	Serine/arginine-rich splicing factor 2	Serine/arginine-rich splicing factor 2	Serine/arginine-rich splicing factor 2	Serine/arginine-rich splicing factor 2
615	Serine/arginine-rich splicing factor 3	Serine/arginine-rich splicing factor 3	Serine/arginine-rich splicing factor 3	Serine/arginine-rich splicing factor 3	Serine/arginine-rich splicing factor 3	Serine/arginine-rich splicing factor 3
616		Serine/arginine-rich splicing factor 4		Serine/arginine-rich splicing factor 4		
617	Serine/threonine-protein kinase 10	Serine/threonine-protein kinase 10	Serine/threonine-protein kinase 10	Serine/threonine-protein kinase 10	Serine/threonine-protein kinase 10	Serine/threonine-protein kinase 10
618	Serine/threonine-protein kinase 4	Serine/threonine-protein kinase 4	Serine/threonine-protein kinase 4	Serine/threonine-protein kinase 4	Serine/threonine-protein kinase 4	Serine/threonine-protein kinase 4
619			Serine/threonine-protein kinase Nek9	Serine/threonine-protein kinase Nek9		Serine/threonine-protein kinase Nek9
620		Serine/threonine-protein kinase OSR1	Serine/threonine-protein kinase OSR1	Serine/threonine-protein kinase OSR1	Serine/threonine-protein kinase OSR1	Serine/threonine-protein kinase OSR1
621	Serine/threonine-protein kinase PAK 2	Serine/threonine-protein kinase PAK 2	Serine/threonine-protein kinase PAK 2	Serine/threonine-protein kinase PAK 2	Serine/threonine-protein kinase PAK 2	Serine/threonine-protein kinase PAK 2
622	Serine/threonine-protein kinase WNK1					
623	Serine/threonine-protein phosphatase 2A 65 kDa regulatory subunit A alpha isoform	Serine/threonine-protein phosphatase 2A 65 kDa regulatory subunit A alpha isoform	Serine/threonine-protein phosphatase 2A 65 kDa regulatory subunit A alpha isoform	Serine/threonine-protein phosphatase 2A 65 kDa regulatory subunit A alpha isoform	Serine/threonine-protein phosphatase 2A 65 kDa regulatory subunit A alpha isoform	Serine/threonine-protein phosphatase 2A 65 kDa regulatory subunit A alpha isoform
624	Serine/threonine-protein phosphatase 2A activator	Serine/threonine-protein phosphatase 2A activator	Serine/threonine-protein phosphatase 2A activator	Serine/threonine-protein phosphatase 2A activator	Serine/threonine-protein phosphatase 2A activator	Serine/threonine-protein phosphatase 2A activator
625	Serine/threonine-protein phosphatase 6 catalytic subunit	Serine/threonine-protein phosphatase 6 catalytic subunit	Serine/threonine-protein phosphatase 6 catalytic subunit	Serine/threonine-protein phosphatase 6 catalytic subunit	Serine/threonine-protein phosphatase 6 catalytic subunit	Serine/threonine-protein phosphatase 6 catalytic subunit
626					Serine/threonine-protein phosphatase 6 regulatory ankyrin repeat subunit B	
627	Serine/threonine-protein phosphatase CPPED1	Serine/threonine-protein phosphatase CPPED1	Serine/threonine-protein phosphatase CPPED1	Serine/threonine-protein phosphatase CPPED1	Serine/threonine-protein phosphatase CPPED1	Serine/threonine-protein phosphatase CPPED1
628	Serine/threonine-protein phosphatase PP1-alpha catalytic subunit	Serine/threonine-protein phosphatase PP1-alpha catalytic subunit	Serine/threonine-protein phosphatase PP1-alpha catalytic subunit	Serine/threonine-protein phosphatase PP1-alpha catalytic subunit	Serine/threonine-protein phosphatase PP1-alpha catalytic subunit	Serine/threonine-protein phosphatase PP1-alpha catalytic subunit
629	Serotransferrin	Serotransferrin	Serotransferrin	Serotransferrin	Serotransferrin	Serotransferrin
630	Serpin B10	Serpin B10	Serpin B10	Serpin B10	Serpin B10	Serpin B10
631	SERPINE1 mRNA-binding protein 1	SERPINE1 mRNA-binding protein 1	SERPINE1 mRNA-binding protein 1	SERPINE1 mRNA-binding protein 1	SERPINE1 mRNA-binding protein 1	SERPINE1 mRNA-binding protein 1
632						
633	SH3 domain-binding glutamic acid-rich-like protein 3	SH3 domain-binding glutamic acid-rich-like protein 3	SH3 domain-binding glutamic acid-rich-like protein 3	SH3 domain-binding glutamic acid-rich-like protein 3	SH3 domain-binding glutamic acid-rich-like protein 3	SH3 domain-binding glutamic acid-rich-like protein 3
634	SH3 domain-containing kinase-binding protein 1	SH3 domain-containing kinase-binding protein 1	SH3 domain-containing kinase-binding protein 1	SH3 domain-containing kinase-binding protein 1	SH3 domain-containing kinase-binding protein 1	SH3 domain-containing kinase-binding protein 1
635	Sialic acid-binding Ig-like lectin 14	Sialic acid-binding Ig-like lectin 14	Sialic acid-binding Ig-like lectin 14	Sialic acid-binding Ig-like lectin 14	Sialic acid-binding Ig-like lectin 14	Sialic acid-binding Ig-like lectin 14
636	Sialic acid-binding Ig-like lectin 5	Sialic acid-binding Ig-like lectin 5	Sialic acid-binding Ig-like lectin 5	Sialic acid-binding Ig-like lectin 5	Sialic acid-binding Ig-like lectin 5	Sialic acid-binding Ig-like lectin 5
637	Sialic acid-binding Ig-like lectin 9	Sialic acid-binding Ig-like lectin 9	Sialic acid-binding Ig-like lectin 9	Sialic acid-binding Ig-like lectin 9	Sialic acid-binding Ig-like lectin 9	Sialic acid-binding Ig-like lectin 9
638	Signal recognition particle 14 kDa protein	Signal recognition particle 14 kDa protein	Signal recognition particle 14 kDa protein	Signal recognition particle 14 kDa protein	Signal recognition particle 14 kDa protein	Signal recognition particle 14 kDa protein

639	Signal recognition particle 9 kDa protein	Signal recognition particle 9 kDa protein	Signal recognition particle 9 kDa protein	Signal recognition particle 9 kDa protein	Signal recognition particle 9 kDa protein	Signal recognition particle 9 kDa protein
640		Signal-induced proliferation-associated protein 1	Signal-induced proliferation-associated protein 1	Signal-induced proliferation-associated protein 1	Signal-induced proliferation-associated protein 1	Signal-induced proliferation-associated protein 1
641	Signal-regulatory protein beta-1	Signal-regulatory protein beta-1	Signal-regulatory protein beta-1	Signal-regulatory protein beta-1	Signal-regulatory protein beta-1	Signal-regulatory protein beta-1
642	Small nuclear ribonucleoprotein E	Small nuclear ribonucleoprotein E	Small nuclear ribonucleoprotein E	Small nuclear ribonucleoprotein E	Small nuclear ribonucleoprotein E	Small nuclear ribonucleoprotein E
643	Small nuclear ribonucleoprotein F			Small nuclear ribonucleoprotein F	Small nuclear ribonucleoprotein F	Small nuclear ribonucleoprotein F
644				Small nuclear ribonucleoprotein Sm D1		
645		Small nuclear ribonucleoprotein Sm D2	Small nuclear ribonucleoprotein Sm D2	Small nuclear ribonucleoprotein Sm D2	Small nuclear ribonucleoprotein Sm D2	Small nuclear ribonucleoprotein Sm D2
646	Small nuclear ribonucleoprotein Sm D3	Small nuclear ribonucleoprotein Sm D3	Small nuclear ribonucleoprotein Sm D3	Small nuclear ribonucleoprotein Sm D3	Small nuclear ribonucleoprotein Sm D3	Small nuclear ribonucleoprotein Sm D3
647	Small nuclear ribonucleoprotein-associated proteins B and B'	Small nuclear ribonucleoprotein-associated proteins B and B'	Small nuclear ribonucleoprotein-associated proteins B and B'	Small nuclear ribonucleoprotein-associated proteins B and B'	Small nuclear ribonucleoprotein-associated proteins B and B'	Small nuclear ribonucleoprotein-associated proteins B and B'
648	Small ribosomal subunit protein eS27	Small ribosomal subunit protein eS27	Small ribosomal subunit protein eS27	Small ribosomal subunit protein eS27	Small ribosomal subunit protein eS27	Small ribosomal subunit protein eS27
649	Small ribosomal subunit protein eS6	Small ribosomal subunit protein eS6	Small ribosomal subunit protein eS6	Small ribosomal subunit protein eS6	Small ribosomal subunit protein eS6	Small ribosomal subunit protein eS6
650		Small ribosomal subunit protein uS11	Small ribosomal subunit protein uS11	Small ribosomal subunit protein uS11	Small ribosomal subunit protein uS11	
651		Small ribosomal subunit protein uS13				
652		Small ribosomal subunit protein uS2	Small ribosomal subunit protein uS2	Small ribosomal subunit protein uS2	Small ribosomal subunit protein uS2	Small ribosomal subunit protein uS2
653	Small ubiquitin-related modifier 2	Small ubiquitin-related modifier 2	Small ubiquitin-related modifier 2	Small ubiquitin-related modifier 2	Small ubiquitin-related modifier 2	Small ubiquitin-related modifier 2
654						
655	Solute carrier family 2, facilitated glucose transporter member 3	Solute carrier family 2, facilitated glucose transporter member 3	Solute carrier family 2, facilitated glucose transporter member 3	Solute carrier family 2, facilitated glucose transporter member 3	Solute carrier family 2, facilitated glucose transporter member 3	Solute carrier family 2, facilitated glucose transporter member 3
656	Spectrin alpha chain, erythrocytic 1	Spectrin alpha chain, erythrocytic 1	Spectrin alpha chain, erythrocytic 1	Spectrin alpha chain, erythrocytic 1	Spectrin alpha chain, erythrocytic 1	Spectrin alpha chain, erythrocytic 1
657	Spectrin alpha chain, non-erythrocytic 1	Spectrin alpha chain, non-erythrocytic 1	Spectrin alpha chain, non-erythrocytic 1	Spectrin alpha chain, non-erythrocytic 1	Spectrin alpha chain, non-erythrocytic 1	Spectrin alpha chain, non-erythrocytic 1
658	Spectrin beta chain, erythrocytic	Spectrin beta chain, erythrocytic	Spectrin beta chain, erythrocytic	Spectrin beta chain, erythrocytic	Spectrin beta chain, erythrocytic	Spectrin beta chain, erythrocytic
659	Spectrin beta chain, non-erythrocytic 1	Spectrin beta chain, non-erythrocytic 1	Spectrin beta chain, non-erythrocytic 1	Spectrin beta chain, non-erythrocytic 1	Spectrin beta chain, non-erythrocytic 1	Spectrin beta chain, non-erythrocytic 1
660	Spliceosome RNA helicase DDX39B	Spliceosome RNA helicase DDX39B	Spliceosome RNA helicase DDX39B	Spliceosome RNA helicase DDX39B	Spliceosome RNA helicase DDX39B	Spliceosome RNA helicase DDX39B
661	Splicing factor, proline- and glutamine-rich	Splicing factor, proline- and glutamine-rich	Splicing factor, proline- and glutamine-rich	Splicing factor, proline- and glutamine-rich	Splicing factor, proline- and glutamine-rich	Splicing factor, proline- and glutamine-rich
662		Src kinase-associated phosphoprotein 2	Src kinase-associated phosphoprotein 2	Src kinase-associated phosphoprotein 2	Src kinase-associated phosphoprotein 2	Src kinase-associated phosphoprotein 2
663	Stathmin	Stathmin	Stathmin	Stathmin	Stathmin	Stathmin
664	Stomatin	Stomatin	Stomatin	Stomatin	Stomatin	Stomatin
665		Stress-induced-phosphoprotein 1	Stress-induced-phosphoprotein 1	Stress-induced-phosphoprotein 1	Stress-induced-phosphoprotein 1	Stress-induced-phosphoprotein 1
666				Stromal membrane-associated protein 2		
667				Structural maintenance of chromosomes protein 1A	Structural maintenance of chromosomes protein 1A	Structural maintenance of chromosomes protein 1A
668			Structural maintenance of chromosomes protein 3	Structural maintenance of chromosomes protein 3	Structural maintenance of chromosomes protein 3	Structural maintenance of chromosomes protein 3
669	Sulfhydryl oxidase 1	Sulfhydryl oxidase 1	Sulfhydryl oxidase 1	Sulfhydryl oxidase 1	Sulfhydryl oxidase 1	Sulfhydryl oxidase 1
670		SUMO-conjugating enzyme UBC9	SUMO-conjugating enzyme UBC9	SUMO-conjugating enzyme UBC9		

671	Superoxide dismutase [Cu-Zn]	Superoxide dismutase [Cu-Zn]	Superoxide dismutase [Cu-Zn]	Superoxide dismutase [Cu-Zn]	Superoxide dismutase [Cu-Zn]	Superoxide dismutase [Cu-Zn]
672	Supervillin	Supervillin	Supervillin	Supervillin	Supervillin	Supervillin
673				SWI/SNF complex subunit SMARCC2		SWI/SNF complex subunit SMARCC2
674	Synaptic vesicle membrane protein VAT-1 homolog	Synaptic vesicle membrane protein VAT-1 homolog	Synaptic vesicle membrane protein VAT-1 homolog	Synaptic vesicle membrane protein VAT-1 homolog	Synaptic vesicle membrane protein VAT-1 homolog	Synaptic vesicle membrane protein VAT-1 homolog
675				Syntaxin-3		
676	Syntaxin-7	Syntaxin-7	Syntaxin-7	Syntaxin-7	Syntaxin-7	Syntaxin-7
677		Syntaxin-binding protein 2	Syntaxin-binding protein 2	Syntaxin-binding protein 2		
678	Syntenin-1	Syntenin-1	Syntenin-1	Syntenin-1	Syntenin-1	Syntenin-1
679	T-complex protein 1 subunit alpha	T-complex protein 1 subunit alpha		T-complex protein 1 subunit alpha	T-complex protein 1 subunit alpha	
680	T-complex protein 1 subunit beta			T-complex protein 1 subunit beta	T-complex protein 1 subunit beta	T-complex protein 1 subunit beta
681	T-complex protein 1 subunit delta	T-complex protein 1 subunit delta	T-complex protein 1 subunit delta	T-complex protein 1 subunit delta	T-complex protein 1 subunit delta	T-complex protein 1 subunit delta
682	T-complex protein 1 subunit gamma	T-complex protein 1 subunit gamma	T-complex protein 1 subunit gamma	T-complex protein 1 subunit gamma	T-complex protein 1 subunit gamma	T-complex protein 1 subunit gamma
683	T-complex protein 1 subunit theta	T-complex protein 1 subunit theta		T-complex protein 1 subunit theta	T-complex protein 1 subunit theta	T-complex protein 1 subunit theta
684	Talin-1	Talin-1	Talin-1	Talin-1	Talin-1	Talin-1
685	Thioredoxin	Thioredoxin	Thioredoxin	Thioredoxin	Thioredoxin	Thioredoxin
686	Thioredoxin domain-containing protein 17	Thioredoxin domain-containing protein 17	Thioredoxin domain-containing protein 17	Thioredoxin domain-containing protein 17	Thioredoxin domain-containing protein 17	Thioredoxin domain-containing protein 17
687			Thioredoxin reductase 1, cytoplasmic	Thioredoxin reductase 1, cytoplasmic	Thioredoxin reductase 1, cytoplasmic	Thioredoxin reductase 1, cytoplasmic
688		Thioredoxin-dependent peroxide reductase, mitochondrial	Thioredoxin-dependent peroxide reductase, mitochondrial	Thioredoxin-dependent peroxide reductase, mitochondrial	Thioredoxin-dependent peroxide reductase, mitochondrial	Thioredoxin-dependent peroxide reductase, mitochondrial
689		Thioredoxin-like protein 1			Thioredoxin-like protein 1	
690	THO complex subunit 4	THO complex subunit 4	THO complex subunit 4	THO complex subunit 4	THO complex subunit 4	THO complex subunit 4
691	Thrombospondin-1	Thrombospondin-1	Thrombospondin-1	Thrombospondin-1	Thrombospondin-1	Thrombospondin-1
692	Thymidine phosphorylase	Thymidine phosphorylase	Thymidine phosphorylase	Thymidine phosphorylase	Thymidine phosphorylase	Thymidine phosphorylase
693	Thymocyte nuclear protein 1	Thymocyte nuclear protein 1	Thymocyte nuclear protein 1	Thymocyte nuclear protein 1	Thymocyte nuclear protein 1	Thymocyte nuclear protein 1
694	Thymosin beta-10	Thymosin beta-10	Thymosin beta-10	Thymosin beta-10	Thymosin beta-10	Thymosin beta-10
695	Thymosin beta-4	Thymosin beta-4	Thymosin beta-4	Thymosin beta-4	Thymosin beta-4	Thymosin beta-4
696	Thyroid hormone receptor-associated protein 3	Thyroid hormone receptor-associated protein 3	Thyroid hormone receptor-associated protein 3	Thyroid hormone receptor-associated protein 3	Thyroid hormone receptor-associated protein 3	Thyroid hormone receptor-associated protein 3
697	Transaldolase	Transaldolase	Transaldolase	Transaldolase	Transaldolase	Transaldolase
698	Transcobalamin-1	Transcobalamin-1	Transcobalamin-1	Transcobalamin-1	Transcobalamin-1	Transcobalamin-1
699		Transcription elongation factor A protein-like 3		Transcription elongation factor A protein-like 3	Transcription elongation factor A protein-like 3	Transcription elongation factor A protein-like 3
700			Transforming acidic coiled-coil-containing protein 3	Transforming acidic coiled-coil-containing protein 3		
701	Transforming protein RhoA	Transforming protein RhoA	Transforming protein RhoA	Transforming protein RhoA	Transforming protein RhoA	Transforming protein RhoA
702	Transgelin-2	Transgelin-2	Transgelin-2	Transgelin-2	Transgelin-2	Transgelin-2

703	Transitional endoplasmic reticulum ATPase	Transitional endoplasmic reticulum ATPase	Transitional endoplasmic reticulum ATPase	Transitional endoplasmic reticulum ATPase	Transitional endoplasmic reticulum ATPase	Transitional endoplasmic reticulum ATPase
704	Transketolase	Transketolase	Transketolase	Transketolase	Transketolase	Transketolase
705	Translation machinery-associated protein 7	Translation machinery-associated protein 7	Translation machinery-associated protein 7	Translation machinery-associated protein 7	Translation machinery-associated protein 7	Translation machinery-associated protein 7
706				Translationally-controlled tumor protein	Translationally-controlled tumor protein	Translationally-controlled tumor protein
707	Translin	Translin	Translin	Translin	Translin	Translin
708	Translin-associated protein X	Translin-associated protein X	Translin-associated protein X	Translin-associated protein X	Translin-associated protein X	Translin-associated protein X
709			Triokinase/FMN cyclase	Triokinase/FMN cyclase	Triokinase/FMN cyclase	Triokinase/FMN cyclase
710	Triosephosphate isomerase	Triosephosphate isomerase	Triosephosphate isomerase	Triosephosphate isomerase	Triosephosphate isomerase	Triosephosphate isomerase
711		Tropomodulin-3	Tropomodulin-3	Tropomodulin-3	Tropomodulin-3	Tropomodulin-3
712	Tropomyosin alpha-3 chain	Tropomyosin alpha-3 chain	Tropomyosin alpha-3 chain	Tropomyosin alpha-3 chain	Tropomyosin alpha-3 chain	Tropomyosin alpha-3 chain
713	Tropomyosin alpha-4 chain	Tropomyosin alpha-4 chain	Tropomyosin alpha-4 chain	Tropomyosin alpha-4 chain	Tropomyosin alpha-4 chain	Tropomyosin alpha-4 chain
714	Tryptophan--tRNA ligase, cytoplasmic	Tryptophan--tRNA ligase, cytoplasmic	Tryptophan--tRNA ligase, cytoplasmic	Tryptophan--tRNA ligase, cytoplasmic	Tryptophan--tRNA ligase, cytoplasmic	Tryptophan--tRNA ligase, cytoplasmic
715	Tubulin alpha-1B chain	Tubulin alpha-1B chain	Tubulin alpha-1B chain	Tubulin alpha-1B chain	Tubulin alpha-1B chain	Tubulin alpha-1B chain
716	Tubulin beta chain		Tubulin beta chain	Tubulin beta chain	Tubulin beta chain	
717	Tubulin beta-1 chain	Tubulin beta-1 chain	Tubulin beta-1 chain	Tubulin beta-1 chain		
718	Tubulin-specific chaperone A	Tubulin-specific chaperone A	Tubulin-specific chaperone A	Tubulin-specific chaperone A	Tubulin-specific chaperone A	Tubulin-specific chaperone A
719	Tumor necrosis factor receptor superfamily member 10C	Tumor necrosis factor receptor superfamily member 10C	Tumor necrosis factor receptor superfamily member 10C	Tumor necrosis factor receptor superfamily member 10C	Tumor necrosis factor receptor superfamily member 10C	Tumor necrosis factor receptor superfamily member 10C
720						
721	Tumor protein D54	Tumor protein D54	Tumor protein D54	Tumor protein D54	Tumor protein D54	Tumor protein D54
722	Tumor susceptibility gene 101 protein	Tumor susceptibility gene 101 protein	Tumor susceptibility gene 101 protein		Tumor susceptibility gene 101 protein	Tumor susceptibility gene 101 protein
723	Twinfilin-2	Twinfilin-2		Twinfilin-2	Twinfilin-2	Twinfilin-2
724			Tyrosine-protein kinase CSK	Tyrosine-protein kinase CSK	Tyrosine-protein kinase CSK	Tyrosine-protein kinase CSK
725	Tyrosine-protein kinase Fgr	Tyrosine-protein kinase Fgr	Tyrosine-protein kinase Fgr	Tyrosine-protein kinase Fgr	Tyrosine-protein kinase Fgr	Tyrosine-protein kinase Fgr
726	Tyrosine-protein kinase HCK	Tyrosine-protein kinase HCK	Tyrosine-protein kinase HCK	Tyrosine-protein kinase HCK	Tyrosine-protein kinase HCK	Tyrosine-protein kinase HCK
727	Tyrosine-protein kinase Lyn		Tyrosine-protein kinase Lyn	Tyrosine-protein kinase Lyn		Tyrosine-protein kinase Lyn
728		Tyrosine-protein phosphatase non-receptor type 12	Tyrosine-protein phosphatase non-receptor type 12	Tyrosine-protein phosphatase non-receptor type 12	Tyrosine-protein phosphatase non-receptor type 12	
729		Tyrosine-protein phosphatase non-receptor type 18		Tyrosine-protein phosphatase non-receptor type 18	Tyrosine-protein phosphatase non-receptor type 18	Tyrosine-protein phosphatase non-receptor type 18
730	Tyrosine-protein phosphatase non-receptor type 6	Tyrosine-protein phosphatase non-receptor type 6	Tyrosine-protein phosphatase non-receptor type 6	Tyrosine-protein phosphatase non-receptor type 6	Tyrosine-protein phosphatase non-receptor type 6	Tyrosine-protein phosphatase non-receptor type 6
731			Tyrosine-protein phosphatase non-receptor type substrate 1		Tyrosine-protein phosphatase non-receptor type substrate 1	Tyrosine-protein phosphatase non-receptor type substrate 1
732	U6 snRNA-associated Sm-like protein LSm2		U6 snRNA-associated Sm-like protein LSm2	U6 snRNA-associated Sm-like protein LSm2	U6 snRNA-associated Sm-like protein LSm2	U6 snRNA-associated Sm-like protein LSm2
733	U6 snRNA-associated Sm-like protein LSm3	U6 snRNA-associated Sm-like protein LSm3	U6 snRNA-associated Sm-like protein LSm3	U6 snRNA-associated Sm-like protein LSm3	U6 snRNA-associated Sm-like protein LSm3	U6 snRNA-associated Sm-like protein LSm3
734		U6 snRNA-associated Sm-like protein LSm4	U6 snRNA-associated Sm-like protein LSm4	U6 snRNA-associated Sm-like protein LSm4	U6 snRNA-associated Sm-like protein LSm4	U6 snRNA-associated Sm-like protein LSm4

735	U6 snRNA-associated Sm-like protein LSm8	U6 snRNA-associated Sm-like protein LSm8	U6 snRNA-associated Sm-like protein LSm8	U6 snRNA-associated Sm-like protein LSm8	U6 snRNA-associated Sm-like protein LSm8	U6 snRNA-associated Sm-like protein LSm8
736	Ubiquitin carboxyl-terminal hydrolase 14			Ubiquitin carboxyl-terminal hydrolase 14		
737	Ubiquitin carboxyl-terminal hydrolase 5		Ubiquitin carboxyl-terminal hydrolase 5	Ubiquitin carboxyl-terminal hydrolase 5	Ubiquitin carboxyl-terminal hydrolase 5	Ubiquitin carboxyl-terminal hydrolase 5
738			Ubiquitin recognition factor in ER-associated degradation protein 1	Ubiquitin recognition factor in ER-associated degradation protein 1	Ubiquitin recognition factor in ER-associated degradation protein 1	Ubiquitin recognition factor in ER-associated degradation protein 1
739	Ubiquitin-conjugating enzyme E2 L3	Ubiquitin-conjugating enzyme E2 L3	Ubiquitin-conjugating enzyme E2 L3	Ubiquitin-conjugating enzyme E2 L3	Ubiquitin-conjugating enzyme E2 L3	Ubiquitin-conjugating enzyme E2 L3
740	Ubiquitin-conjugating enzyme E2 variant 2	Ubiquitin-conjugating enzyme E2 variant 2	Ubiquitin-conjugating enzyme E2 variant 2	Ubiquitin-conjugating enzyme E2 variant 2	Ubiquitin-conjugating enzyme E2 variant 2	Ubiquitin-conjugating enzyme E2 variant 2
741		Ubiquitin-fold modifier-conjugating enzyme 1	Ubiquitin-fold modifier-conjugating enzyme 1	Ubiquitin-fold modifier-conjugating enzyme 1	Ubiquitin-fold modifier-conjugating enzyme 1	Ubiquitin-fold modifier-conjugating enzyme 1
742	Ubiquitin-like modifier-activating enzyme 1	Ubiquitin-like modifier-activating enzyme 1	Ubiquitin-like modifier-activating enzyme 1	Ubiquitin-like modifier-activating enzyme 1	Ubiquitin-like modifier-activating enzyme 1	Ubiquitin-like modifier-activating enzyme 1
743	Ubiquitin-ribosomal protein eL40 fusion protein	Ubiquitin-ribosomal protein eL40 fusion protein	Ubiquitin-ribosomal protein eL40 fusion protein	Ubiquitin-ribosomal protein eL40 fusion protein	Ubiquitin-ribosomal protein eL40 fusion protein	Ubiquitin-ribosomal protein eL40 fusion protein
744		UMP-CMP kinase	UMP-CMP kinase	UMP-CMP kinase	UMP-CMP kinase	UMP-CMP kinase
745	Unconventional myosin-If	Unconventional myosin-If	Unconventional myosin-If	Unconventional myosin-If	Unconventional myosin-If	Unconventional myosin-If
746	Unconventional myosin-XVIIIa	Unconventional myosin-XVIIIa	Unconventional myosin-XVIIIa	Unconventional myosin-XVIIIa	Unconventional myosin-XVIIIa	Unconventional myosin-XVIIIa
747	Urokinase plasminogen activator surface receptor	Urokinase plasminogen activator surface receptor	Urokinase plasminogen activator surface receptor	Urokinase plasminogen activator surface receptor	Urokinase plasminogen activator surface receptor	Urokinase plasminogen activator surface receptor
748	Urokinase-type plasminogen activator	Urokinase-type plasminogen activator	Urokinase-type plasminogen activator	Urokinase-type plasminogen activator	Urokinase-type plasminogen activator	Urokinase-type plasminogen activator
749	UTP--glucose-1-phosphate uridylyltransferase	UTP--glucose-1-phosphate uridylyltransferase	UTP--glucose-1-phosphate uridylyltransferase	UTP--glucose-1-phosphate uridylyltransferase	UTP--glucose-1-phosphate uridylyltransferase	UTP--glucose-1-phosphate uridylyltransferase
750		V-set and transmembrane domain-containing protein 1	V-set and transmembrane domain-containing protein 1	V-set and transmembrane domain-containing protein 1	V-set and transmembrane domain-containing protein 1	V-set and transmembrane domain-containing protein 1
751	V-type proton ATPase catalytic subunit A		V-type proton ATPase catalytic subunit A	V-type proton ATPase catalytic subunit A	V-type proton ATPase catalytic subunit A	V-type proton ATPase catalytic subunit A
752				V-type proton ATPase subunit E 1		V-type proton ATPase subunit E 1
753	V-type proton ATPase subunit G 1	V-type proton ATPase subunit G 1	V-type proton ATPase subunit G 1	V-type proton ATPase subunit G 1	V-type proton ATPase subunit G 1	V-type proton ATPase subunit G 1
754	Vacuolar protein sorting-associated protein 35		Vacuolar protein sorting-associated protein 35	Vacuolar protein sorting-associated protein 35	Vacuolar protein sorting-associated protein 35	Vacuolar protein sorting-associated protein 35
755				Vacuolar protein sorting-associated protein 4B		Vacuolar protein sorting-associated protein 4B
756	Vasodilator-stimulated phosphoprotein	Vasodilator-stimulated phosphoprotein	Vasodilator-stimulated phosphoprotein	Vasodilator-stimulated phosphoprotein	Vasodilator-stimulated phosphoprotein	Vasodilator-stimulated phosphoprotein
757	Vesicle-associated membrane protein 8	Vesicle-associated membrane protein 8	Vesicle-associated membrane protein 8	Vesicle-associated membrane protein 8	Vesicle-associated membrane protein 8	Vesicle-associated membrane protein 8
758		Vesicle-trafficking protein SEC22b		Vesicle-trafficking protein SEC22b	Vesicle-trafficking protein SEC22b	Vesicle-trafficking protein SEC22b
759	Vimentin	Vimentin	Vimentin	Vimentin	Vimentin	Vimentin
760	Vinculin	Vinculin	Vinculin	Vinculin	Vinculin	Vinculin
761	Vitamin D-binding protein	Vitamin D-binding protein	Vitamin D-binding protein	Vitamin D-binding protein	Vitamin D-binding protein	Vitamin D-binding protein
762	von Willebrand factor	von Willebrand factor	von Willebrand factor	von Willebrand factor		von Willebrand factor
763	WAS/WASL-interacting protein family member 1	WAS/WASL-interacting protein family member 1	WAS/WASL-interacting protein family member 1	WAS/WASL-interacting protein family member 1	WAS/WASL-interacting protein family member 1	WAS/WASL-interacting protein family member 1
764		WASH complex subunit 2C			WASH complex subunit 2C	WASH complex subunit 2C
765	WD repeat-containing protein 1	WD repeat-containing protein 1	WD repeat-containing protein 1	WD repeat-containing protein 1	WD repeat-containing protein 1	WD repeat-containing protein 1
766		X-ray repair cross-complementing protein 6	X-ray repair cross-complementing protein 6	X-ray repair cross-complementing protein 6	X-ray repair cross-complementing protein 6	X-ray repair cross-complementing protein 6

767	Y-box-binding protein 1	Y-box-binding protein 1	Y-box-binding protein 1	Y-box-binding protein 1	Y-box-binding protein 1	Y-box-binding protein 1
768	Zyxin	Zyxin	Zyxin	Zyxin	Zyxin	Zyxin

Table S1. Table of proteins identified by nLC-MS/MS for three neutrophil donors in control and after stimulation with AC 264613. Proteins presented in the table were filtered to include only proteins identified by 1% FDR and at least two unique peptides.

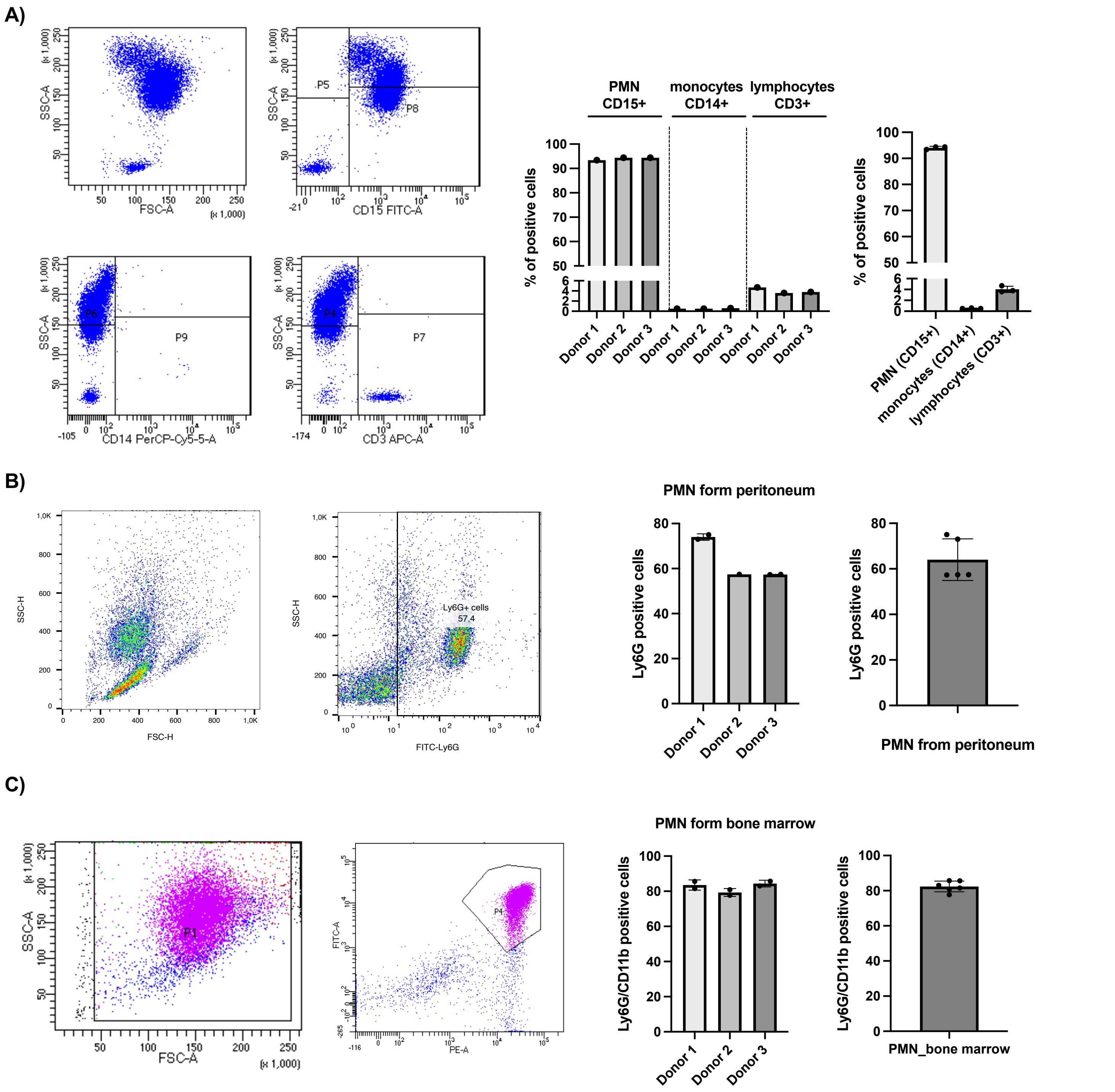

Fig. S5. Purity of PMN isolated from human blood and murine neutrophils isolated from peritoneum and bone marrow determined by flow cytometry. (A) The purity of human PMN fraction was determined using cell specific antibodies anti-human CD15 (neutrophils), anti-human CD14 (monocytes), anti-human CD3 (lymphocytes). (B) Purity of peritoneal neutrophils was determined using anti-mouse Ly6G-FITC antibodies. (C) Purity of bone marrow neutrophils was determined using anti-mouse Ly6G-FITC and CD11b-PE antibodies. Results were obtained from 3 donors and shown as mean ± SEM.

Gene	Sequence	Program[1]
EF-2 F	5' GAC ATC ACC AAG GGT GTG CAG 3'	
EF-2 R	5' TTC AGC ACA CTG GCA TAG AGG C 3'	
F2R F	5' CTG TGG TGT ATC CCA TGC AG 3'	Denaturation:
F2R R	5' GCC AGA CAA GTG AAG GAA GC 3'	95 °C, 30 s
F2RL1 F	5' TCC TCA CTG GAA AAC TGA CC 3'	Annealing:
F2RL1 R	5' GGA AAA GAA AGA CCC ACA GG 3'	57 °C, 30 s
F2RL2 F	5' GGT GTG GGC AAC AGT TTT CT 3'	Elongation:
F2RL2 R	5' GGA CTC GCA AGT GTT GTG AA 3'	72 °C, 45 s
F2RL3 F	5' ACC ATG CTG CTG ATG AAC CT 3'	
F2RL3 R	5' AGC ACT GAG CCA TAC ATG TGA C 3'	

Table S2. Primer sequences and amplification conditions for qRT-PCR.
