# Supplementary material for: Non-classical neutrophil extracellular traps induced by PAR2-signaling proteases
Source: Cell Death Dis. 2025 Feb 19;16(1):109. doi: 10.1038/s41419-025-07428-z (PMC11840154; doi:10.1038/s41419-025-07428-z)
Supplement: Supplementary file 2 — Original Data [file 41419_2025_7428_MOESM2_ESM.pdf]

Fig. 1. NET formation is triggered by PAR2-activating proteases and small-molecule PAR2 agonists but not by proteases that cleave PAR2 at non-canonical sites.

Fig. 1B   Relative expression levels of *PAR1* (*F2R*), *PAR2* (*F2RL1*), *PAR3* (*F2RL3*) and *PAR4* (*F2RL3*) mRNA in human neutrophils.

F2R	*F2RL1*	*F2RL2*	*F2RL3*
0,0508622	0,5509096	0,1685493	0,0007663
0,0474517	0,5428984	0,1803241	0,0008149
0,02656	0,7998517	0,3074414	0,0086097
0,0387842	0,9282687	0,2623602	0,0093386
0,0333667	0,7580668	0,3156702	0,0056284
0,0308096	0,7887091	0,2657898	0,0050767

Fig. 1C   The amount of extracellular DNA released by neutrophils 3 h post-incubation with trypsin, kallikrein 14 (KLK14), cathepsin G (catG) and neutrophil elastase (NE) at the indicated concentrations (μM) based on SytoxGreen staining (RFU = relative fluorescence units).

-	0.025	0.05	0.25	0.025	0.05	0.25	0.05	0.25	0.1	0.2
38,336	77,935	137,6	359,65	63,355	93,731	109,321	48,566	27,709	12,085	33,683
34,642	27,179	167,38	174,36	109,345	69,883	145,999	44,542	31,789	24,444	38,578
39,489	112,03	237,51	203,16	76,755	64,827	94,552	27,232	31,669	13,727	18,11
31,114	108,78	154,32	274,65	57,784		90,091	15,735	38,505	56,594	48,606
54,108	92,478	104,96	125,93	63,945		183,101667	19,706	37,875	49,961	52,921
41,013				46,611		160,863332	30,795	32,554	59,115	43,021

Fig. 1D   The amount of extracellular DNA released by neutrophils 3 h post-incubation with 0.05 μM trypsin, trypsin pre-incubated in a 1:1 ratio with aprotinin based on SytoxGreen staining.

-	trypsin	trypsin + aprotinin	aprotinin
23,266	154,32	22,687	40,125
38,486	104,96	21,201	21,089
39,638	137,6	21,591	22,331
43,129	167,38	40,786	14,295
30,57	237,51	13,892	14,944
23,578		37,858	19,212

Fig. 1E   The amount of extracellular DNA released by neutrophils 3 h post-stimulation with trypsin (0.05 μM) after pre-incubation with 100 μM FSLLRY-NH_2 for 10 min.

-	FSLLRY-NH_2	trypsin	FSLLRY-NH_2+ trypsin
39,062	73,343	90,862	84,525
28,991	26,042	171,948	66,335
70,099	56,387	149,72	84,699
62,929	53,46	138,816	99,529
72,456	48,373	90,662	60,86
66,225	11,691	118,816	44,896
51,867	14,741	100,951	38,676
53,632	59,554	125,338	75,1
45,462	104,289	159,825	90,6
59,884	58,784	140,823	90,252

Fig. 1F   The amount of extracellular DNA released by neutrophils 3 h post-stimulation with 100 μM AC-264613 or SLIGRL-NH_2 (PAR2 agonists) or LRGILS-NH_2 (reversed amino acid sequence control peptide for SLIGRL-NH_2) based on SytoxGreen staining.

-	AC 264613	SLIGRL-NH_2	LRGILS-NH_2
79,42	114,829	78,4	27,104
84,994	100,788	129,5	26,548
81,075	109,92	113,5	27,092
35,166	95,039		69,106
51,8	100,681		74,058
36,933	146,92		77,326
59,91	111,386		51,549
64,522	171,884		72,116
65,56	170,903		28,235
30,717	128,706		54,4
29,173	180,858		77,3

Fig. 1I   Peritoneal neutrophils from wild-type and *PAR2* $^{-/-}$ C57BL6/J mice were incubated with trypsin and catG (0.25 μM).

-	trypsin	cat G	-	trypsin	cat G
35,2	102,8	10,4	13,9	41,4	9,5
42,8	142,7	10,3	19,9	9,4	8,4

Fig. 2. NET formation is triggered by PAR2-activating FXa.

Fig. 2B The amount of extracellular DNA released by neutrophils 3 h post-incubation with FXa at the indicated concentrations (µM) based on SytoxGreen staining.

-	0.25	0.5	1
15,747	28,726	63,99	98,098
27,464	33,256	69,687	89,393
23,717	71,368	44,662	74,798
34,972	41,366	37,178	115,809
31,003	46,176	38,769	109,271
33,184	32,422	102,133	87,996

Fig. 2D The amount of extracellular DNA released by neutrophils 3 h post-incubation with FXa and FXa EGR native protein (active site irreversibly blocked by 1 µM of the chloromethylketone tripeptide inhibitor EGRck) based on SytoxGreen staining.

-	FXa	FXa EGR
23,266	98,098	33,5
38,486	89,393	29,199
43,129	74,798	51,825
30,57	115,809	49,172
50,573	87,996	39,638

Fig. 2E The amount of extracellular DNA released by neutrophils 3 h post-stimulation with FXa (1 µM) after pre-incubation with 100 µM FSLLRY-NH_2 for 10 min.

-	FSLLRY-NH_2	FXa	FSLLRY-NH_2+ FXa
39,062	53,46	163,608	26,635
62,929	48,373	165,095	32,254
66,225	11,691	185,303	19,126
51,867	14,741	63,394	19,695
53,632	29,361	77,913	28,991

Fig. 3. FXa induces NET formation in the liver vasculature and is dependent on PAR2 expression.

(C-E) Quantitative analysis of NETs in liver sinusoids: area (%) covered by
(C) extDNA

CONTROL	WT	PAR-2$^{-/-}$
0,02	3,269	0,017
0,081	2,108	0,016
0,066	5,03	0,078
0,065	4,173	0,122
0,094	4,567	0,031
	3,967	0,074
	1,844	0,044
	2,492	
	1,39	
	2,757	
	2,425	
	3,97	

(D) histone H2A.X

CONTROL	WT	PAR-2$^{-/-}$
1,491	16,295	1,22
2,983	13,102	0,255
0,901	11,847	2,376
0,805	13,865	0,35
0,977	15,541	0,67
1,492	12,577	1,128
1,162	11,907	0,693
0,528	9,839	0,391
0,373	10,252	0,388
1,105	9,859	1,169
0,565	10,282	1,454
1,547	18,748	1,077
0,674	14,827	0,909
	12,791	0,835
	15,562	1,823
	12,663	0,994
	9,389	1,915
	11,859	1,247
	15,949	1,287
	10,323	0,653
	8,783	1,596
	9,373	1,623
	10,491	1,602
	11,379	
	11,308	
	12,813	
	10,373	
	11,634	
	13,659	
	11,894	
	16,301	

(E) neutrophil elastase (NE)

CONTROL	WT	PAR-2$^{-/-}$
2,985	19,356	1,821
1,844	18,374	2,28
1,198	15,697	2,6
1,105	16,203	1,52
1,12	19,332	0,792
2,059	14,041	2,423
1,426	16,219	1,438
1,173	11,975	2,91
1,79	14,505	2,16
1,724	16,783	1,265
1,25	15,789	1,574
1,627	16,674	2,901
1,67	18,057	1,949
	24,08	2,592
	15,89	2,534
	14,781	2,465
	14,867	2,287
	15,346	1,907
	9,722	3,357
	12,242	1,911
	8,822	1,549
	9,952	2,988
	13,302	2,574
	17,844	
	15,431	
	12,294	
	15,321	
	10,181	
	12,411	
	13,823	
	12,846	

Fig. 4. Induction of coagulation by RgpA in the liver vasculature is associated with NET formation.

(B–D, F–H) Quantitative analysis of NETs in the liver sinusoids: area (%) covered by (B, F) extDNA, (C, G) histone H2A.X, and (D, H) neutrophil elastase (NE).

Fig. 4B	CONTROL	RgpA	RgpA / Kyt
	0,191	2,931	0,093
	0,044	1,935	0,078
	0,072	2,14	0,035
		1,834	
		1,762	
		1,884	
		1,645	

Fig. 4C	CONTROL	RgpA	RgpA / Kyt
	1,947	11,146	2,123
	1,897	9,874	1,231
	1,914	11,896	2,318
		13,808	
		9,773	
		21,057	
		10,49	
		12,905	
		12,055	
		8,843	
		11,538	
		12,05	

Fig. 4D	CONTROL	RgpA	RgpA / Kyt
	2,179	13,536	2,228
	2,843	15,401	2,397
	2,084	13,012	2,925
		12,823	
		10,735	
		15,955	
		11,549	
		16,833	
		12,641	
		14,59	
		12,747	
		10,913	

Fig. 4F	DMSO	-	Apx
	2,58	2,931	0,101
	2,899	1,935	0,027
	2,555	2,14	0,113
	1,806	1,834	0,133
		1,762	0,075
		1,884	
		1,645	

Fig. 4G	DMSO	-	ApX
	11,103	11,146	2,325
	9,911	9,874	2,063
	9,555	11,896	1,357
	10,083	13,808	1,643
	9,148	9,773	1,192
	11,819	21,057	1,371
	11,762	10,49	2,13
	14,345	12,905	2,414
	12,301	12,055	1,948
		8,843	1,722
		11,538	0,973
		12,05	1,009
			0,941
			1,061
			1,43
			1,479

Fig. 4H	DMSO	-	Apx
	12,831	13,536	3,32
	11,033	15,401	2,404
	19,493	13,012	1,856
	11,631	12,823	2,969
	12,485	10,735	2,204
	18,724	15,955	2,468
	11,399	11,549	0,987
	9,242	16,833	1,163
	9	12,641	1,969
		14,59	2,009
		12,747	2,295
		10,913	1,411
			1,234
			0,888
			1,975
			1,07

Fig. 5. Biochemical and functional characterization of protease-derived NETs.

Fig. 5A Calcium mobilization in neutrophils exposed to AC-264613, trypsin, and/or FXa.

-	AC 264613	trypsin	FXa
43,26	58,32	86,94	395,7
35,16	85,74	115,68	325,2
19,56	77,58	97,74	267,66
41,16	89,52	59,58	
55,44	74,46	65,28	
66,06	50,28		
59,82	78,54		

Fig. 5B The amount of extracellular DNA estimated by SytoxGreen staining in neutrophils pre-treated for 5 min with the ERK1/2 inhibitor UO126 before stimulation with AC-264613, trypsin and FXa for 3 h.

-	UO126	-	UO126	-	UO126	-	UO126
81,075	54,128	114,829	85,529	218,755	116,356	163,608	106,469
35,166	60,4	100,788	80,898	177,522	89,411	165,095	114,658
36,933	100,332	109,92	90,615	168,331	134,578	185,303	117,282
59,91	35,087	95,039	30,402	166,419	129,847	166,686	74,613
64,522	28,504	100,681	26,438	140,977	128,336	106,854	91,976
65,56	97,223	146,92	28,658	197,635	57,196	107,251	63,892
53,775	114,44	111,386	125,995	313,109	54,407	91,544	61,554
30,717	60,893	171,884	150,808	263,976	144,611	131,413	81,514
29,173	84,862	170,903					57,077
24,459	68,615	128,706					73,769
25,477	63,306	180,858					
24,345	29,647	181,986					
78,36		145,9					

Fig. 5D Formation of NETs released by neutrophils isolated from 3 independent donors. The amount of extracellular DNA released by neutrophils 3 h post-incubation with AC264613 (100 µM) based on SytoxGreen staining (RFU = relative fluorescence units) (lower panel).

-	AC264613	-	AC264613	-	AC264613
141,347	1321,609	88,869	969,069	175,074	814,756
128,128	1200,662	144,622	1010,971	85,321	909,378
133,9	1096,06	37,477	1107,034	30,977	876,101
172,714		31,047		261,202	
137,276		132,538		56,764	
155,85					
153,626					

Fig. 5J Bactericidal activity of NETs induced by PAR2 activators. *E. coli* cultures were mixed with NETs generated by exposure to PMA (25 nM), AC-264613 (100 µM) and FXa (1 µM) or serum-free DMEM collected from neutrophils. After incubation for 2 h, the bacteria were plated and the number of colonies (CFUs) was determined.

-	PMA	AC 264613	FXa	-	PMA	AC 264613	FXa
10	10	10	10	10	0	10	10
21	14	18	11	10	1	6	13
22,3	13,6	19,2	14,2	9,6	1,1	5,4	15,5
10	13	10	20	10	0	10	10
19	12,4	14	22	11	1	7	16
26,7	3	13,6	17,9	11,8	1	5,8	16,8
9	3,7	7	20	20	1	10	10
9,5	4,42	7,6	16	21,8	0,8	13	18
8,72	4	6,36	13,6	17	1,31	14,3	17,4
10	3,2	10	20	18,7	1,4	10	20
10,4	5,54	15	20	18	1,17	14	20
11,08	8,6	11,9	22,4	19,4	0	15,3	19,5
10	8	10,68		20	4		
19	5,4			21	3,2		
18,6	10			20,1	3,5		
10	8						
10	7,8						

Fig. 5L Enzymatic activity of human neutrophil elastase

-	PMA	AC 264613	trypsin	FXa
95,1104602	205,36	21,2118326	35,1155875	47,9158347
32,922018	230,818613	16,3950282	35,0141721	53,3344261
56,6733262	58,7299062	25,9312689	43,9912161	45,9402559
44,1552461		13,8666632	47,5893438	

Fig. 5M Enzymatic activity of cathepsin G

-	PMA	AC 264613	trypsin	FXa
0,06206429	0,294	0,04761085	0,16453145	0,12726481
0,11201065	0,355	0,04759609	0,0878284	0,07509332
0,27746001	0,41314917	0,24896517	0,34077454	0,51679081
0,1277215	0,36055914	0,16216832		

Fig. S1. Identification of PAR-2 protein on the surface of neutrophils.

The presence of PAR-2 receptor on the surface of **(A)** human and **(B)** murine neutrophils was detected with anti-PAR2 antibody and goat anti rabbit IgG conjugated with APC.
After fixation and permeabilization cells were stained with PAR-2 specific antibodies. Additionally, trypan blue quenchinh **(Q)** was performed to confirm the localization of the receptor.

A) Human PMN

Unstained cells - AF

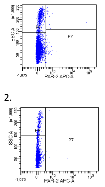

Population	% Parent
P7_1	0,80
P7_2	0,1

PAR-2 ab.

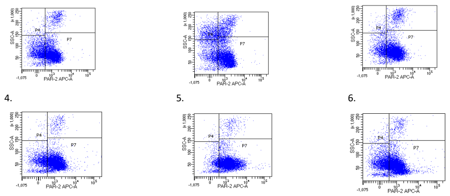

	Population	% Parent
Donor 1	P7_1	70,8
	P7_2	68,4
Donor 2	P7_3	80,7
	P7_4	79,9
Donor 3	P7_5	90,1
	P7_6	67,2

2nd ab.

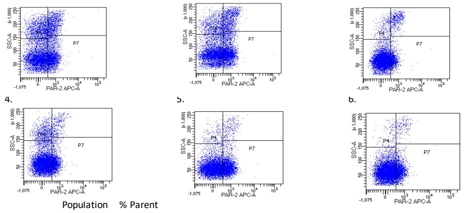

	Population	% Parent
Donor 1	P7_1	36,2
	P7_2	41,8
Donor 2	P7_3	17,8
	P7_4	20,3
Donor 3	P7_5	35,1
	P7_6	21,3

Quenching (Q)

Unstained cells - AF

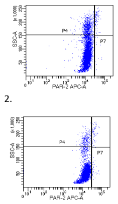

Population	% Parent
P7_1	1,20
P7_2	1,3

PAR-2 ab.

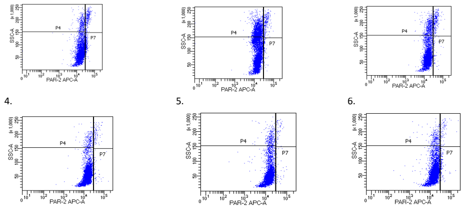

	Population	% Parent
Donor 1	P7_1	7,7
	P7_2	4
Donor 2	P7_3	4,4
	P7_4	7,7
Donor 3	P7_5	4
	P7_6	4,4

2nd ab.

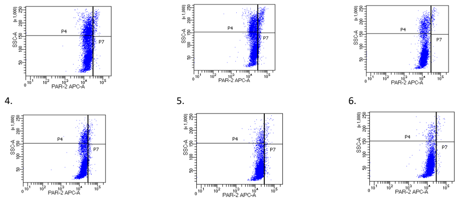

	Population	% Parent
Donor 1	P7_1	4,3
	P7_2	11,2
Donor 2	P7_3	1,5
	P7_4	4
Donor 3	P7_5	3,6
	P7_6	1,2

B) Murine neutrophils

Unstained cells - AF

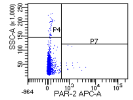

Population	% Parent
P7	0,2

PAR-2 ab.

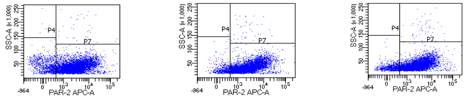

	Population	% Parent
mouse 1	P7_1	87,3
mouse 2	P7_2	92,8
mouse 3	P7_3	88,6

2nd ab.

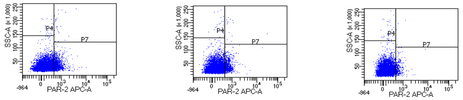

	Population	% Parent
mouse 1	P7_1	15,7
mouse 2	P7_2	12,2
mouse 3	P7_3	4,8

Quenching (Q)

Unstained cells - AF

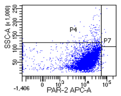

Population	% Parent
P7	0,7

PAR-2 ab.

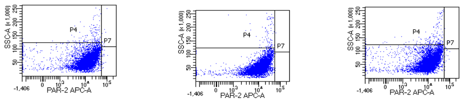

	Population	% Parent
mouse 1	P7_1	0,7
mouse 2	P7_2	0,2
mouse 3	P7_3	0,3

2nd ab.

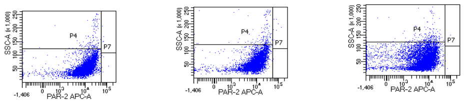

	Population	% Parent
mouse 1	P7_1	0,2
mouse 2	P7_2	0,1
mouse 3	P7_3	0,1

Fig. S2. Generation of FXa by Russell viper venom (RVV-X) and gingipain RgpA leads to the formation of NETs *in vitro* .

(C) Induction of NETs by FX (1 µM) modified using RVV-X (1 µg/ml). After incubation for 3 h, NET formation was determined by SytoxGreen staining.

-	FX	-	FX
25,133	22,336	38,029	73,291
27,683	22,582	29,011	48,734
33,03	32,217	33,975	61,506
35,758	38,6	32,542	61,741
33,302		32,526	

(D) The amount of extracellular DNA, measured by SytoxGreen staining, released by neutrophils 3 h post-incubation with 10 nM RgpA and/or 1 µM FX in the presence or absence of Kyt-1 at a final concentration of 1 µM.

FX	-	-	-	+	+	+	-	+
RgpA	-	+	+	-	+	+	-	+
Kyt-1	-	-	+	-	-	+	+	+
	39,331	59,044	54,51	46,1	132,056	84,626	43,039	47,382
	39,016	57,269	43,108	33,389	141,856	39,998	44,745	45,92
	39,169	57,908	52,022	41,891	130,792	107,852	48,292	45,921
	45,029							38,701
	43,002							47,42
	49,541							43,089

(E) Kinetic measurement of clot formation after PMA- and FXa- derived NETs and FXa

NETs FXa	FXa
14,97	14,28
18,75	11,908
17,442	13,497

Fig. S3. Apixaban has no effect on the activity of gingipain RgpA.

RgpA 50nM	RgpA 50nM+Kyt-1	RgpA 50nM+Apx	-	Kyt-1	Apx
15,374	0,024	15,442	0,003	0,032	0,014
15,46	0,047	15,772	0,007	0,007	0,015

Fig. S5. Purity of PMN isolated from human blood and murine neutrophils isolated from peritoneum and bone marrow determined by flow cytometry.
(A) The purity of human PMN fraction was determined using cell specific antibodies anti-human CD15 (neutrophils), anti-human CD14 (monocytes), anti-human CD3 (lymphocytes).
(B) Purity of peritoneal neutrophils was determined using anti-mouse Ly6G-FITC antibodies.
(C) Purity of bone marrow neutrophils was determined using anti-mouse Ly6G-FITC and CD11b-PE antibodies.

A) Purity of human PMN fraction

Donor 1

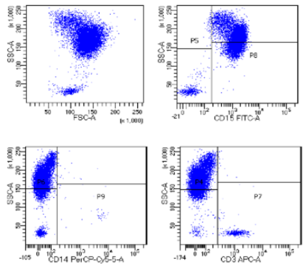

Population		% Parent
PMN	P8	93,4
monocytes	P9	0,5
lymphocytes	P7	4,7

Donor 2

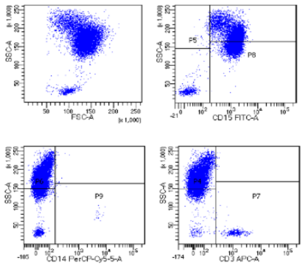

Population		% Parent
PMN	P8	94,4
monocytes	P9	0,5
lymphocytes	P7	3,6

Donor 3

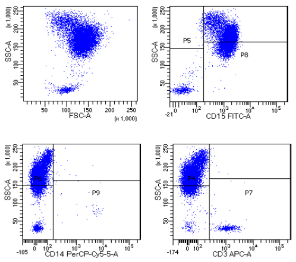

Population		% Parent
PMN	P8	94,4
monocytes	P9	0,5
lymphocytes	P7	3,6

B) Purity of murine peritoneal PMN

Donor 1

1. 2.

AF **+ FITC-Ly6G antibody**

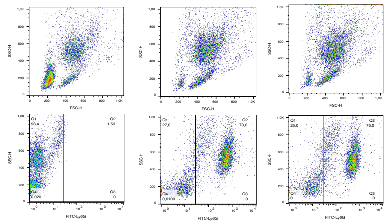

	Population	% Parent
1.	FITC-Ly6G+	73
2.	FITC-Ly6G+	75

Donor 2

1.

AF **+ FITC-Ly6G antibody**

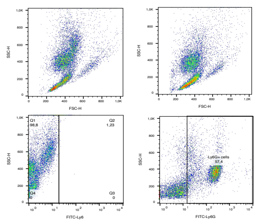

	Population	% Parent
1.	FITC-Ly6G+	57,4

Donor 3

1. 2.

AF **+ FITC-Ly6G antibody**

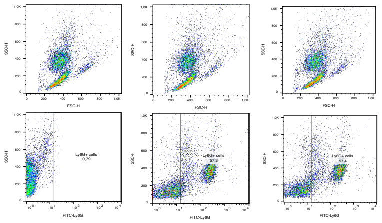

	Population	% Parent
1.	FITC-Ly6G+	57,3
2.	FITC-Ly6G+	57,4

C) Purity of murine bone marrow PMN

Donor 1

1. 2.

AF **+ FITC-Ly6G/ PE-CD11b antibody**

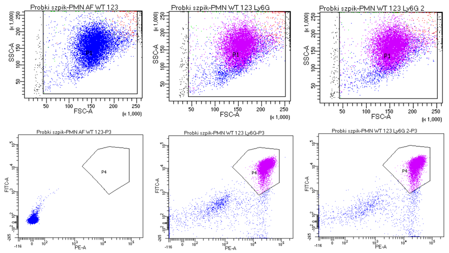

	Population	% Parent
1.	P4	81,5
2.	P4	85,6

Donor 2

1. 2.

+ FITC-Ly6G/ PE-CD11b antibody

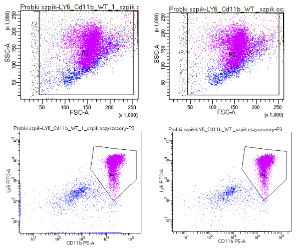

	Population	% Parent
1.	P4	77,7
2.	P4	80,9

Donor 3

1. 2.

+ FITC-Ly6G/ PE-CD11b antibody

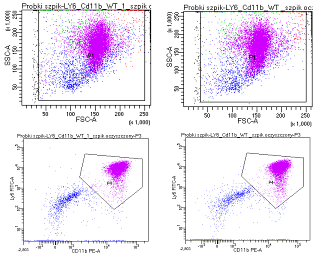

	Population	% Parent
1.	P4	83
2.	P4	85,7
