# Supplementary figures and images for: Non-classical neutrophil extracellular traps induced by PAR2-signaling proteases
Source: Cell Death Dis. 2025 Feb 19;16(1):109. doi: 10.1038/s41419-025-07428-z (PMC11840154; doi:10.1038/s41419-025-07428-z)

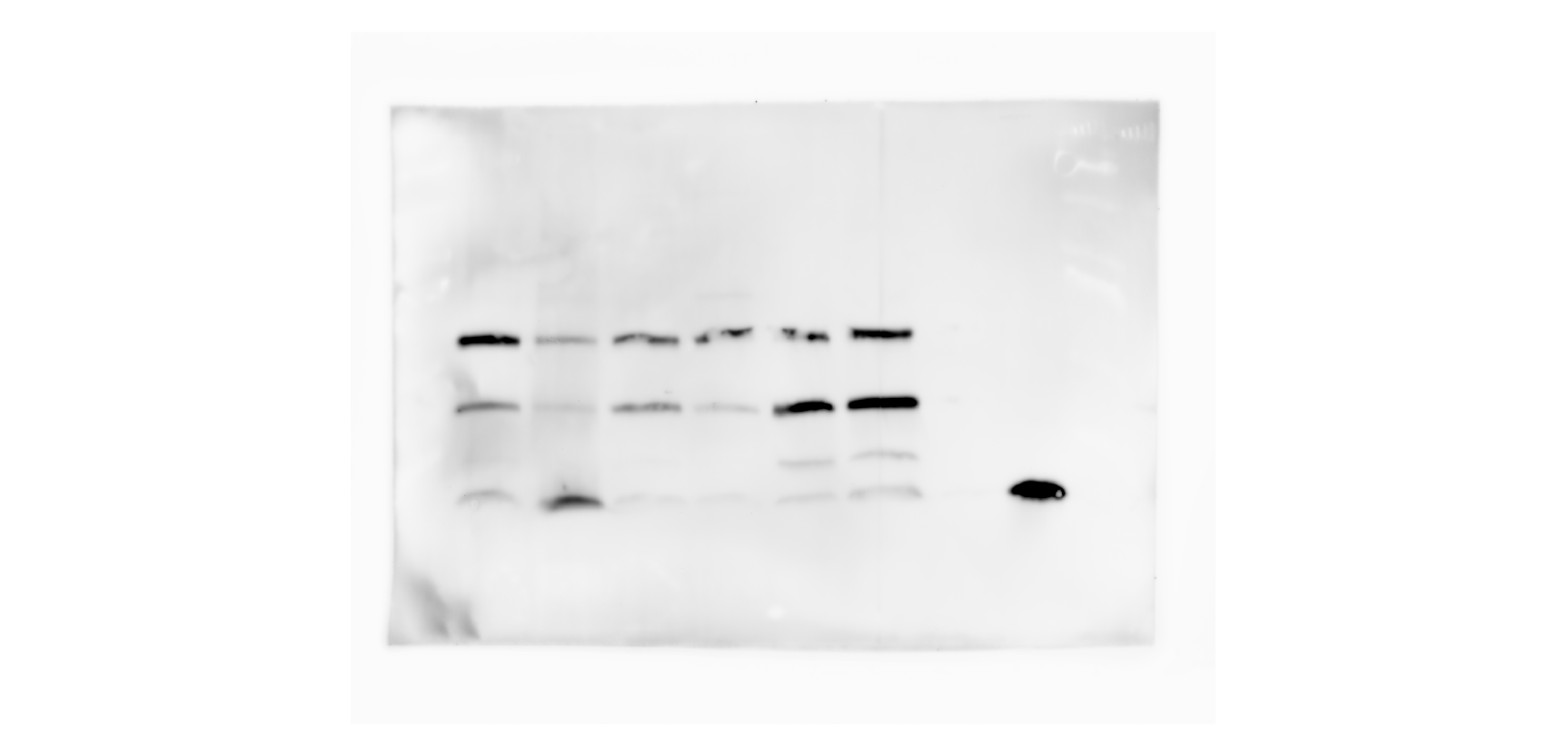

Supplement: Supplementary file 3 — original western blot file [file 41419_2025_7428_MOESM3_ESM.tif]

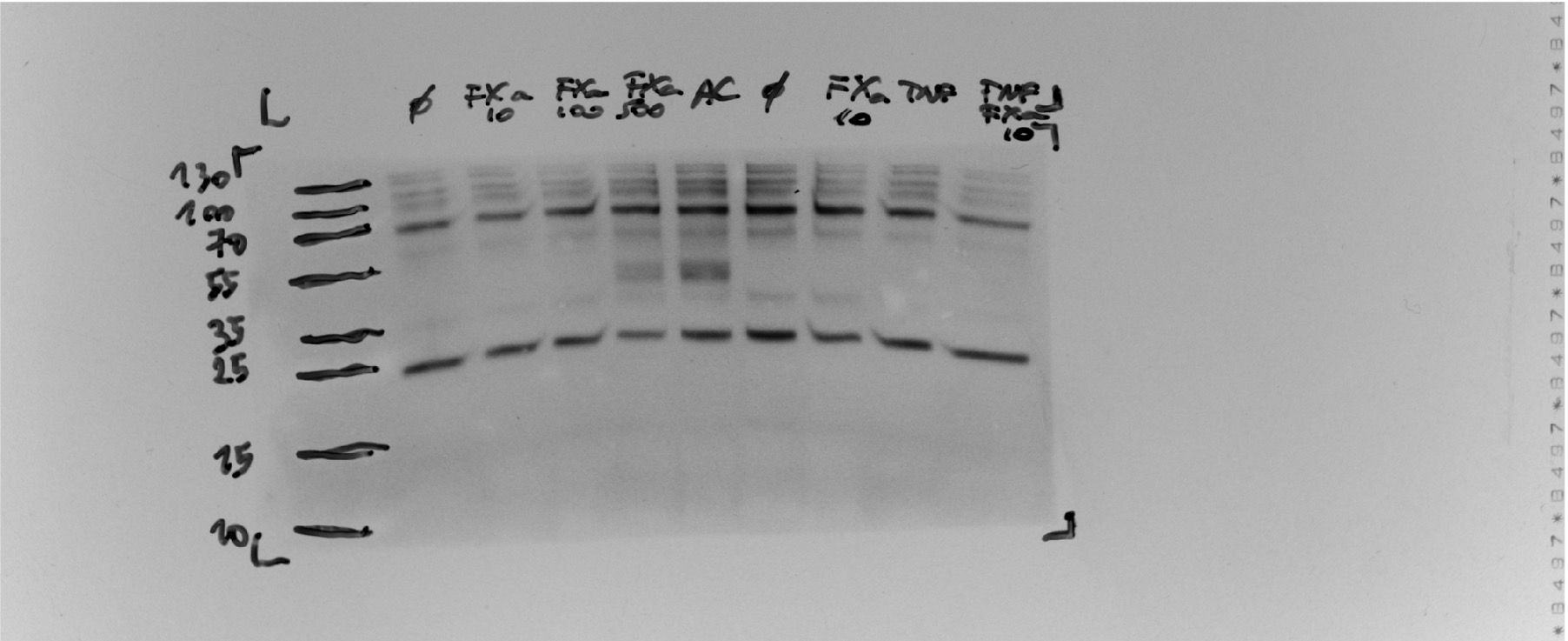

Supplement: Supplementary file 4 — original western blot file [file 41419_2025_7428_MOESM4_ESM.jpg]

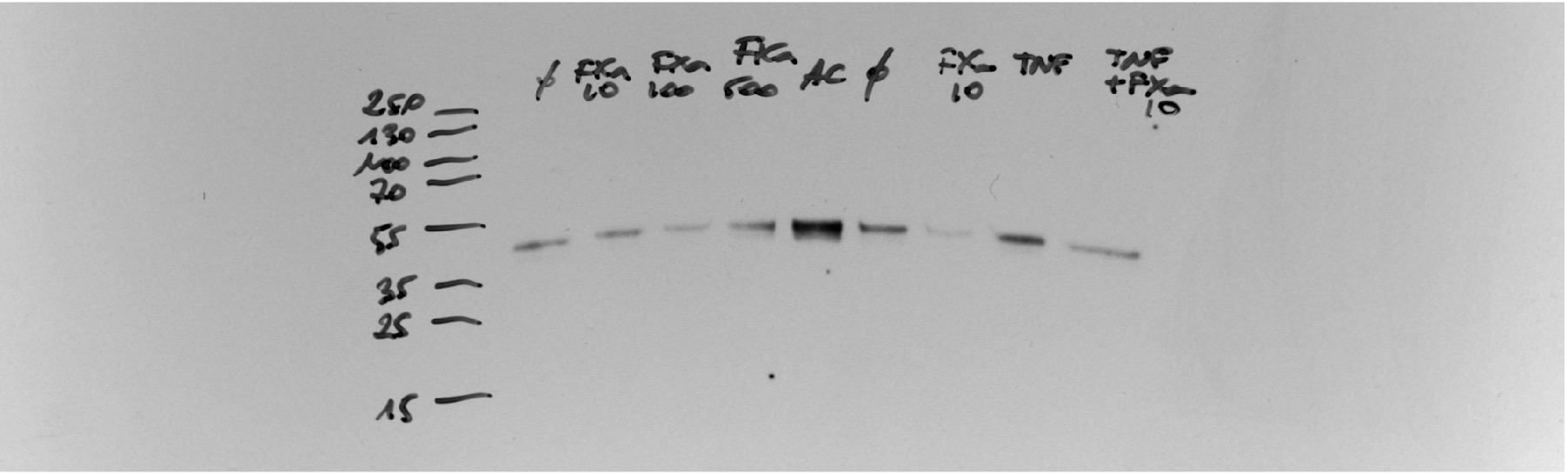

Supplement: Supplementary file 5 — original western blot file [file 41419_2025_7428_MOESM5_ESM.jpg]

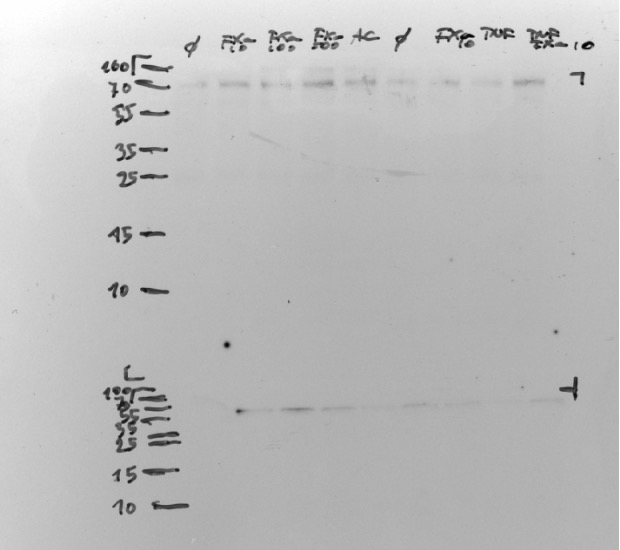

Supplement: Supplementary file 6 — original western blot file [file 41419_2025_7428_MOESM6_ESM.jpg]

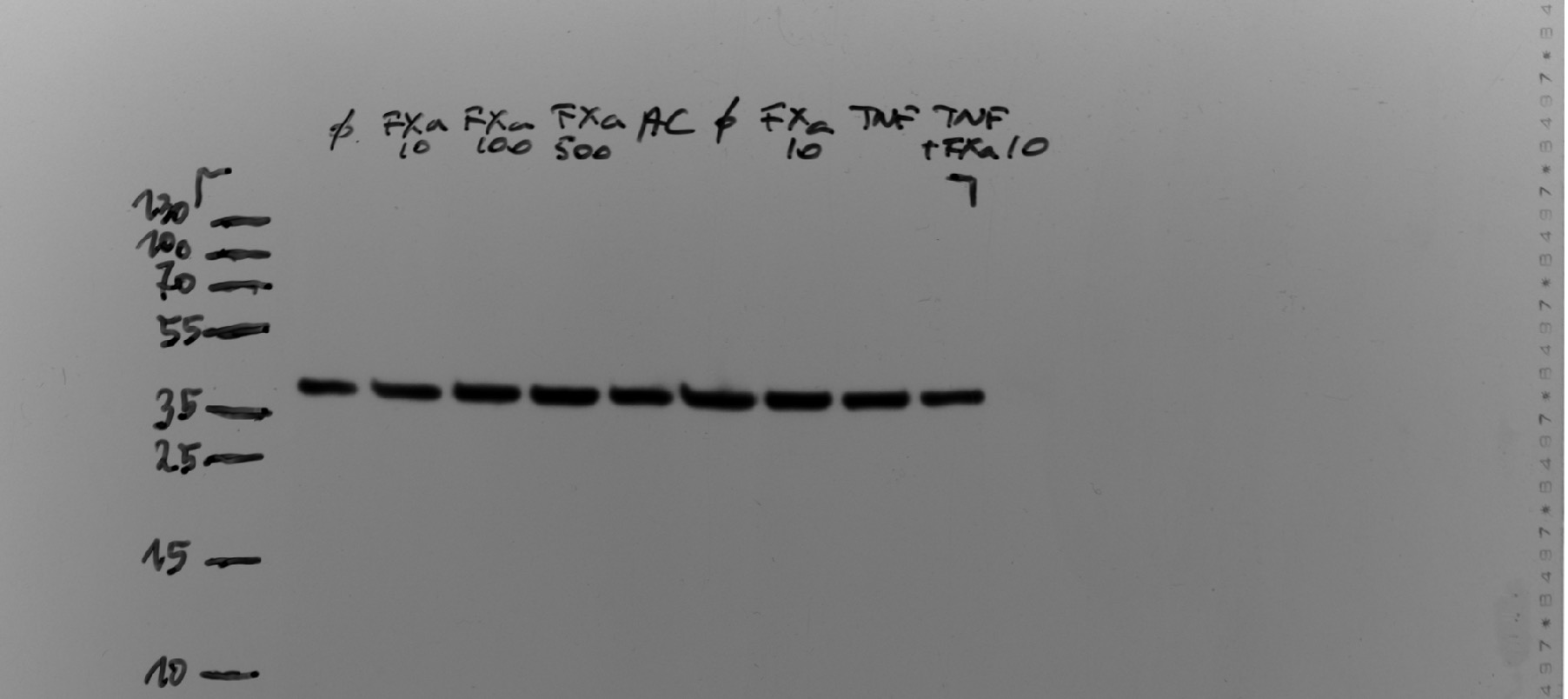

Supplement: Supplementary file 7 — original western blot file [file 41419_2025_7428_MOESM7_ESM.jpg]
